# State-of-the-art transition metal-catalyzed approaches to quinoline frameworks *via* multicomponent reactions

**DOI:** 10.1039/d5ra06627a

**Published:** 2025-12-04

**Authors:** Mosstafa Kazemi, Ghada Al Assi, Ramin Javahershenas, Rekha M M, Shaker Al-Hasnaawei, Subhashree Ray, Amrita Pal, Renu Sharma

**Affiliations:** a a, Department of Chemistry, Young Researchers and Elite Club, Tehran Branch, Islamic Azad University Tehran Iran mosstafakazemi@gmail.com; b Department of Medical Laboratory Sciences, Faculty of Allied Medical Sciences, Hourani Center for Applied Scientific Research, Al-Ahliyya Amman University Amman Jordan galasi@ammanu.edu.jo; c Organic Development, Chemistry Faculty, Urmia University Urmia Iran jshbco@yahoo.com jshbco@gmail.com; d Department of Chemistry and Biochemistry, School of Sciences, JAIN (Deemed to be University) Bangalore Karnataka India mm.rekha@jainuniversity.ac.in; e College of Pharmacy, The Islamic University Najaf Iraq; f Department of Medical Analysis, Medical Laboratory Technique College, The Islamic University of Al Diwaniyah Al Diwaniyah Iraq shakeralhasnawi@iunajaf.edu.iq; g Department of Biochemistry, IMS and SUM Hospital, Siksha ‘O’ Anusandhan (Deemed to be University) Bhubaneswar Odisha-751003 India subhashreeray@soa.ac.in; h Department of Chemistry, Sathyabama Institute of Science and Technology Chennai Tamil Nadu India amritapal.chemistry@sathyabama.ac.in; i Department of Chemistry, University Institute of Sciences, Chandigarh University Mohali Punjab India drrenusharma01@outlook.com

## Abstract

Quinoline derivatives are vital nitrogen-containing heterocycles that are widely utilized in pharmaceuticals, agrochemicals, and materials science. Recent advancements have highlighted the effectiveness of multi-component reactions (MCRs) as efficient and versatile strategies for constructing these complex molecules, offering high atom economy, operational simplicity, and broad structural diversity. Transition metal catalysts such as palladium, copper, iron, and silver have been instrumental in optimizing quinoline synthesis *via* MCRs, significantly enhancing the reaction efficiency, selectivity, and scope. These catalysts facilitate key transformations, including cyclization, C–C and C–N bond formation, and oxidative coupling under mild conditions. This review offers a comprehensive overview of the state-of-the-art transition metal-catalyzed MCR approaches for quinoline construction, emphasizing the reaction mechanisms, catalytic performance, and developments in sustainable methodologies. Additionally, it discusses future directions for catalyst design and expands on the synthetic potential of these methodologies to meet emerging scientific and industrial needs.

## Abstract 2

Quinoline derivatives are crucial nitrogen-containing heterocycles in pharma, agrochemicals, and materials science, with recent advances highlighting multi-component reactions (MCRs).

## Graphical abstract

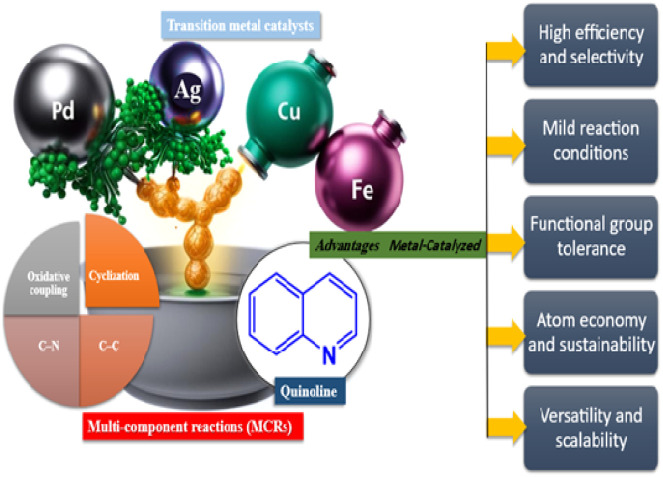

## 1 Introduction

Catalysts are fundamental elements in the landscape of chemical reactions, profoundly accelerating the rate at which these reactions occur through their dynamics. What distinguishes catalysts is their ability to engage in these processes without experiencing any permanent alteration to themselves; they facilitate reactions but remain unchanged at the end of the reaction cycle.^1^ This characteristic is crucial because it means that a single catalyst molecule can be used repeatedly, making it a cost-effective tool in both industrial and laboratory settings. The operational mechanism of catalysts involves the introduction of an alternative pathway for the chemical reaction to occur, which typically requires a lower energy threshold for initiation than the original reaction route. This threshold is referred to as the activation energy.^2^ By effectively lowering the activation energy barrier, catalysts enable reactions to occur more rapidly and efficiently, often under milder conditions than those that would generally be necessary.^3,4^ This property is particularly beneficial as it not only enhances productivity but also often leads to more favorable environmental impacts by allowing reactions to occur at lower temperatures and pressures.

The significance of catalysts extends deeply into various fields, impacting industrial production processes and natural biochemical mechanisms alike.^5,6^ For instance, in industrial applications, catalysts play a crucial role by facilitating efficient syntheses of essential chemicals, pharmaceuticals, and energy sources like fuels.^7^ By minimizing the energy input needed for these reactions, catalysts lead to notable economic savings, enhanced operational efficiency, and reductions in environmental footprints due to fewer emissions and waste.^8,9^

The breadth of catalytic applications is vast and spans numerous industries, such as petrochemicals, pharmaceuticals, and materials science. In the petroleum sector, for example, catalysts such as platinum and zeolites are instrumental in transforming crude oil into gasoline and other high-value products through processes such as catalytic cracking and hydrocracking.^10,11^ In the pharmaceutical industry, catalysts like palladium are crucial for synthesizing complex drug molecules *via* techniques such as cross-coupling reactions, which allow chemists to create intricate structures with precision.^12,13^ Furthermore, catalysts are indispensable in the field of polymer production, where transition metal-based catalysts enable the controlled polymerization of plastics, such as polyethylene and polypropylene. This capability allows for the development of materials with specific and desirable properties, making catalysts essential for innovations in material design.^14,15^

### 1.1 Transition metals as catalysts

Transition metals are essential in chemistry as catalysts due to their unique electronic properties.^1^ With their specialized d-orbital electron arrangements, they effectively lower activation energy and accelerate various reactions.^2,3^ Their versatility makes them highly valuable in both industrial applications and research, enabling complex processes to occur more efficiently.^4^ Their significance in catalysis stems from several key characteristics:

✓ *Variable oxidation states*: Transition metals can adopt multiple oxidation states, allowing them to easily donate or accept electrons in redox reactions.^4^ This property is crucial for facilitating complex reaction pathways in both organic and inorganic chemistry.

✓ *Formation of coordination complexes*: Transition metals can form stable complexes with ligands, altering their reactivity and enabling precise control over reaction mechanisms. This ability is essential in homogeneous catalysis, such as in organometallic reactions.^5^

✓ *Activation of reactants*: Many transition metals have the ability to adsorb and activate small molecules like O_2_, H_2_, and CO, making them indispensable in catalytic processes such as hydrogenation and oxidation reactions.^6^

✓ *High reactivity with substrate molecules*: Due to their partially filled d-orbitals, transition metals can interact with reactants in ways that facilitate bond formation and cleavage, making reactions more efficient.^7^

#### 1.1.1 Advantages of using transition metals as catalysts (Fig. 1)

➢ *Lower activation energy*: Transition metal catalysts reduce the energy barrier for reactions, allowing them to proceed faster and at lower temperatures. This is especially beneficial in industrial processes where energy efficiency is crucial.^8^

➢ *Increased reaction rates*: By providing alternative reaction pathways with lower energy requirements, transition metals significantly accelerate reaction rates, leading to higher productivity in chemical manufacturing.^9^

➢ *Enhanced selectivity*: Transition metal catalysts can be finely tuned through ligand modification, allowing for high selectivity in product formation. This selectivity minimizes unwanted by-products and improves yield.^10^

➢ *Versatility in different catalytic systems*: Transition metals are effective in homogeneous catalysis (*e.g.*, organometallic catalysts like palladium in cross-coupling reactions) and heterogeneous catalysis (*e.g.*, platinum or nickel in hydrogenation reactions). Their adaptability makes them suitable for a wide range of chemical processes.^11^

➢ *Sustainability and green chemistry*: Many transition metal catalysts contribute to greener chemistry by reducing the need for harsh conditions, decreasing waste production, and enabling atom-efficient transformations. Catalysts such as iron, cobalt, and nickel are gaining popularity as environmentally friendly alternatives to precious metal catalysts.^12^

➢ *Industrial and technological applications*: Transition metals are essential in many large-scale industrial processes, including petroleum refining, polymerization, and pharmaceutical synthesis. They are also integral in emerging fields like renewable energy production, such as in fuel cells and photocatalysis.^13^

The use of transition metals as catalysts is fundamental to modern chemistry due to their efficiency, selectivity, and versatility.^14^ Their ability to lower activation energy, increase reaction rates, and enable environmentally friendly processes makes them indispensable in both academic research and industrial applications.^15^ As catalyst design continues to evolve, transition metals will play a central role in advancing sustainable and energy-efficient chemical technologies.

The ongoing objective to reduce the environmental footprint of state-of-the-art transition metal-catalyzed quinoline syntheses—especially those operating *via* multicomponent reactions (MCRs)—centers on lowering catalyst loading, maximizing turnover, and integrating greener energy inputs.^16,17^ Key strategies include designing highly active and robust catalysts with superior turnover frequencies (TOFs) and turnover numbers (TONs) under milder conditions, along with ligand engineering and optimized metal–support interactions that facilitate facile recovery and recycling.^18,19^ Deploying heterogeneous or nanostructured catalysts immobilized on recyclable supports can substantially reduce metal usage per unit of product and mitigate metal contamination in pharmaceutical contexts. Moreover, prioritizing earth-abundant metals (*e.g.*, Fe, Cu, and Mn) over precious metals, while preserving activity, selectivity, and long-term stability under scalable MCR conditions, aligns with green chemistry principles and enhances sustainability for quinoline frameworks.^20,21^

Environmental burden can be further alleviated through the intentional use of renewable energy sources and process intensification. The importance of careful solvent selection in making responsible choices in chemical synthesis is crucial. Driving reaction heating or photochemical processes with solar, wind, or other low-carbon energy sources can significantly reduce the carbon footprint of large-scale operations. Photoredox-enabled MCRs, coupled with energy-efficient light sources and solar concentrators, offer milder temperatures and access to novel reactivity that can reduce byproducts.^22–24^ In addition, careful solvent selection and reaction engineering—such as solvent-free protocols, the use of benign or recyclable solvents, and continuous-flow processing with inline purification—can minimize waste and energy expenditure. Process intensification, in particular, holds substantial potential to reduce waste generation and energy use, thereby increasing the overall efficiency and sustainability of quinoline-forming MCRs without sacrificing scope or performance. Collectively, these approaches—reduced catalyst loading, renewable energy deployment, and process intensification—provide a path toward more sustainable state-of-the-art quinoline synthesis *via* MCRs.^25,26^

### 1.2 Quinolines

Quinoline derivatives represent a crucial category of heterocyclic compounds, renowned for their diverse biological activities and significant pharmaceutical applications.^27^ Their importance cannot be overstated, as they continue to play a vital role in drug discovery and development, showcasing immense potential in addressing various health challenges.^28^ Their unique chemical structure allows them to interact with various biological targets, making them valuable in drug development. These compounds exhibit antibacterial, antifungal, antiviral, anti-inflammatory, anticancer, and antimalarial properties, among others (Fig. 2).^29^ Due to their broad-spectrum activity, quinoline derivatives have been extensively studied and developed as therapeutic agents for numerous diseases.^30^ In the pharmaceutical and medicinal fields, quinoline-based drugs are widely used for treating infectious diseases. One of the most well-known quinoline derivatives is chloroquine, which has been used for decades as an antimalarial agent.^31^ Its derivative, hydroxychloroquine, has also been used for autoimmune diseases like rheumatoid arthritis and lupus. Additionally, quinoline derivatives like ciprofloxacin belong to the fluoroquinolone class of antibiotics, which are effective against bacterial infections by inhibiting DNA gyrase and topoisomerase IV, essential enzymes for bacterial replication. Some quinoline-based drugs, such as quinapril, are used as antihypertensive agents, while others are being explored for neuroprotective and anti-Alzheimer's applications.^29–31^ Beyond infectious diseases, quinoline derivatives have shown significant promise in anticancer therapy due to their ability to interfere with DNA replication and inhibit tumor growth.^32^ Compounds like camptothecin and its derivatives (topotecan and irinotecan) are used in chemotherapy as topoisomerase inhibitors, disrupting cancer cell division.^33^ Moreover, quinoline scaffolds are being explored in drug discovery for treating neurodegenerative diseases, cardiovascular conditions, and inflammatory disorders.^30^ The diverse pharmacological potential of quinoline derivatives makes them crucial in medicinal chemistry, with ongoing research aimed at discovering new, more potent, and selective therapeutic agents.^34^

**Fig. 1:**
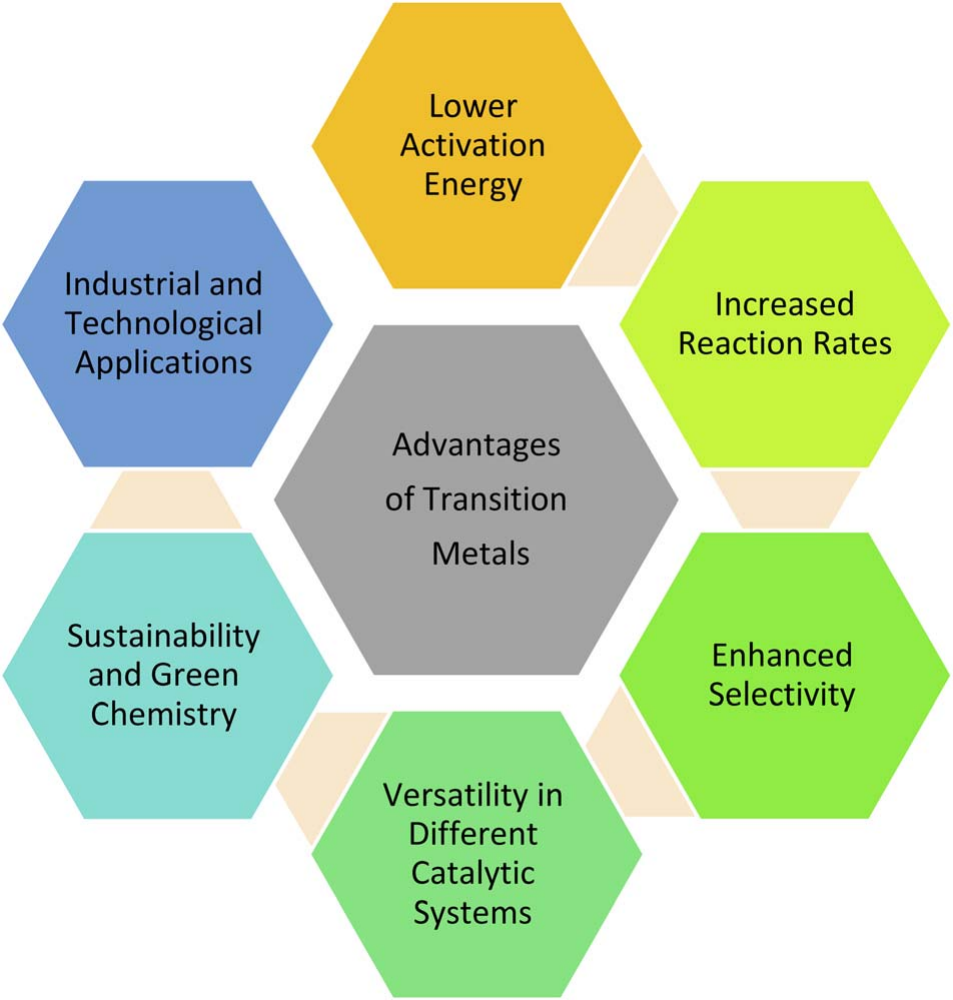
Advantages of transition metals for catalysis.

**Fig. 2:**
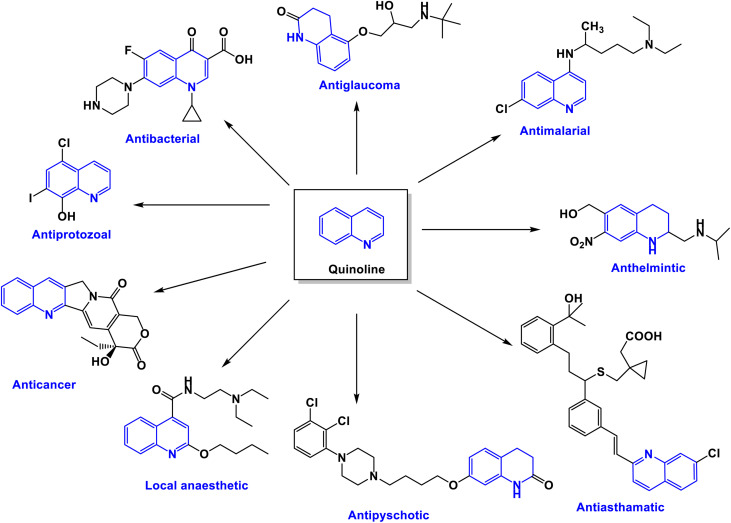
Several examples of bioactive quinoline derivatives.

#### 1.2.1 Highlights of the biological, pharmaceutical, and medicinal applications of quinoline derivatives

➢ *Broad-spectrum biological activity*: Quinoline derivatives exhibit diverse biological activities, including antibacterial, antifungal, antiviral, anti-inflammatory, anticancer, and antimalarial properties, making them valuable in drug development.^35^

➢ *Antimalarial agents*: Chloroquine and hydroxychloroquine are well-known quinoline-based drugs used for malaria treatment and autoimmune diseases like lupus and rheumatoid arthritis.^36^

➢ *Antibacterial and antiviral drugs*: Fluoroquinolones, such as ciprofloxacin and levofloxacin, are potent antibiotics that inhibit bacterial DNA replication. Some quinoline derivatives are also being explored for antiviral therapies, including potential treatments for HIV and COVID-19.^37^

➢ *Anticancer therapy*: Quinoline-based compounds like camptothecin and its derivatives (topotecan, irinotecan) are used as topoisomerase inhibitors in chemotherapy, disrupting cancer cell proliferation.^38^

➢ *Neuroprotective and anti-Alzheimer's potential*: Some quinoline derivatives show promise in treating neurodegenerative diseases by modulating enzyme activity and reducing oxidative stress in neurons.^39^

➢ *Cardiovascular and anti-inflammatory agents*: Drugs like quinapril (an ACE inhibitor) are used for hypertension treatment, while others are being studied for anti-inflammatory and analgesic properties.^40^

#### 1.2.2 Examples of drugs containing quinoline derivatives

➢ *Chloroquine & hydroxychloroquine*: Antimalarial and autoimmune disease treatments.^41^

➢ *Ciprofloxacin & levofloxacin*: Broad-spectrum fluoroquinolone antibiotics.^42^

➢ *Mefloquine*: Antimalarial drug used for prevention and treatment.^43^

➢ *Camptothecin, topotecan, & irinotecan*: Chemotherapeutic agents targeting topoisomerase enzymes in cancer treatment.

➢ *Quinapril*: An antihypertensive drug (ACE inhibitor) used to treat high blood pressure.^44^

➢ *Clioquinol*: Antifungal and antiprotozoal agent used for skin infections.^45^

➢ *Bedaquiline*: A quinoline-based drug used for multidrug-resistant tuberculosis (MDR-TB).^46^

Quinoline derivatives continue to be vital components of medicinal chemistry, with ongoing research exploring their potential in developing novel therapeutic agents for a wide range of diseases.

### 1.3 Multi-component reactions

**Fig. 3:**
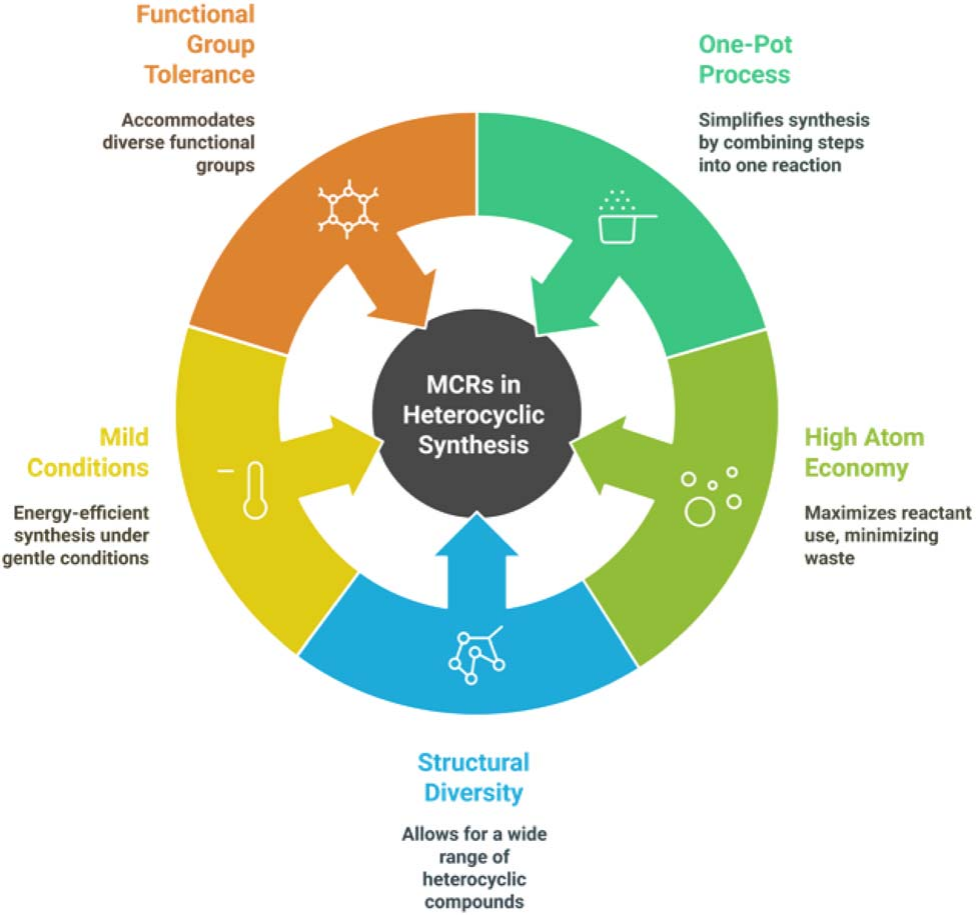
Advantages of multicomponent reactions (MCRs) in heterocyclic synthesis.

Multi-component reactions (MCRs) are highly efficient synthetic strategies that enable the construction of complex heterocyclic frameworks in a single step by combining three or more reactants in a one-pot process.^47^ The key highlights of MCRs in heterocycle synthesis include atom economy, operational simplicity, and high structural diversity, making them valuable for pharmaceutical, agrochemical, and material applications.^48,49^ These reactions often proceed under mild conditions, reducing energy consumption and minimizing waste, thus aligning with green chemistry principles.^6,50^ Additionally, MCRs allow for easy variation of substrates, providing access to a wide range of heterocycles with high functional group tolerance (Fig. 3).^6,51^ Their efficiency, reduced purification steps, and ability to generate libraries of bioactive molecules make them particularly attractive for drug discovery and industrial-scale synthesis.^52,53^

### 1.4 Highlights

➢ *One-pot process*: MCRs allow the formation of heterocyclic compounds in a single step, minimizing reaction steps and work-up procedures.^48^

➢ *High atom economy*: These reactions maximize the incorporation of reactants into the final product, reducing byproducts and waste.^54^

➢ *Structural diversity*: A wide variety of heterocyclic frameworks can be synthesized by simple variations in reactants.^55^

➢ *Mild reaction conditions*: Many MCRs proceed under mild conditions (room temperature, solvent-free, or green solvents), making them energy-efficient.^56^

➢ *Broad functional group tolerance*: They can accommodate diverse functional groups, enabling the synthesis of complex and bioactive heterocycles.^47^

### 1.5 Advantages

➢ *Efficiency & time-saving*: MCRs reduce the number of synthetic steps, reducing time, effort, and cost.^57^

➢ *Eco-friendly & sustainable*: Fewer reagents, solvents, and purification steps contribute to greener chemistry.^58^

➢ *Versatility in drug discovery*: MCRs are widely used in medicinal chemistry for the rapid synthesis of heterocyclic drug candidates.^59^

➢ *Scalability & industrial applications*: These reactions are well-suited for large-scale production in pharmaceuticals, agrochemicals, and material science.^58^

By integrating simplicity, efficiency, and sustainability, MCRs have become powerful tools for the synthesis of diverse heterocycles, revolutionizing modern organic and medicinal chemistry.

## 2 Transition metal-catalyzed MCRs in quinoline synthesis

This review presents an in-depth exploration of the latest developments in transition metal-catalyzed multi-component reactions (MCR) for the synthesis of quinolines. It delves into the intricate reaction mechanisms involved, assesses the catalytic efficiencies achieved in various methodologies, and emphasizes sustainable approaches to these reactions. The discussion extends to envisioning future perspectives that focus on enhancing catalyst design and broadening the synthetic applications of these innovative strategies.

### 2.1 Catalysis by copper

Copper, a transition metal, possesses unique electronic properties that make it an effective catalyst in various chemical transformations.^60^ It demonstrates a wide range of oxidation states, notably Cu(i) and Cu(ii), which allow it to participate in diverse redox reactions.^61^ Moreover, copper's ability to coordinate with various ligands and engage in π-backbonding contributes to its catalytic effectiveness.^62,63^ These attributes render copper particularly adaptable, facilitating a variety of coupling reactions, including those essential for the formation of heterocycles.

#### 2.1.1 Advantages of using copper as a catalyst

✓ *Cost-effectiveness*: One of the pivotal advantages of utilizing copper as a catalyst is its economic viability. Compared to noble metals such as palladium and platinum, copper is significantly more abundant and less expensive.^64^ This affordability enhances its attractiveness, especially for large-scale industrial processes where cost reduction is critical.

✓ *Environmental sustainability*: The increasing emphasis on green chemistry underscores the need for environmentally benign catalytic processes. Copper's natural abundance and non-toxic nature make it a favorable choice for environmentally sustainable practices.^65^ Reactions that utilize copper can often be performed under mild conditions, resulting in lower energy consumption and reduced carbon footprints.

✓ *Versatility in catalytic applications*: Copper's versatility is evident in its ability to catalyze a range of reactions, including cross-coupling reactions, C–H activation, and oxidation–reduction processes. Its participation in reactions such as Ullmann coupling, Sonogashira coupling, and azide–alkyne cycloaddition showcases its pivotal role in organic synthesis.^66^

✓ *Facilitation of heterocycle synthesis*: Heterocycles, which are cyclic compounds containing one or more heteroatoms (such as nitrogen, oxygen, or sulfur) within the ring, are ubiquitous in biological and pharmacological contexts.^67^ The synthesis of heterocycles often poses challenges, particularly in developing efficient, selective, and high-yielding methods. Copper catalysts have demonstrated exceptional efficacy in facilitating the formation of these complex structures.^68^

✓ *Tuning reactivity and selectivity*: The reactivity and selectivity of copper catalysts can be modulated through the choice of ligands and reaction conditions, providing chemists with the flexibility to optimize reactions for specific substrates.^69^ This control over reactivity enhances the utility of copper in complex synthesis scenarios, particularly in the development of pharmaceutical compounds, where selectivity is paramount.^70^

#### 2.1.2 The synthesis of heterocycles: a focused analysis

The synthesis of heterocycles is crucial in pharmaceutical research, as many drugs contain heterocyclic structures that are integral to their biological activity. Copper-catalyzed reactions, such as the copper-catalyzed cyclization of anilines and alkynes or the preparation of indoles from phenylacetylene and nitroalkenes, exemplify the ability of copper to facilitate the formation of these vital compounds.^71^ A noteworthy example is the copper-catalyzed synthesis of quinolines, a class of heterocycles with considerable biological significance. This often employs copper salts in conjunction with a base, yielding quinolines with excellent yields and selectivity. The ability of copper to mediate such transformations reflects its indispensable role in advancing synthetic methodologies in heterocycle chemistry.^72^ Furthermore, copper's application in the synthesis of other nitrogen-containing heterocycles, such as pyrroles and pyridines, has expanded the toolkit available for synthetic chemists. The development of copper-catalyzed reactions has led to greater accessibility and diversity of heterocyclic compounds, fostering innovation in the design of new drugs and agrochemicals. In summary, copper serves as an essential catalyst in a wide array of chemical reactions, particularly in the synthesis of heterocycles with important applications in pharmaceuticals and materials science. Its cost-effectiveness, environmental sustainability, versatility, and ability to facilitate complex reactions position copper as a catalyst of choice in both academic research and industrial applications. As the field of catalysis continues to evolve, the ongoing research into copper-catalyzed reactions promises to lead to innovative methodologies that will further advance our understanding and utilization of these fundamental chemical processes.

**Scheme 1:**
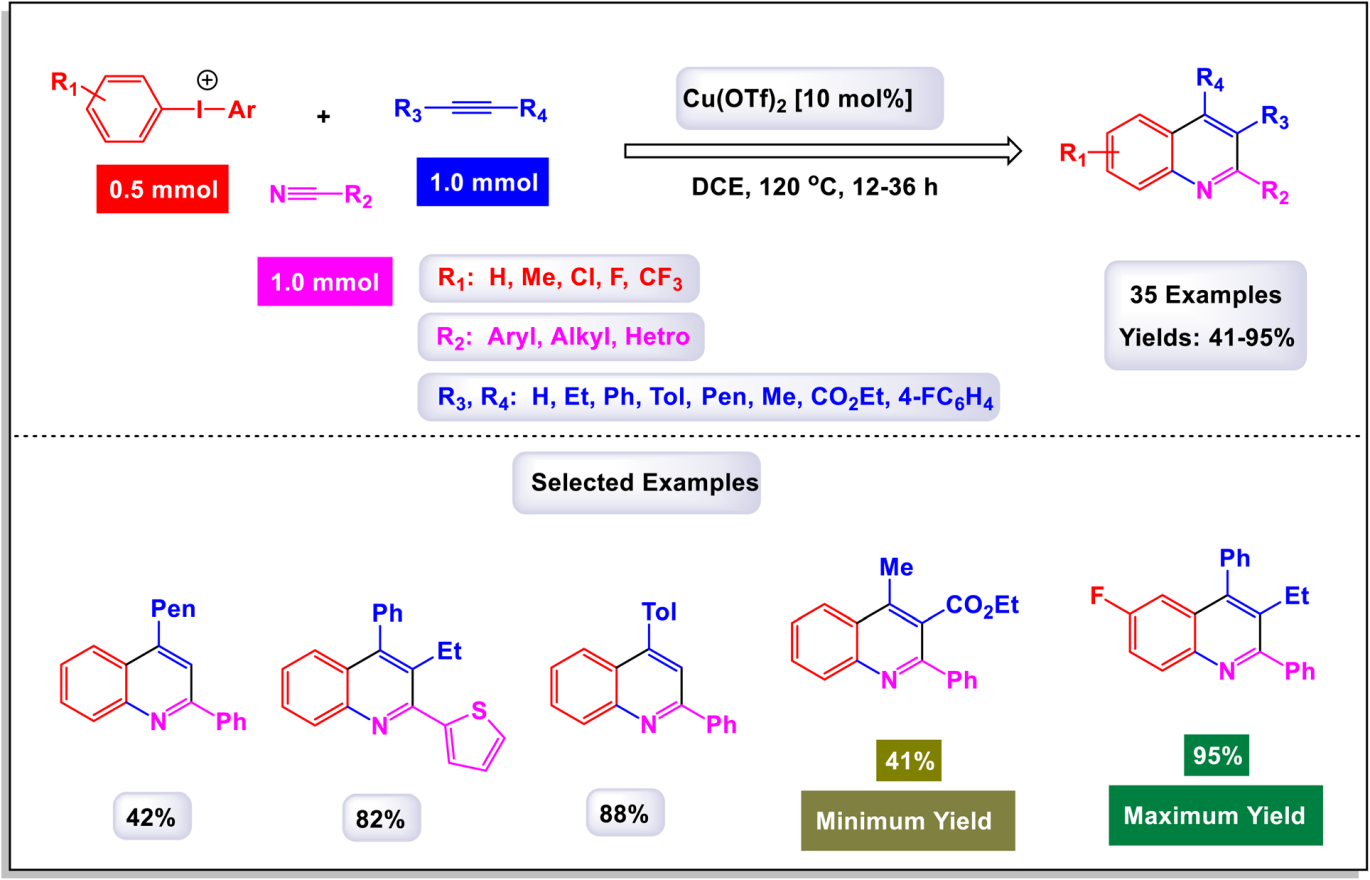
Synthesis of multiply-substituted quinolines [catalysis by Cu(OTf)_2_].

**Scheme 2:**
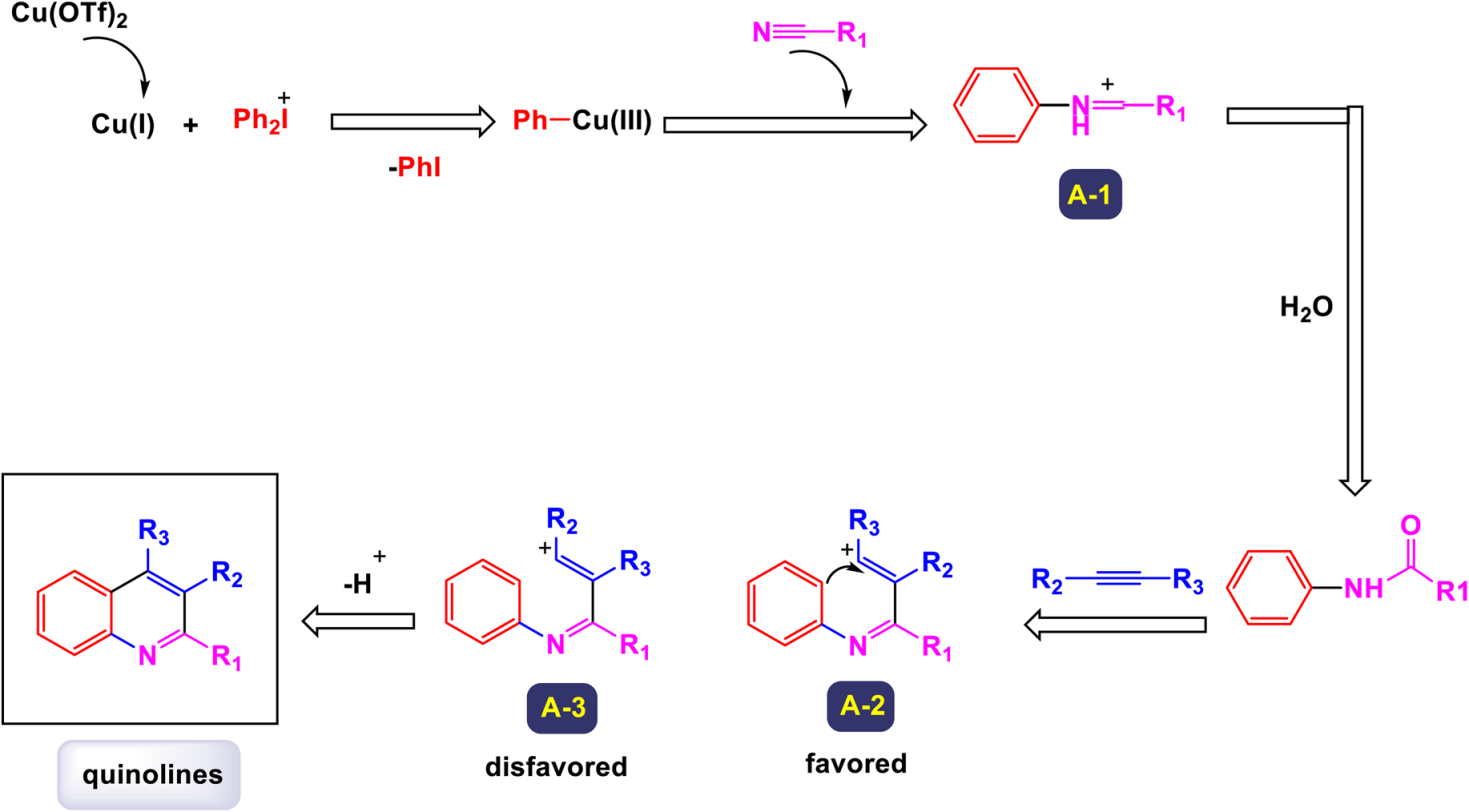
Plausible mechanism for the synthesis of multiply-substituted quinolines [catalysis by Cu(OTf)_2_].

The research team led by Chen has developed a highly efficient and regioselective method for synthesizing multiply substituted quinolines.^62^ This innovative approach involves three-component reactions that integrate diaryliodoniums, alkynes, and nitriles. Conducted in the presence of a copper catalyst, the reactions take place in 1,2-dichloroethane under thermal conditions over a duration of 12 hours, as illustrated in Scheme 1. To optimize the reaction process, the researchers conducted a thorough investigation into the effects of various factors, including the loading of the copper catalyst, the nature of the solvent, and other reaction conditions. The regioselective cyclization process was effectively catalyzed by Cu(OTf)_2_, with the aryl group derived from the diaryliodonium serving as a crucial C2 building block in the formation of the quinoline structure. This detailed study demonstrates the method's efficiency and offers key insights into optimizing reaction parameters for improved results. According to the mechanism presented by the authors (Scheme 2), the process begins with the conversion of Cu-(OTf)_2_ into Cu(i). This transformation can occur through either reduction or disproportionation, a phenomenon that has been explored in earlier studies. The formation of a purple solution serves as an indicator of the presence of Cu(i) species in the reaction mixture. Following this initial step, the Cu(i) species undergoes oxidative addition with a diaryliodonium salt, such as Ph_2_I^+^. This interaction yields a Ph–Cu(iii) species, which is key to the subsequent transfer of the phenyl group to a nitrile compound, resulting in the formation of an *N*-phenylnitrilium intermediate (designated as intermediate A-1). This intermediate is known for its high reactivity; when subjected to hydrolysis, it transforms into an anilide. *N*-phenylnitrilium is particularly notable for its ability to rapidly react with acetylenes. This reaction can lead to the formation of two different intermediates: intermediate A-2, which is favored due to electronic effects, and intermediate A-3, which is less favorable. Intermediate A-2 then undergoes an electrophilic annulation, ultimately producing the desired quinoline product. This sequence of reactions highlights the intricate interplay of various species and reactions driving the synthesis of quinoline derivatives. This innovative approach marks a significant shift from traditional methods that typically depend on condensation chemistry. By allowing for extensive variation in the substitution patterns on quinolines, it opens up new avenues for exploration and development in this field.

**Scheme 3:**
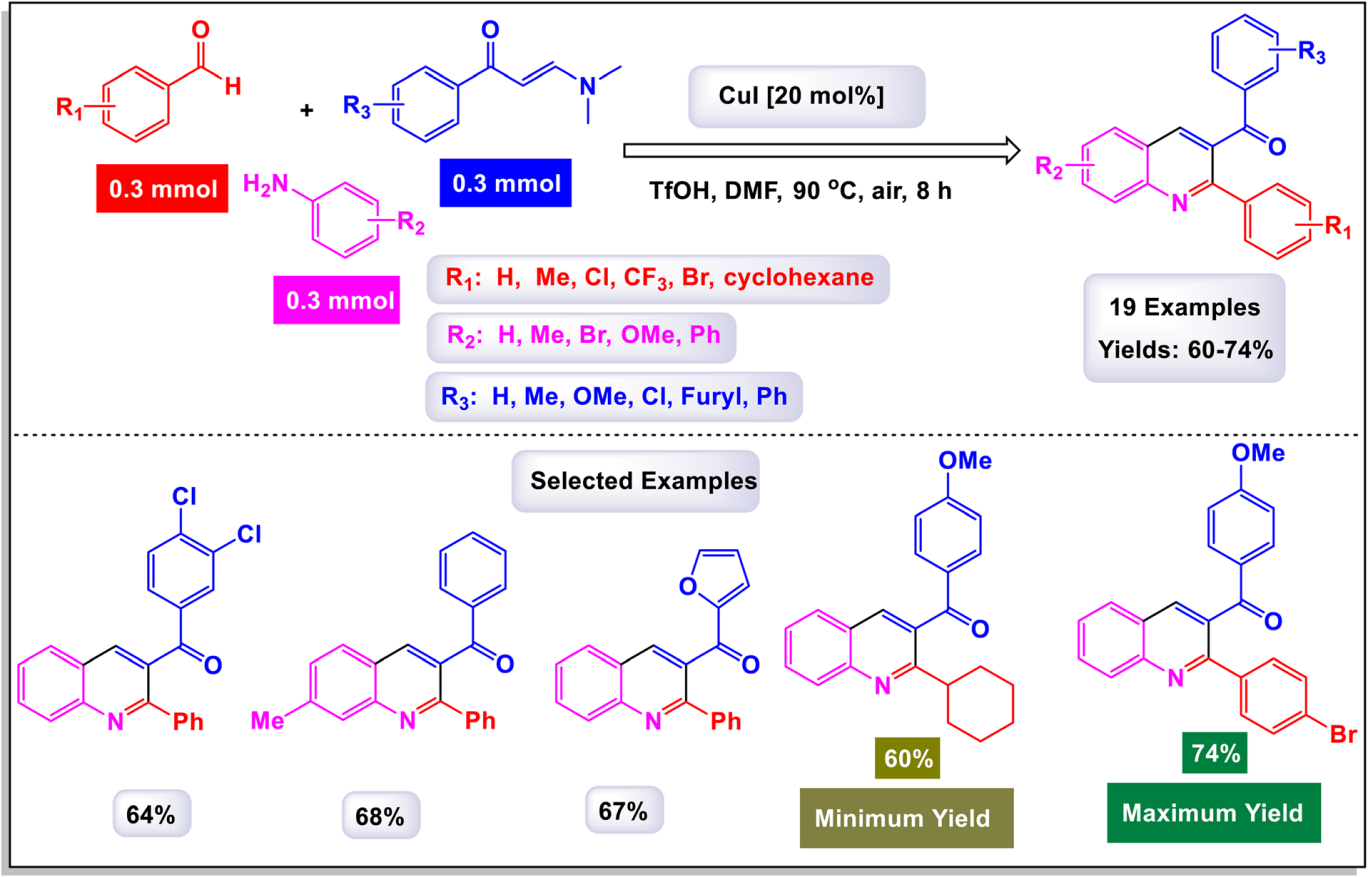
Synthesis of functionalized quinolines [catalysis by CuI].

**Scheme 4:**
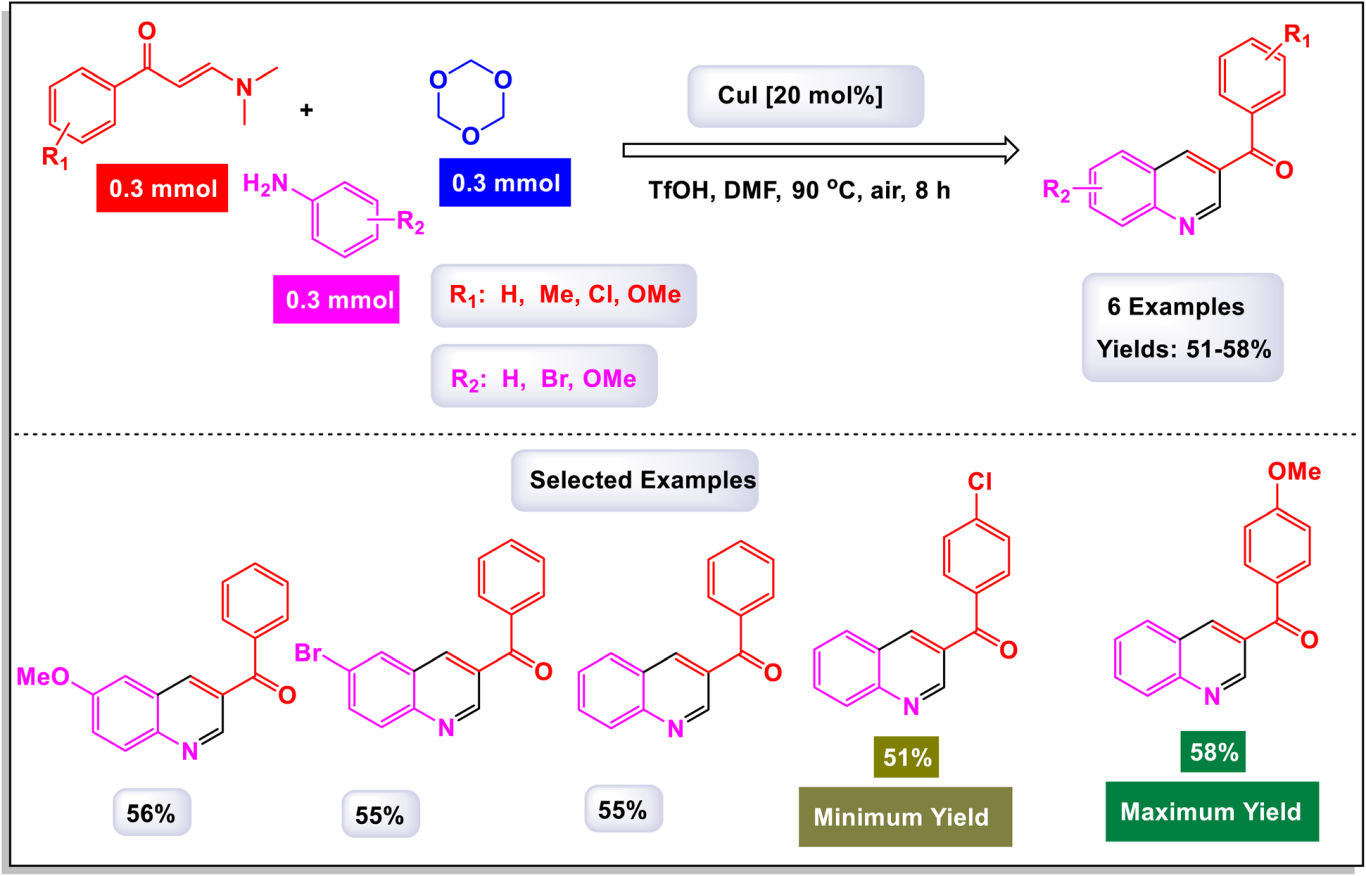
Synthesis of functionalized quinolines [catalysis by CuI].

**Scheme 5:**
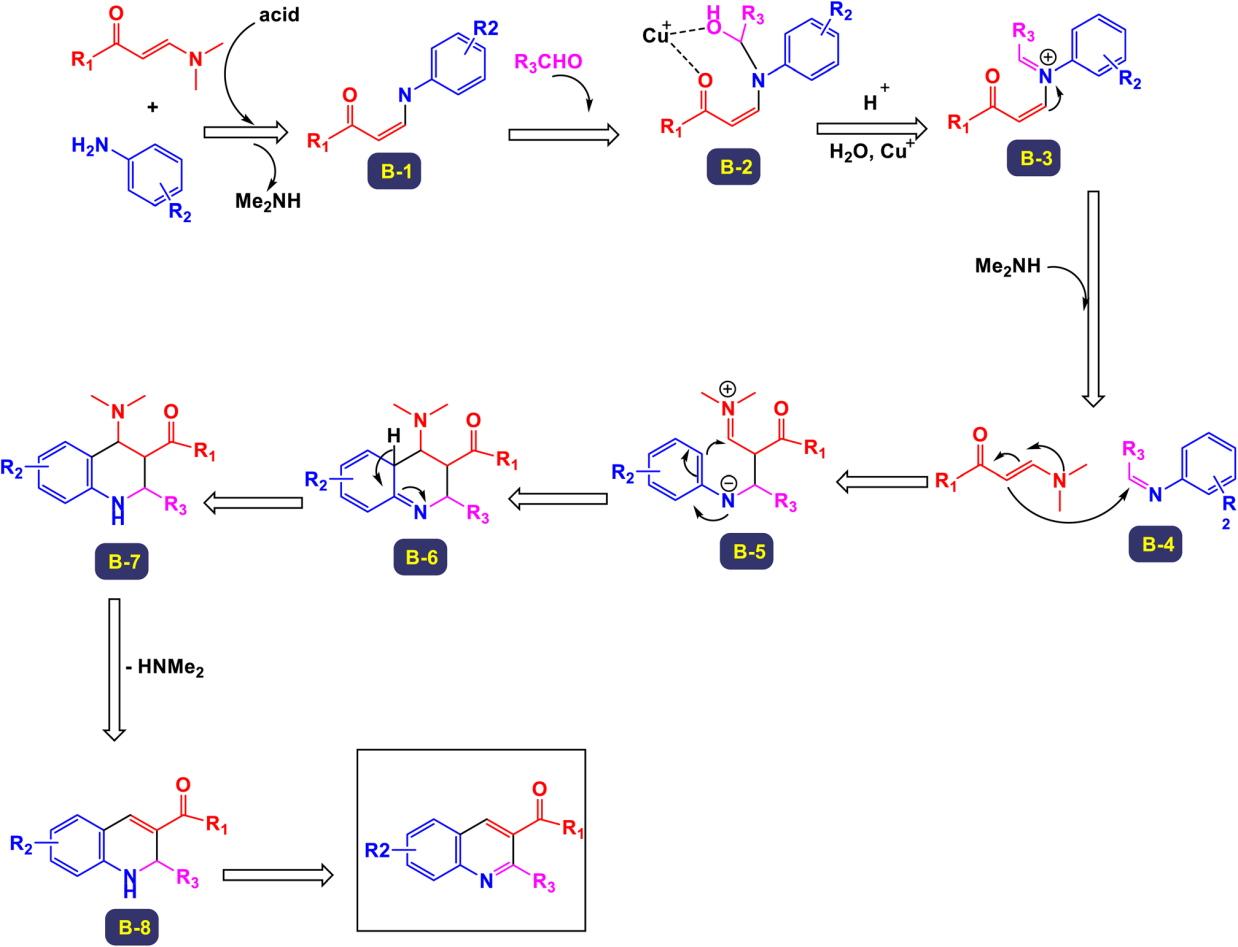
Plausible mechanism for the synthesis of functionalized quinolines [catalysis by CuI].

The research team led by Wan has successfully devised a versatile and regioselective approach for the synthesis of functionalized quinolines.^73^ This innovative method involves one-pot three-component reactions, combining enaminones, aldehydes, and anilines, all facilitated by the use of Cu(i) as a catalyst. The results of these multicomponent reactions, which are catalyzed by Cu(i) in the presence of TfOH and conducted in DMF under air conditions, are illustrated in Schemes 3 and 4. Notably, the synthesis demonstrated that both heteroaryl-functionalized enaminones and heteroaryl–aldehydes were compatible with the reaction, leading to the formation of the desired heteroaryl-functionalized quinoline products. Additionally, when cyclohexyl carbaldehyde was utilized as a substrate, the expected quinoline product was also obtained. This highlights the robustness of the methodology, showcasing its ability to tolerate a diverse range of substrates, including both aryl and alkyl aldehydes. In the mechanism proposed by the authors (illustrated in Scheme 5), transamination occurs between enaminone and aniline under acidic conditions, resulting in the formation of compound B-1. This compound then reacts with an aldehyde, catalyzing the addition of the amino group from B-1 to the aldehyde, which leads to the creation of compound B-2. Following this, a dehydration reaction transforms B-2 into the iminium cation B-3, aided by the presence of a Cu(i) salt and a proton. Considering the high reactivity of the resulting imine in generating quinolines, it is plausible that imine B-4 arises from the breakdown of iminium cation B-4 in conjunction with the dimethylamine (HNMe_2_) produced during the prior transamination step. Additionally, imine formation may also occur through the direct condensation of anilines with aldehyde, presenting an alternative pathway. Subsequently, enaminone is added nucleophilically to imine B-4, producing intermediate B-5. This intermediate undergoes cyclization and tautomerization, leading to the formation of amino tetrahydroquinoline B-7 *via* intermediate B-6. Finally, the elimination of HNMe_2_ from B-8, followed by aromatization, yields the final products of the reaction.

The findings from this methodology reveal a significant advancement in the Povarov reaction. Unlike traditional approaches that utilize terminal alkynes or alkenes to generate C3–C4 fragment sources—resulting in the formation of 2,4-disubstituted quinolines—this innovative method enables the rapid and highly regioselective synthesis of 2,3-disubstituted quinolines. This represents a noteworthy modification of the classic Povarov reaction, highlighting its potential for more versatile applications in organic synthesis.

**Scheme 6:**
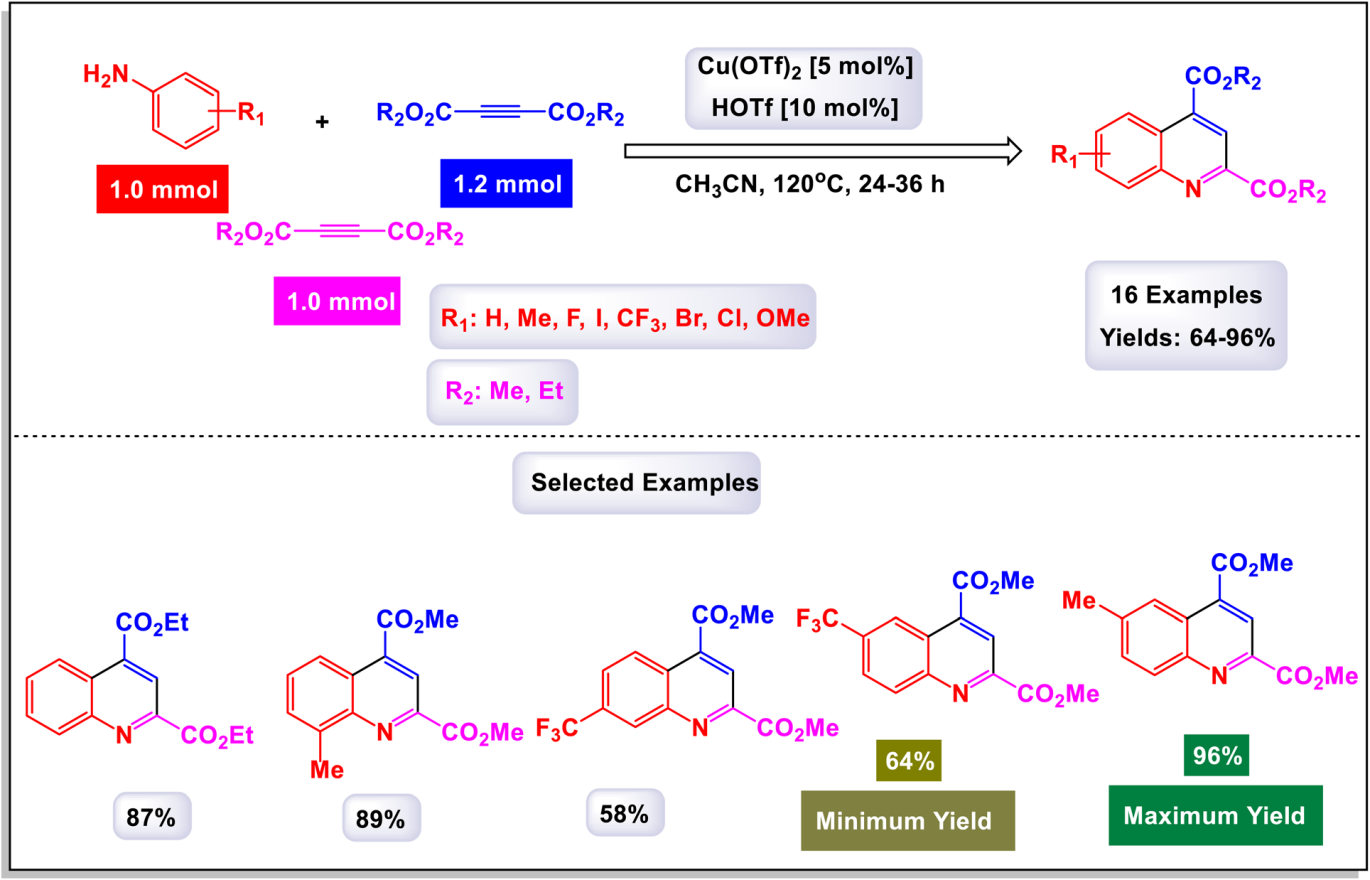
Synthesis of 2,4-disubstituted quinolines from alkynes [catalysis by Cu(OTf)_2_].

**Scheme 7:**
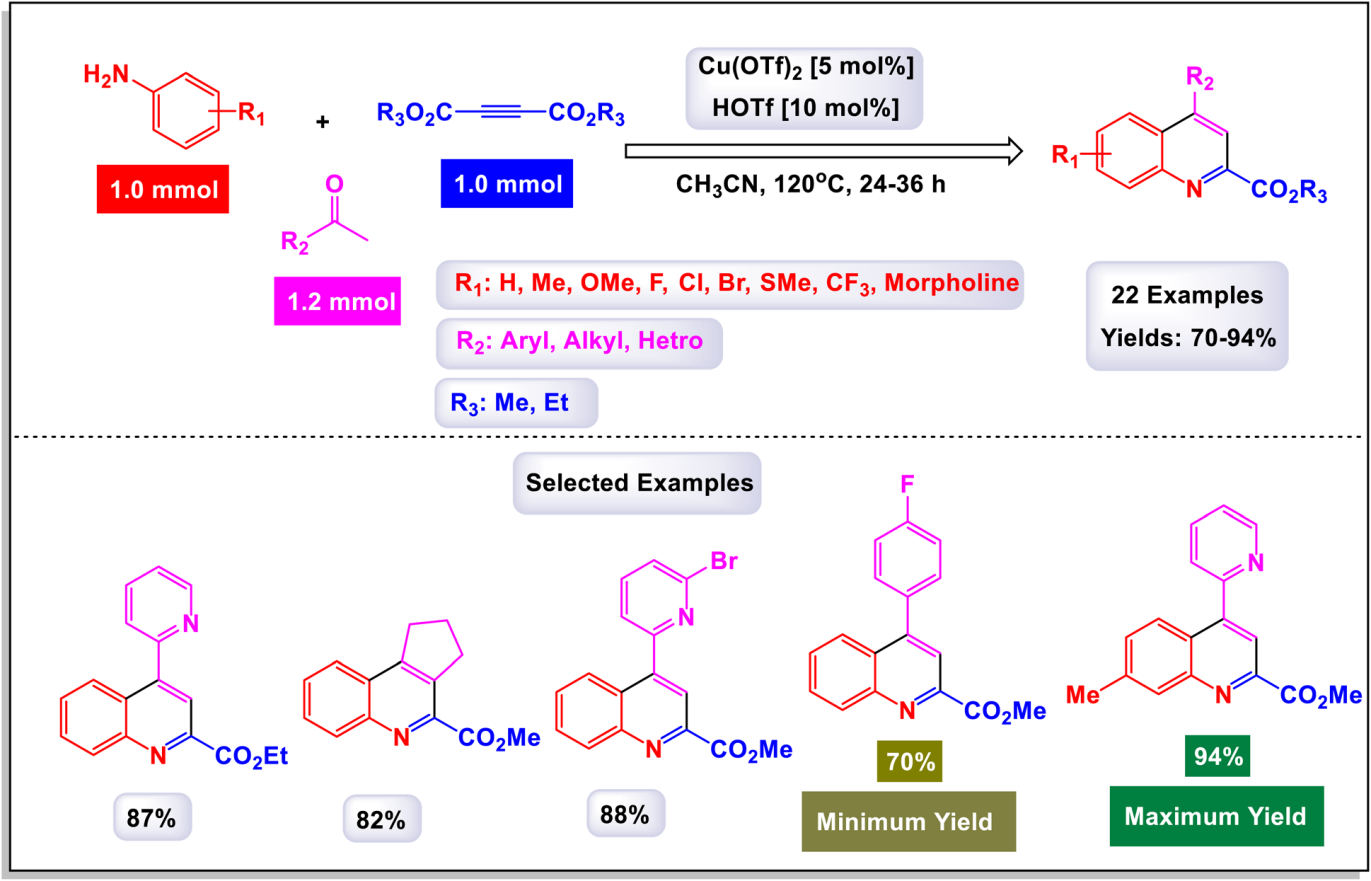
Synthesis of 2,4-disubstituted quinolines from ketones [catalysis by Cu(OTf)_2_].

**Scheme 8:**
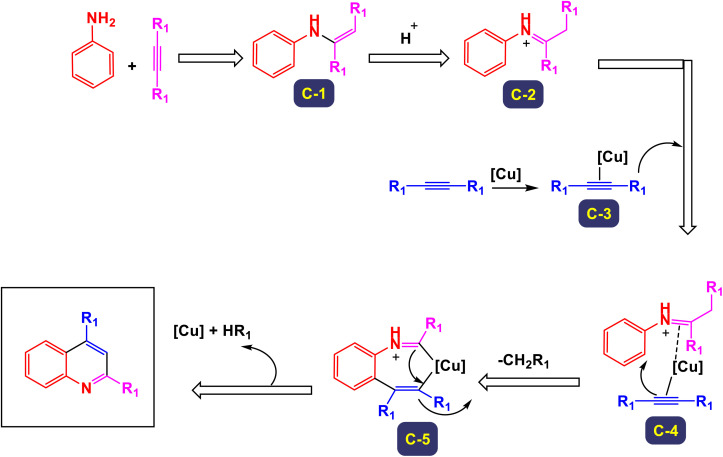
Plausible mechanism for the synthesis of 2,4-disubstituted quinolines from alkynes [catalysis by Cu(OTf)_2_].

**Scheme 9:**
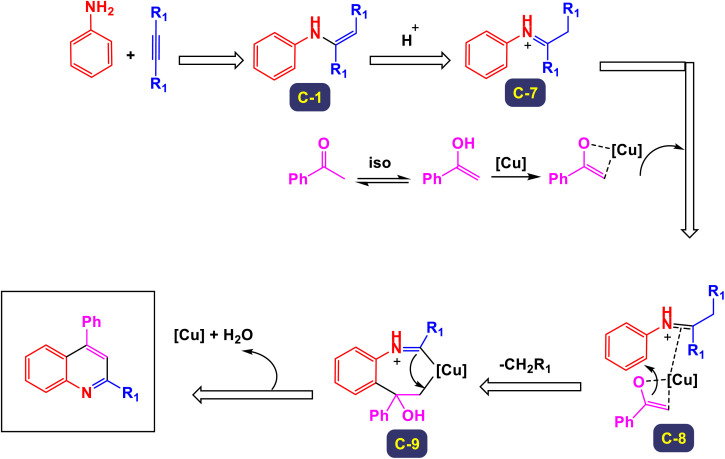
Plausible mechanism for the synthesis of 2,4-disubstituted quinolines from ketones [catalysis by Cu(OTf)_2_].

Zhang *et al.* successfully unveiled a novel copper(ii)-catalyzed cascade annulation process that utilizes simple anilines in conjunction with two molecules of alkyne esters.^74^ This innovative one-pot synthesis method leads to the formation of 2,4-disubstituted quinolines, characterized by their exclusive regioselectivity and impressive tolerance for various substrates and functional groups. As illustrated in Schemes 6 and 7, the versatility of this reaction was further exemplified by the incorporation of a second molecule of alkyne esters, extending the scope to include (hetero)aryl and cycloalkyl–ketone substrates. This extension effectively highlights the robustness and applicability of the present Cu(ii)-catalyzed system, reinforcing its potential in synthetic chemistry. The proposed mechanism in Schemes 8 and 9 outlines the rapid formation of enamine C-1 through the nucleophilic addition of aniline to alkyne ester. This involves Cu(OTf)_2_ forming a copper acetylide species, intermediate C-3, which interacts with imine C(2)-produced from enamine's isomerization and protonation with HOTf—leading to intermediate C-4. The regioselective migratory insertion of alkyne ester forms the intermediate C-5, followed by protonolysis, yielding product 3a, the active Cu(ii) catalyst, and byproducts methanol and carbon dioxide. For ketone substrates, after imine C-2 synthesis, the enol form of the substrate coordinates with Cu(ii) to form intermediate E, which further interacts with imine C-7 to create intermediate C-8. Another regioselective migratory insertion occurs, producing intermediate C-9, and its protonolysis generates the product, along with the active Cu(ii) catalyst and water as a byproduct.

These methods offer a range of notable advantages, including highly specific regioselectivity that directs reactions to desired locations on substrates. They demonstrate impressive tolerance toward various substrates and functional groups, accommodating a wide array of chemical environments. The methods not only yield results that are consistently good to excellent, but they also contribute to the formation of meaningful and complex chemical structures in the resulting products.

**Scheme 10:**
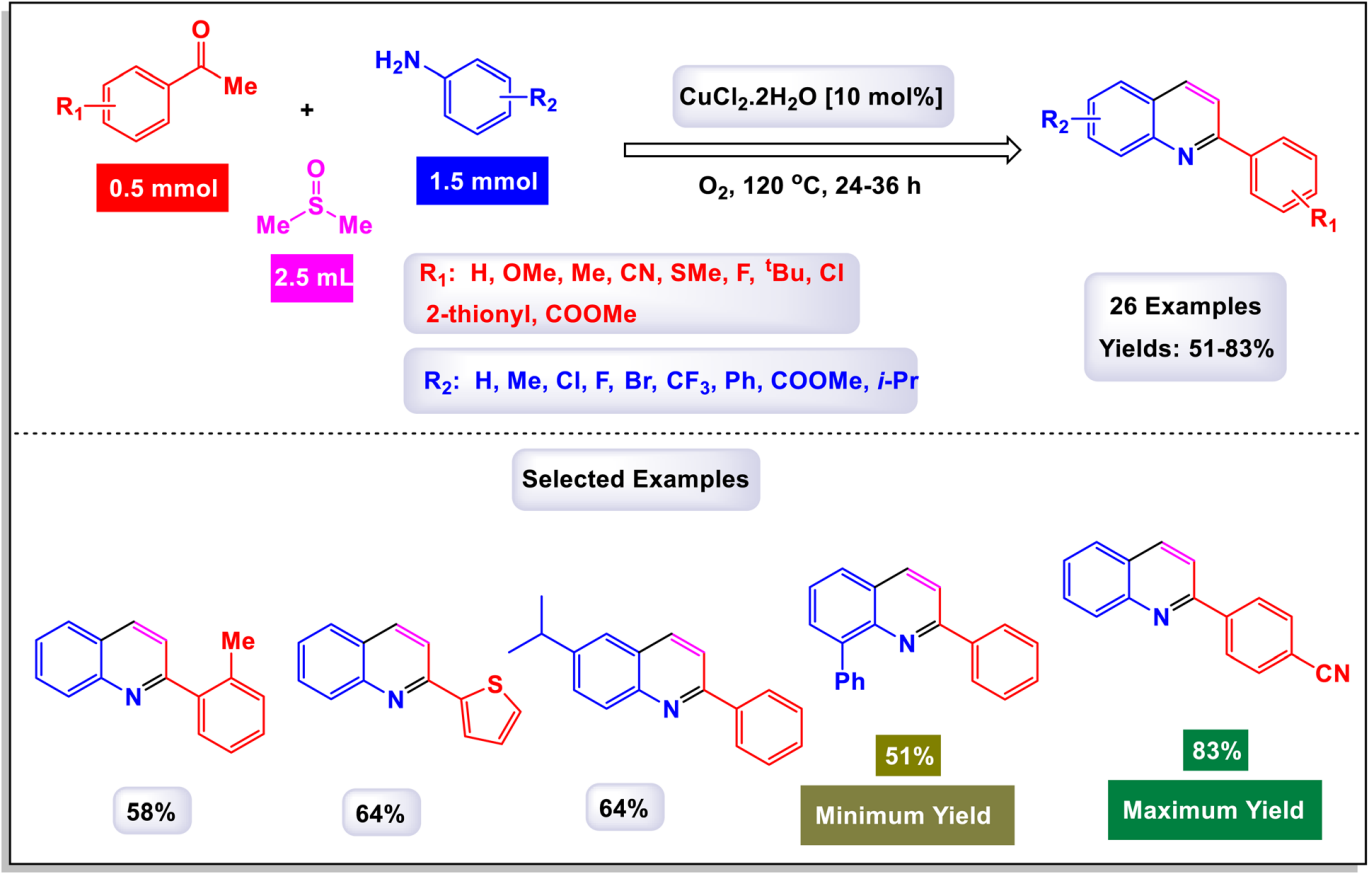
Synthesis of 2-arylquinolines [catalysis by CuCl_2_].

**Scheme 11:**
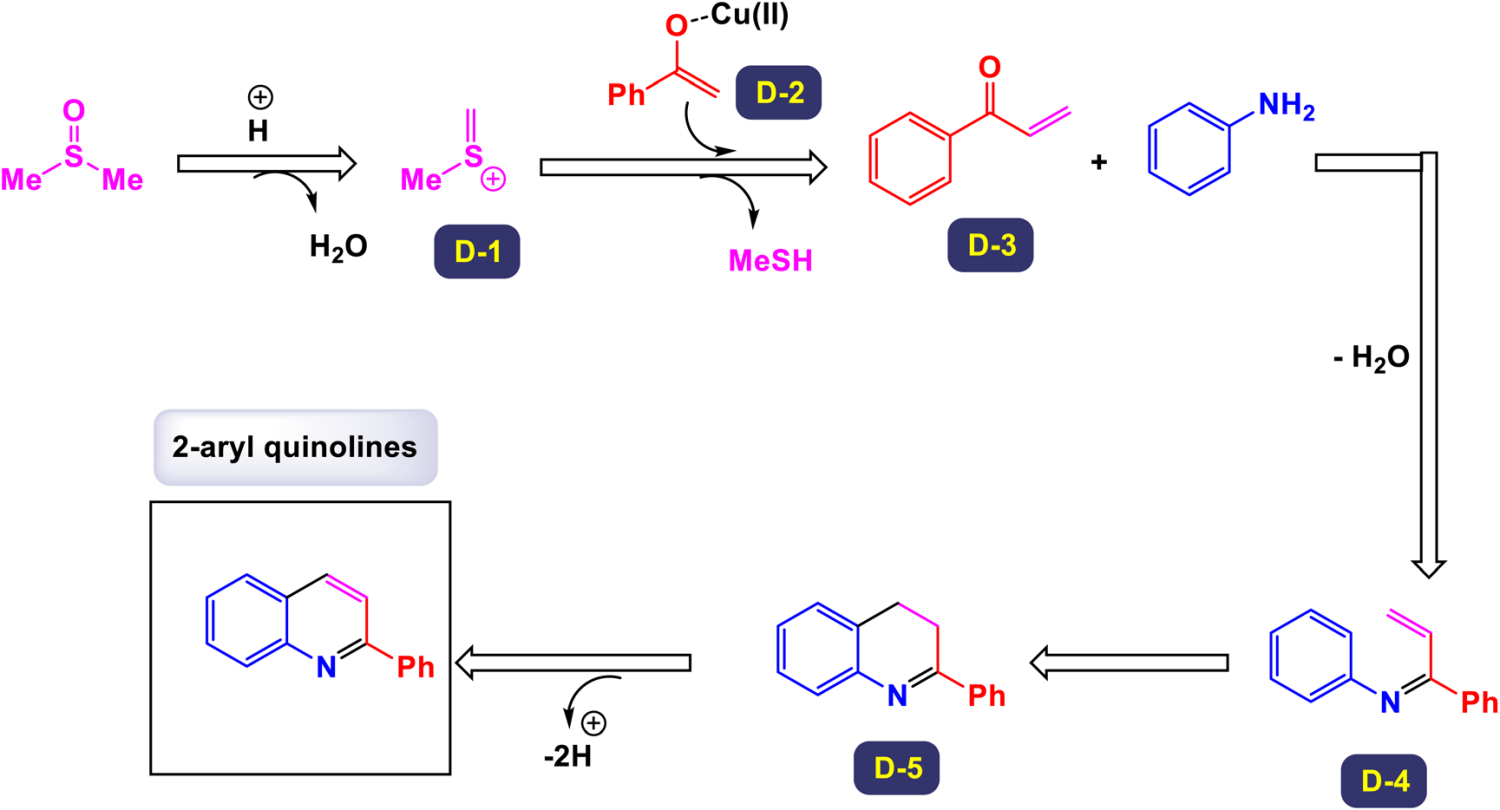
Plausible mechanism for the synthesis of 2-arylquinolines [catalysis by CuCl_2_].

The research team led by Liu explored a novel Cu-catalyzed method for the cyclization of simple anilines in conjunction with ketones, utilizing dimethyl sulfoxide (DMSO) as a one-carbon source.^75^ This innovative approach leverages an aerobic oxidative protocol, allowing for the efficient conversion of various ketones and anilines into 2-arylquinolines, as opposed to the less desired 4-arylquinolines. This methodology not only demonstrates a high level of atom economy but also provides a straightforward strategy to synthesize biologically relevant 2-arylquinolines. As illustrated in Scheme 10, the procedure accommodates arylamines that contain both electron-rich substituents—such as 4-methyl, 4-isopropyl, 2-methyl, 2-phenyl, and 3,5-dimethyl groups—as well as electron-deficient groups including 4-chloro, 4-fluoro, 4-trifluoromethyl, 4-methoxycarbonyl, 3-chloro, and 3-bromo groups. These variations lead to the successful formation of the corresponding products, achieving moderate to good yields across the board. The proposed mechanism (Scheme 11) begins with the activation of DMSO by an aniline, generating an intermediate labeled as D-2. This intermediate can then react with an enolate (designated as A), resulting in a demethylthiolation reaction that produces another species, D-3. Subsequent steps involve the condensation of aniline with species D-3 to form an imine (labeled as D-4). The process concludes with the annulation of D-4 and aromatization of D-5, yielding the desired product. This detailed mechanism highlights the intricate transformations involved in the synthesis of 2-arylquinolines.

**Scheme 12:**
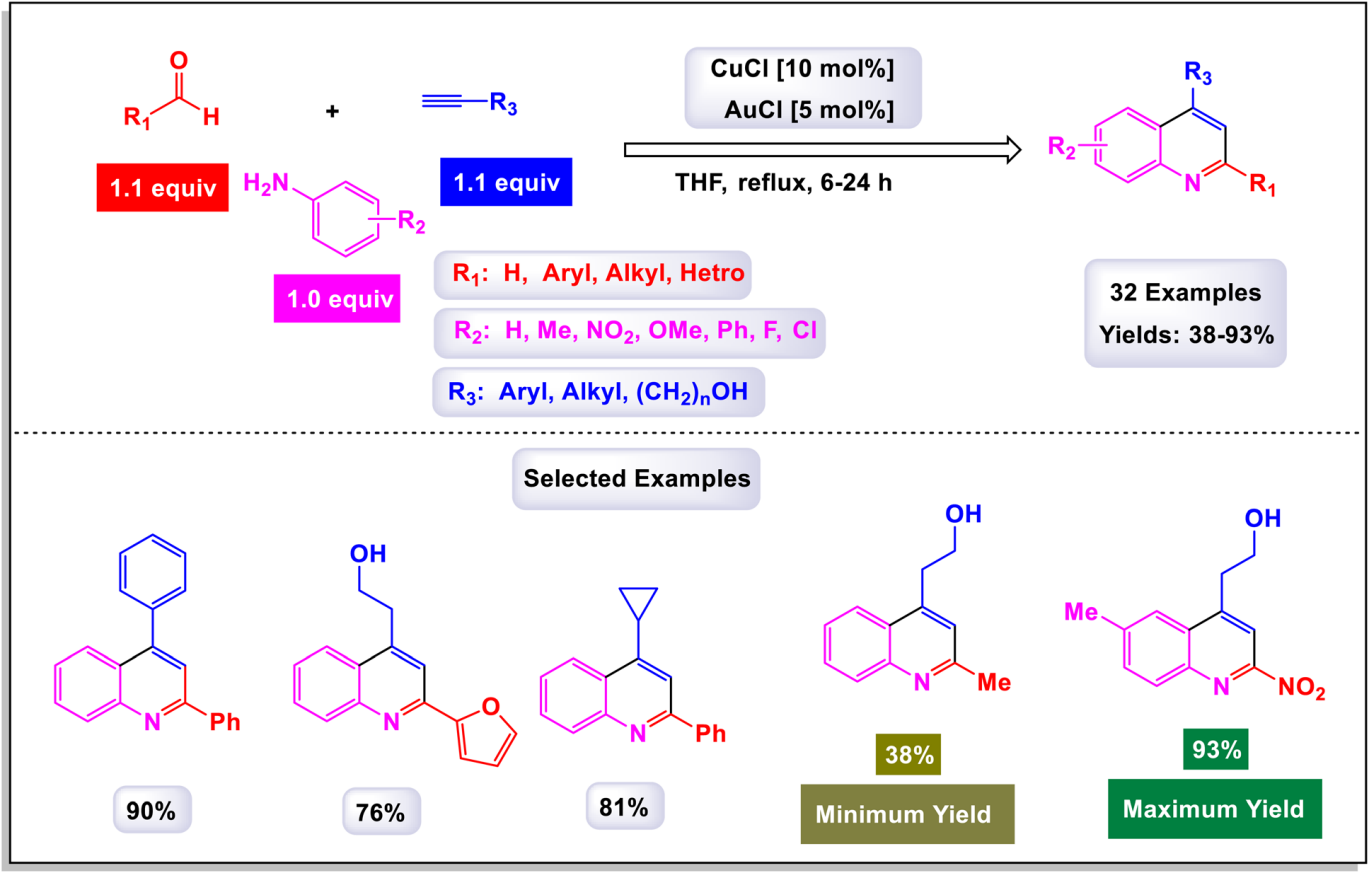
Synthesis of 4-hydroxalkyl-quinolines [catalysis by CuCl/AuCl].

**Scheme 13:**
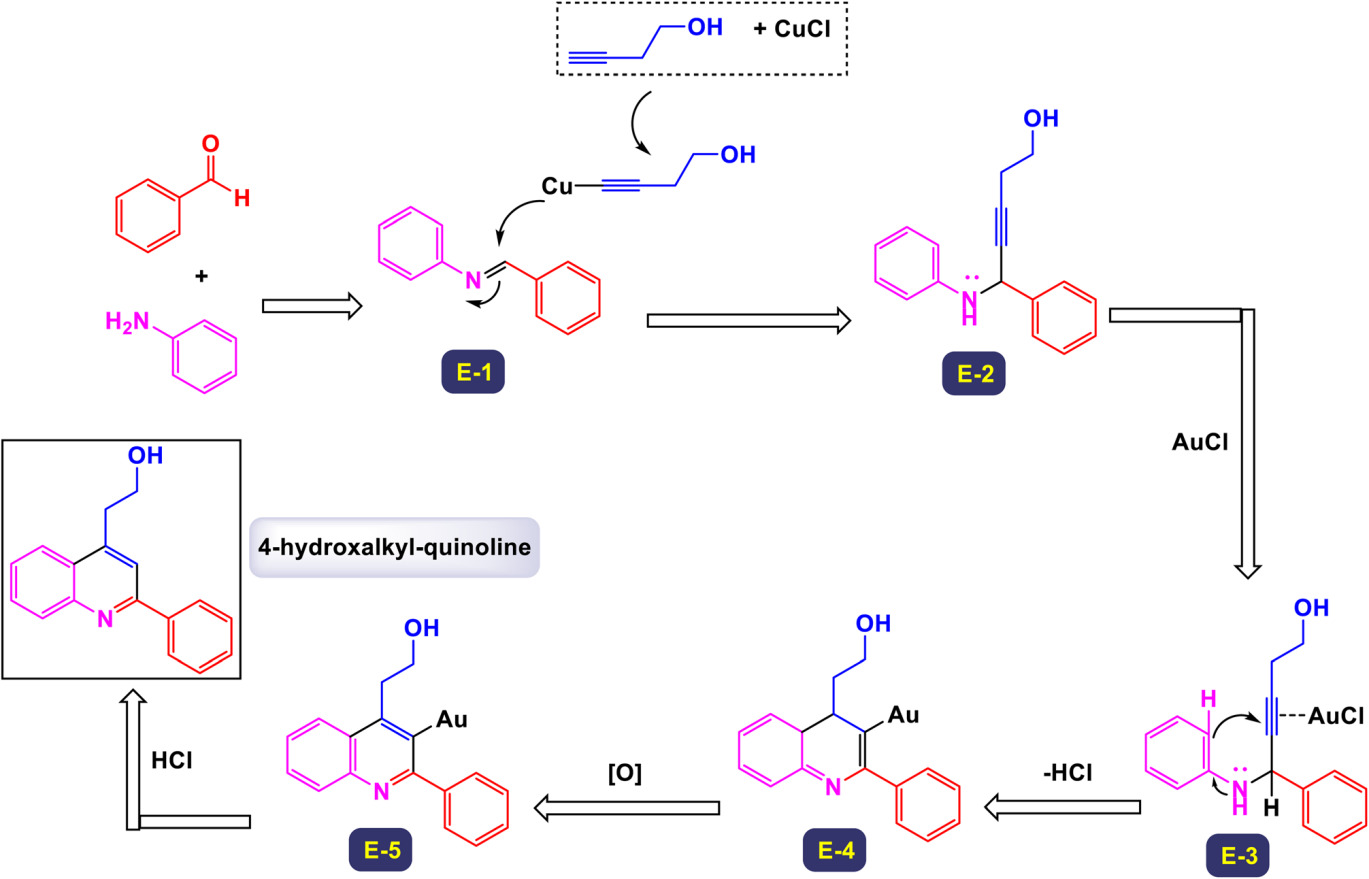
Plausible mechanism for the synthesis of 4-hydroxalkyl-quinolines [catalysis by CuCl/AuCl].

In a groundbreaking study led by the Jin research group, a novel category of 4-hydroxalkyl-quinoline derivatives was synthesized through a sophisticated method employing sequential catalysis with Cu(i) and Au(i).^76^ This innovative approach utilizes a three-component cascade reaction involving anilines, aldehyde derivatives, and aliphatic alkynes, thus facilitating efficient access to a diverse array of quinoline-based compounds. The process is particularly notable for yielding quinolines that feature an aliphatic chain substituent at the 4-position, often achieved with remarkably high yields. The versatility of this reaction is highlighted by the successful application of various substituted aromatic aldehydes and anilines. Notably, substitutions at the para or meta positions—including groups such as fluorine, chlorine, nitro, methoxy, and methyl—have demonstrated promising yields, as detailed in Scheme 12. However, the electronic characteristics of these substituents play a crucial role in influencing the outcomes. Electron-rich substituents generally lead to higher yields, while substrates containing electron-deficient groups tend to produce lower yields, as illustrated by the comparison with 4-methyl-aniline. Delving deeper into the mechanism, as described in Scheme 13, this one-pot A3 reaction encompasses multiple stages, beginning with the formation of an imine (intermediate E-1) through the reaction of an aldehyde with a primary amine. This intermediate then undergoes an imine addition to propargyl amine (E-2), followed by cyclization and oxidation processes. Next, an alkynyl-Cu(i) complex is created, which leads to nucleophilic decomposition that results in the desired quinoline product. The initial activation of the terminal C–H bond on the alkyne is achieved by the Cu(i) catalyst, allowing the formation of the alkynyl-Cu(i) complex (E-3). This complex subsequently engages in nucleophilic addition to the imine produced *in situ*, generating propargyl amine. Furthermore, the triple bond of propargyl amine interacts with AuCl through the principles of the Hard and Soft Acids and Bases (HSAB) theory; here, the soft Lewis base nature of the alkyne is favorably coordinated by the soft Lewis acid Au^+^, which enhances the electrophilicity of the resulting allene. As the reaction progresses, cyclization takes place, prompting an intramolecular nucleophilic attack by the *N*-substituted phenyl ring attached to HCl. This step is followed by an oxidative aromatization with molecular oxygen, resulting in the formation of the quinoline-Au(i) complex, which originates from a dihydroquinoline intermediate (E-4). The final transformation occurs as the quinoline-Au(i) complex (E-5) undergoes protodeauration, catalyzed by HCl, ultimately yielding the target 4-hydroxalkyl-quinoline product. This comprehensive process represents a significant advancement in the synthesis of quinoline derivatives, showcasing the innovative application of metal catalysis in organic chemistry.

**Scheme 14:**
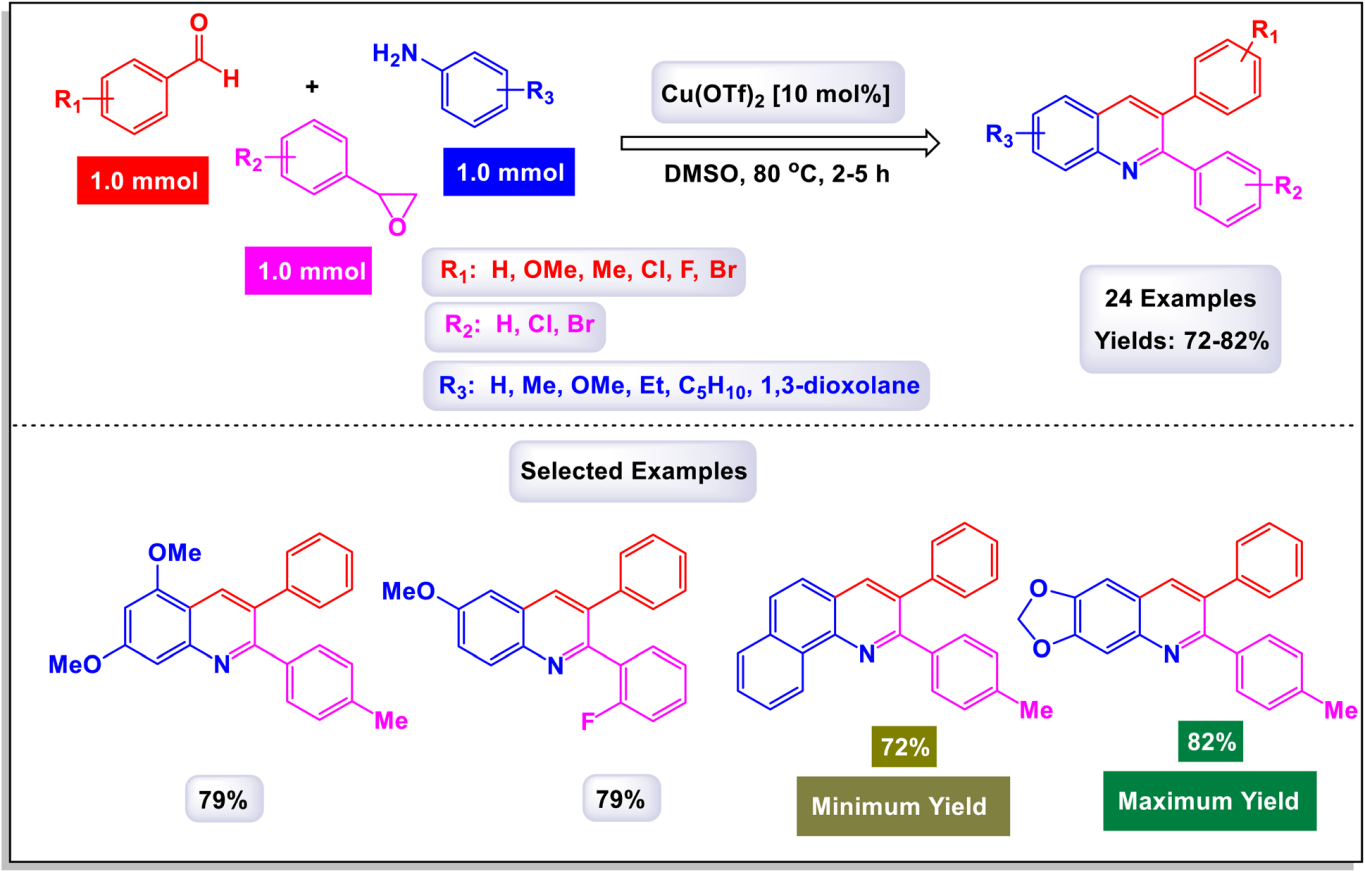
Synthesis of 2,3-diarylquinoline derivatives.

**Scheme 15:**
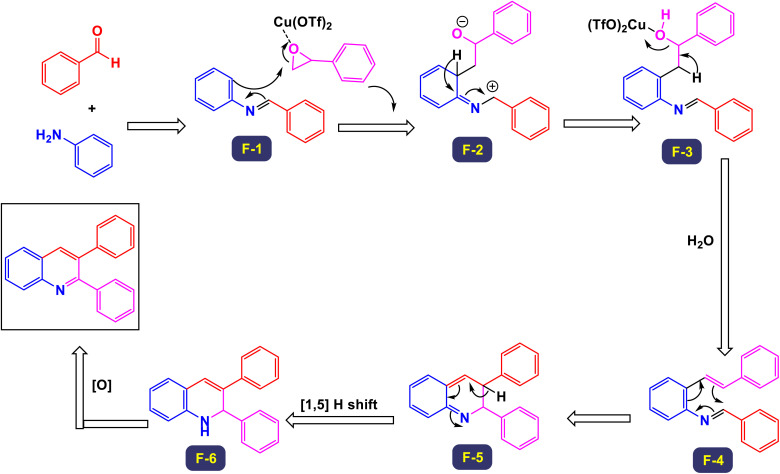
Plausible mechanism for the synthesis of 2,3-diarylquinoline derivatives.

The research group led by Khan has introduced a highly efficient and straightforward synthetic protocol for the production of 2,3-diarylquinoline derivatives.^77^ This method utilizes readily accessible starting materials, namely aryl amines, aryl aldehydes, and styrene oxides. The synthesis is catalyzed by 10 mol% copper(ii) triflate, facilitating a seamless three-component reaction. This innovative approach not only streamlines the synthetic process but also enhances the overall yield of the desired compounds. In Scheme 14, the outlined methodology illustrates the reaction between an imine, which is generated *in situ* from the combination of an aryl amine and an aryl aldehyde, and styrene oxide. This reaction facilitates the synthesis of the target products, showcasing the effectiveness of this approach in achieving the desired chemical transformations. The proposed mechanism for the synthesis of 2,3-diarylquinoline derivatives is illustrated in Scheme 15. Initially, aryl amine engages in a reaction with aryl aldehyde, leading to the formation of an intermediate compound (F-1), which is identified as a Schiff's base. This intermediate then reacts with styrene oxide to yield another intermediate, labeled (F-2). Through a process of aromatization, compound (F-2) transforms into intermediate (F-3), which is detected through mass spectrometry analysis. Subsequently, intermediate (F-3) undergoes a dehydration process, resulting in the elimination of a water molecule to form another intermediate designated as (F-4). This compound (F-4) is further transformed *via* a 6π-electrocyclic ring closure, creating intermediate (F-5). Notably, intermediate (F-5) is characterized by a^1,5^ hydrogen shift that produces dihydroquinoline (F-6). Following this, an aerial oxidation process occurs, ultimately yielding the target quinoline compound. This methodology stands out due to its cost-effectiveness and high atom economy, which reduces waste and makes it accessible for various applications. It offers exceptional regioselectivity, ensuring targeted reactions, and is user-friendly with shorter reaction times that enhance efficiency. The technique allows for the consecutive formation of one carbon-nitrogen (C–N) bond and two carbon–carbon (C–C) bonds, enabling the construction of complex molecular structures. Additionally, its broad substrate scope consistently yields good results, making it a valuable tool for researchers.

**Scheme 16:**
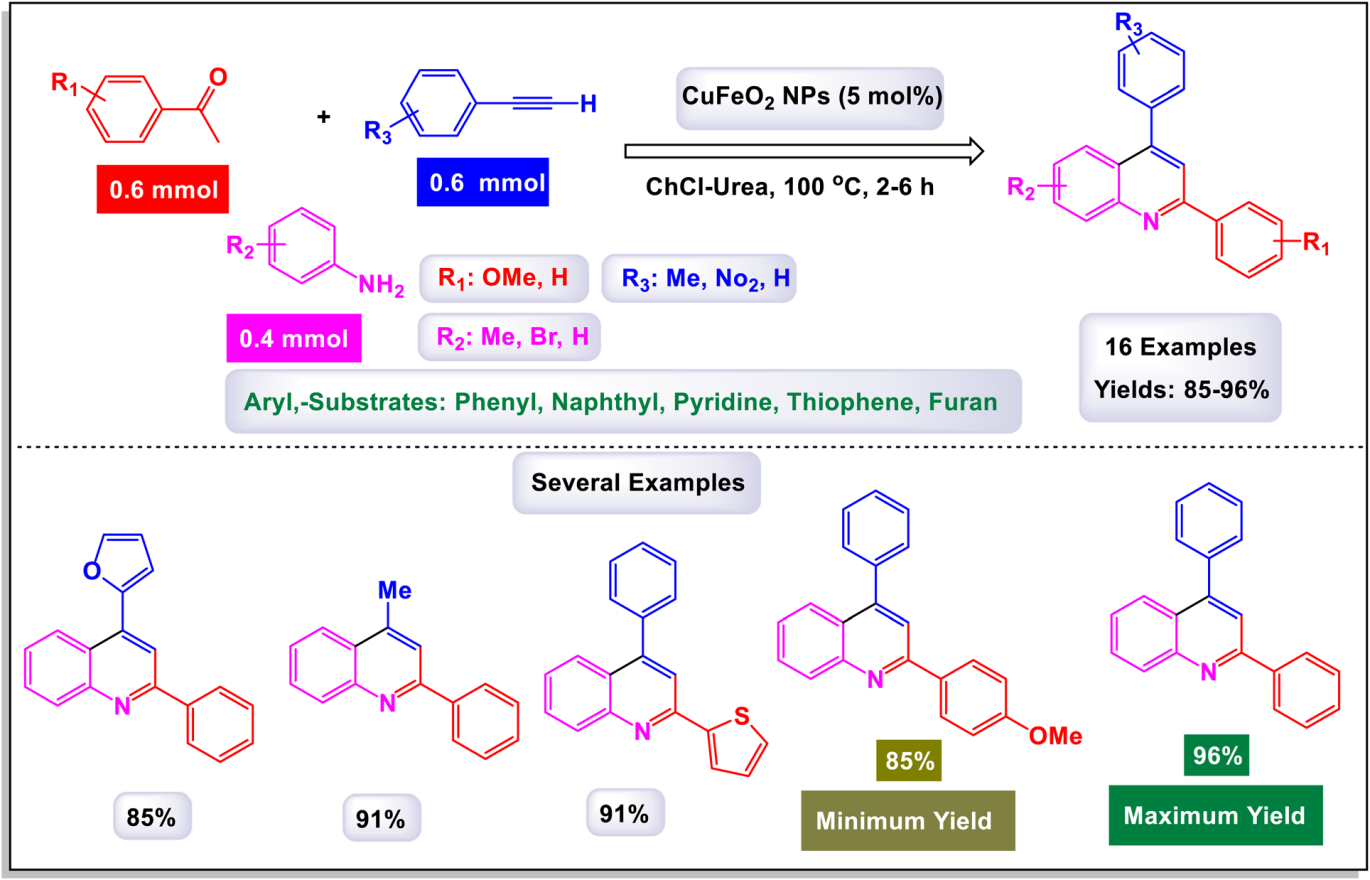
Synthesis of 2,4-substituted quinolines [catalysis by CuFeO_2_ nanoparticles].

**Scheme 17:**
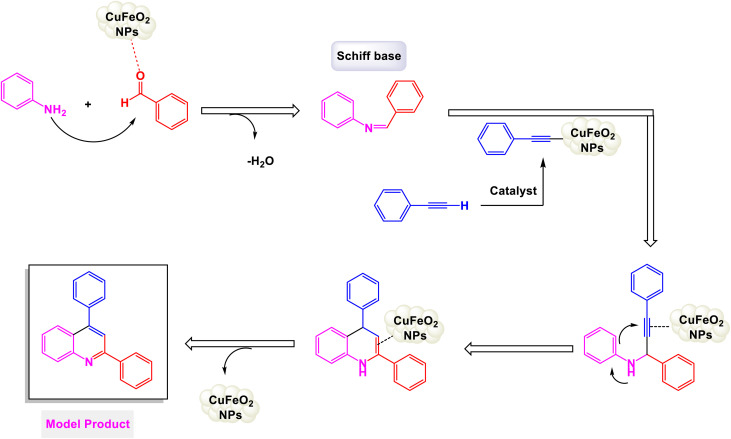
Plausible mechanism for the synthesis of 2,4-substituted quinolines [catalysis by CuFeO_2_ nanoparticles].

In a noteworthy development, Kazemi *et al.* unveiled a captivating technique for synthesizing 2,4-substituted quinolines, employing CuFeO_2_ nanoparticles within a ChCl-Urea green catalytic system.^78^ As illustrated in Scheme 16, their study explored an array of aromatic aldehyde derivatives, each adorned with diverse functional groups on the aromatic rings, revealing the versatility of this method. Astonishingly, regardless of the assortment of functional groups present, the reaction yielded products with impressively high efficiency. This finding underscores the robust nature of the catalytic system, demonstrating that the variations in functional groups have a minimal impact on the overall efficacy. Further expanding their investigation, the effectiveness of several heteroaryl aldehydes as substrates was scrutinized. The results exceeded expectations, showcasing that each type of heteroaryl aldehyde produced high yields, reinforcing the catalyst's remarkable performance. According to the mechanism detailed by this research team in Scheme 17, the process begins with the activation of the substrate by the catalyst, paving the way for cyclization. This is followed by a nucleophilic attack at the ortho position of aniline on the resulting intermediate, leading to the formation of a cyclic intermediate. The culmination of this intricate sequence is an intramolecular rearrangement that produces the sought-after 2,4-diphenylquinoline.

Remarkably, the CuFeO_2_ catalyst demonstrated outstanding reusability, retaining its catalytic prowess even after being recycled up to nine times, a testament to its exceptional stability and efficiency.

### 2.2 Catalysis by iron

Among the myriad of catalysts that have been employed in chemical reactions, iron has emerged as a significant player, particularly in the context of heterocycle synthesis. Iron, being one of the most abundant transition metals in the Earth's crust, is readily available and cost-effective compared to noble metals such as palladium or platinum.^79^ This economic aspect is particularly advantageous for large-scale industrial applications. Furthermore, the natural abundance of iron implies that the environmental impact associated with mining and refining precious metals is significantly mitigated when iron is used, aligning with the principles of green chemistry.^80^

#### 2.2.1 Advantages of iron as a catalyst in heterocycle synthesis

✓ *Ability to promote diverse reactions*: Iron catalysts demonstrate broad applicability across numerous reaction types essential for heterocycle synthesis, including cross-coupling reactions, C–H activation, and electrophilic aromatic substitutions.^81^ By facilitating these reactions, iron enables the formation of a wide variety of heterocyclic compounds, which are crucial in pharmaceuticals, agrochemicals, and biologically active molecules.

✓ *Promoting reactivity and selectivity*: The catalytic properties of iron can often enhance reactivity while allowing for greater control over selectivity. This is a crucial consideration in heterocycle synthesis, where the goal is often to obtain specific products with minimal by-products. Iron catalysts can operate under milder conditions than those required for many traditional catalysts, subsequently minimizing side reactions that result in undesired products.^82^

✓ *Environmental sustainability*: The use of iron as a catalyst contributes significantly to the sustainability of chemical processes. Iron catalysts are less toxic than their precious metal counterparts, and their incorporation into synthetic pathways often results in less hazardous waste and reduced reliance on environmentally damaging reagents. Moreover, when iron is used in catalysis, the reaction can often proceed in aqueous media, providing a greener alternative to organic solvents.^83^

✓ *Innovation through iron-complex catalysis*: Advances in coordination chemistry have led to the development of various iron complexes that exhibit unique catalytic properties. These complexes can be tailored to suit specific substrates or reaction conditions, further enhancing their utility in synthesizing complex heterocycles. For example, specific ligand design can influence the electronic properties of the iron center, thus tuning the catalyst's activity and selectivity.^83^

✓ *Compatibility with functional groups*: Many iron-catalyzed reactions exhibit compatibility with a wide range of functional groups, making them particularly useful in synthesizing complex heterocycles that possess multiple reactive sites. This functional group tolerance enables chemists to navigate synthetically challenging landscapes and construct intricate molecular architectures that would otherwise be difficult to achieve.^84^

✓ *Facilitation of alternative reaction pathways*: Iron catalysts are often able to activate unique reaction pathways that may be inaccessible with other metal systems. For instance, they can induce C–H activation processes under mild conditions, allowing for the functionalization of unactivated C–H bonds in heterocycles.^74^ Such transformations potentially expand the toolbox available for heterocycle synthesis and allow for more inventive approaches to molecular construction.^85^

The role of iron as a catalyst in chemical reactions, particularly in the synthesis of heterocycles, underpins its importance in contemporary synthetic chemistry. Its abundance, economic viability, low toxicity, and efficiency in promoting diverse chemical transformations position iron as an exemplary catalyst for both research and industrial applications. By harmonizing the principles of reactivity, selectivity, and sustainability, iron empowers chemists to venture into increasingly complex territories of molecular construction. As research into iron catalysis continues to evolve, it is anticipated that its significance in the synthesis of heterocycles and broader categories of chemical reactions will only deepen, paving the way for innovative solutions to modern chemical challenges.^86^

**Scheme 18:**
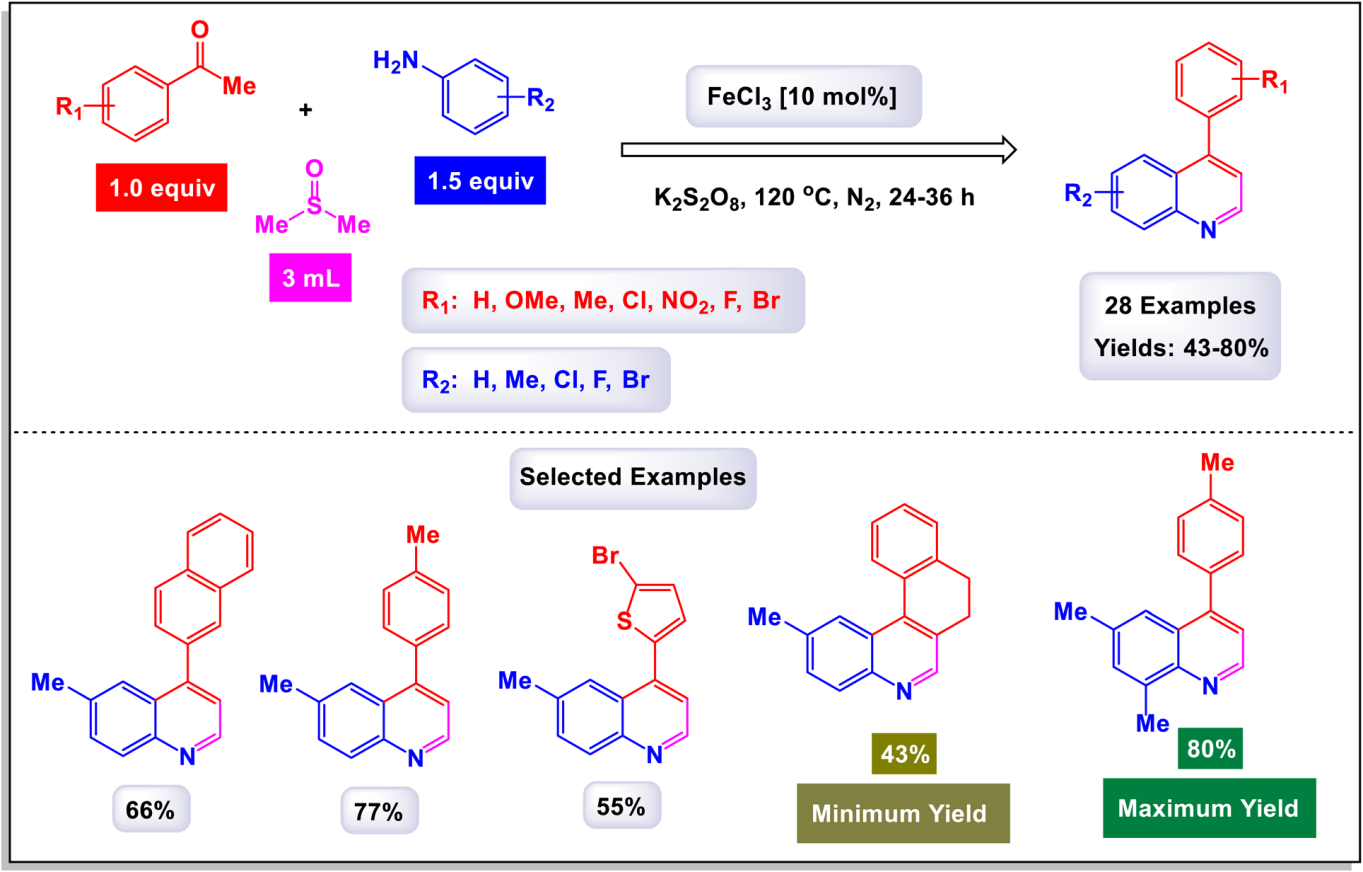
Synthesis of 4-aryl quinolines [catalysis by the FeCl_3_/K_2_S_2_O_8_ system].

**Scheme 19:**
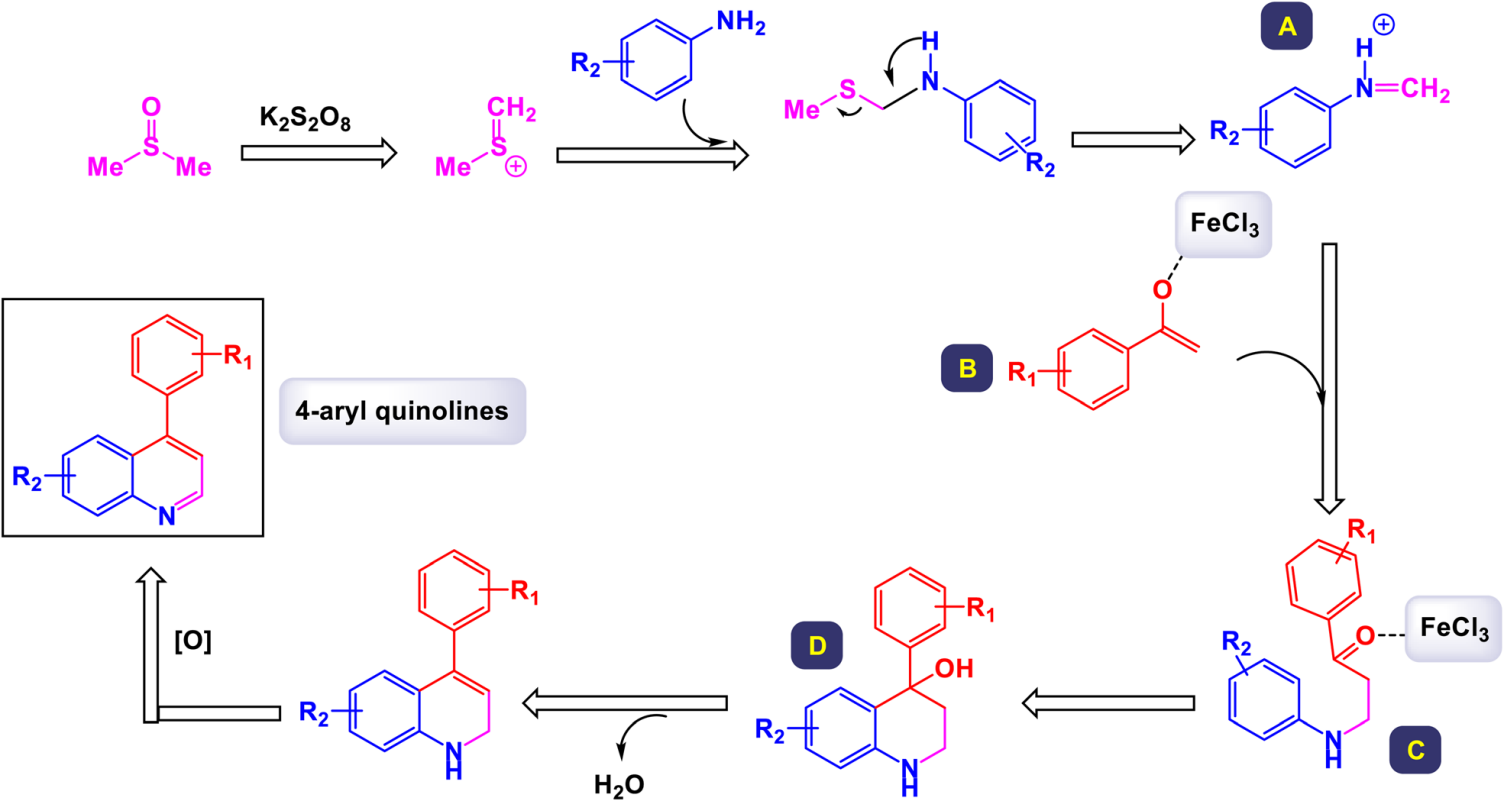
Plausible mechanism for the synthesis of 4-aryl quinolines [catalysis by the FeCl_3_/K_2_S_2_O_8_ system].

In a compelling report, Singh and colleagues introduced a comprehensive and effective methodology for the synthesis of 4-aryl quinolines.^17^ This innovative approach employs one-pot, three-component reactions involving anilines, acetophenone derivatives, and dimethyl sulfoxide (DMSO) as a dual-purpose agent, while catalyzed by 10 mol% of FeCl_3_ in the presence of potassium persulfate (K_2_S_2_O_8_). In this methodology, DMSO not only serves as the solvent but also acts as a methine (=CH–) equivalent, contributing significantly to the reaction's success. As outlined in Scheme 18, a total of 28 distinct examples of 4-aryl quinolines were synthesized, achieving moderate to high yields ranging from 43% to 80%. The process unfolds through a cascade mechanism that includes the generation of a sulfenium ion, followed by the formation of C–N and C–C bonds, culminating in cyclization. The reaction demonstrated impressive versatility, accommodating a wide array of substituents on the starting materials, including both electron-donating and electron-withdrawing groups, as well as halides. Notably, when electron-rich acetophenones were reacted with anilines and DMSO, the synthesis yielded quinolines in good amounts. Conversely, electron-poor acetophenones, such as the 4-nitro derivatives, resulted in moderate to good yields of the respective quinolines. The findings underscored that derivatives with substituents at the *para*-position were particularly well-suited for this reaction. However, it was observed that *para*-unsubstituted anilines were susceptible to oxidative modification and degradation under the reaction conditions, although no such compounds were isolated for analysis. According to the proposed mechanism depicted in Scheme 19, DMSO plays a critical role as a source of methine, reacting with K_2_S_2_O_8_ in the presence of aniline to generate an intermediate structure labeled as G-1. This iminium intermediate subsequently interacts with enolate G-2 to form the compound G-3, which then undergoes cyclization through the formation of a C–N bond. The final steps involve dehydration followed by oxidation of the intermediate G-4, leading to the formation of the desired quinoline products.

**Scheme 20:**
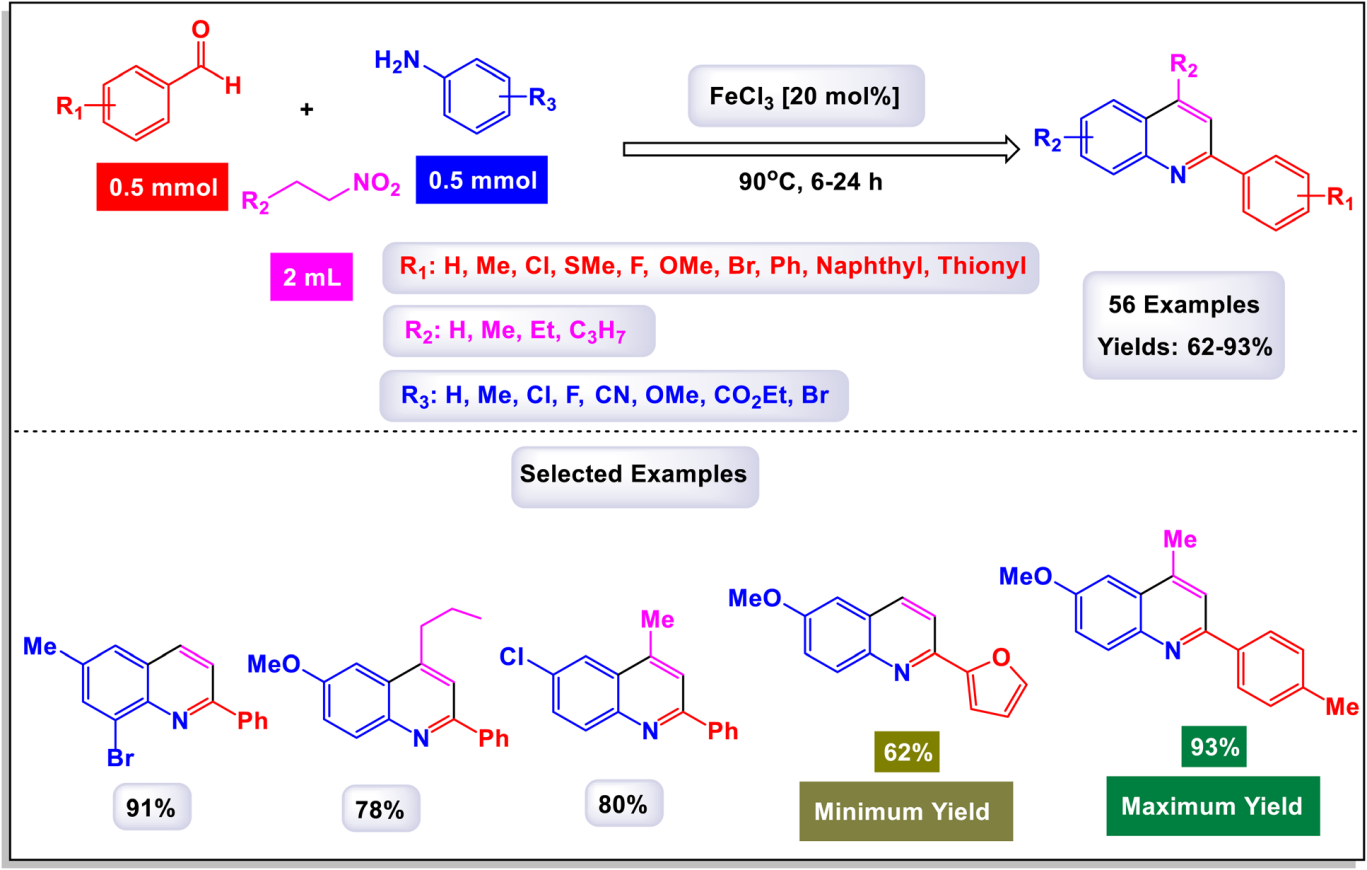
Synthesis of 2-aryl quinolines [catalysis by FeCl_3_].

**Scheme 21:**
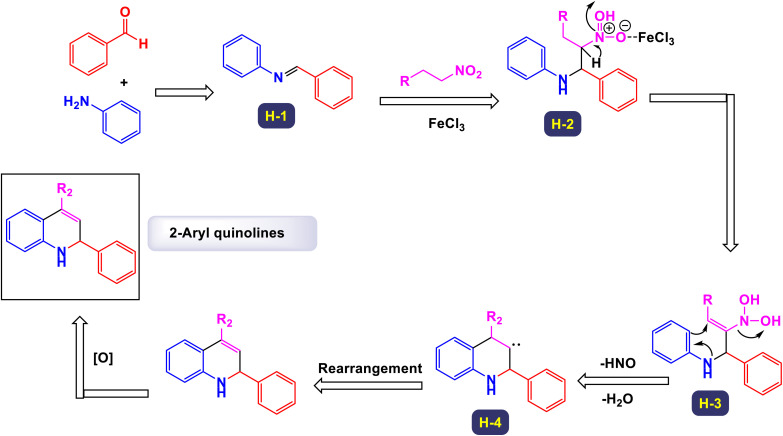
Plausible mechanism for the synthesis of 2-aryl quinolines [catalysis by FeCl_3_].

In a noteworthy publication, the research group led by Majee introduced an innovative one-pot method for synthesizing 2-aryl quinolines utilizing iron(iii) chloride (FeCl_3_) as a catalyst under ambient conditions.^87^ To establish standardized reaction conditions, they conducted extensive testing of a model reaction, evaluating a variety of catalysts, including iron, copper, aluminum, and zinc salts. Through a systematic series of experiments, the authors demonstrated that this synthesis approach is not only efficient at the laboratory scale but also highly effective for gram-scale production, with minimal loss of efficiency. This finding highlights the method's potential for large-scale applications in the synthesis of 2-aryl quinoline derivatives. The results indicated that incorporating both electron-withdrawing and electron-donating substituents within the same aniline moiety leads to the successful formation of the desired quinoline derivatives in excellent yields, as illustrated in Scheme 20. The current reaction likely proceeds through a series of interconnected processes, specifically a sequential aza-Henry reaction followed by cyclization and denitration, as illustrated in Scheme 21. Observations from the reaction suggest that it begins with the formation of an imine intermediate (designated as H-1). This imine then reacts with a nitroalkane, resulting in the generation of the aza-Henry adduct (labeled H-2). In this aza-Henry intermediate (H-2), a rearrangement of the nitro group occurs, leading to the formation of another intermediate (H-3) that features a geminal hydroxyl group on the nitrogen atom. This transformation is accompanied by the elimination of nitrogen monoxide (HNO) and water (H_2_O). Subsequently, intermediate (H-3) undergoes an ortho cyclization reaction, resulting in the generation of a new carbon–carbon bond alongside the creation of an adjacent carbene (denoted as H-4). Ultimately, the dihydroquinoline structure formed from this carbene undergoes a rearrangement; upon oxidation, it is transformed into the corresponding quinoline compound. This protocol offers several key advantages, including the use of easily accessible and cost-effective commercially available chemicals as starting materials. It utilizes inexpensive metal catalysts and operates under aerobic conditions, which simplifies the process and enhances sustainability. Additionally, the method is tolerant of a wide range of functional groups, allowing for versatility in substrates, while its operational simplicity makes it suitable for both experienced chemists and beginners.

**Scheme 22:**
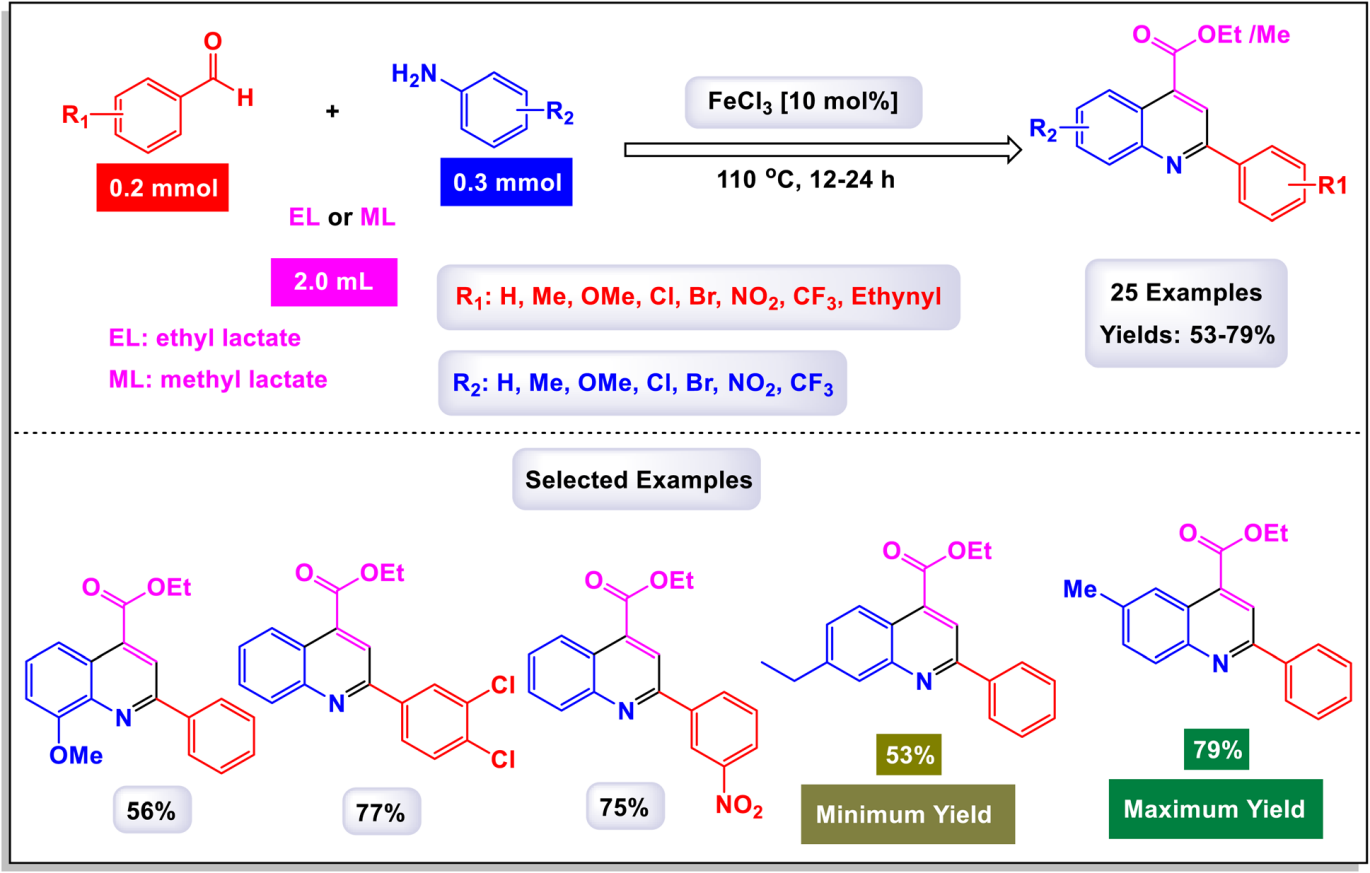
Synthesis of 2,4-disubstituted quinoline derivatives [catalysis by FeCl_3_].

Wan and Yang have introduced an innovative and practical methodology for the synthesis of 2,4-disubstituted quinoline derivatives through a one-pot three-component reaction.^88^ This process involves ethyl or methyl lactate, anilines, and aldehydes, utilizing FeCl_3_ as a catalyst under solvent-free conditions (illustrated in Scheme 22). This three-component approach showcases exceptional efficiency and a wide substrate tolerance, enabling rapid access to a diverse array of quinoline products in a neat reaction environment. The findings demonstrate that anilines with both electron-donating and electron-withdrawing substituents, positioned at the *para*-, *meta*-, and *ortho*-locations, are well accommodated in this reaction. As a result, the desired quinoline derivatives were synthesized with remarkably high yields. Additionally, the methodology successfully incorporates benzaldehydes bearing various substituents, including alkyl, alkoxyl, halogen, trifluoromethyl, and nitro groups, leading to the corresponding quinolines produced in good to excellent yields. Notably, the reaction can tolerate strong electron-withdrawing groups, such as nitro and trifluoromethyl functionalized substrates, which also effectively yielded quinoline products. This highlights the robustness of the method. Furthermore, this synthetic approach is characterized by its high sustainability and greenness, emphasizing advantages such as the utilization of biomass, alcohol dehydrogenation, and a focus on diversity-oriented synthesis. The overall impact of this work contributes significantly to the field of organic synthesis, providing an efficient route to valuable quinoline compounds.

**Scheme 23:**
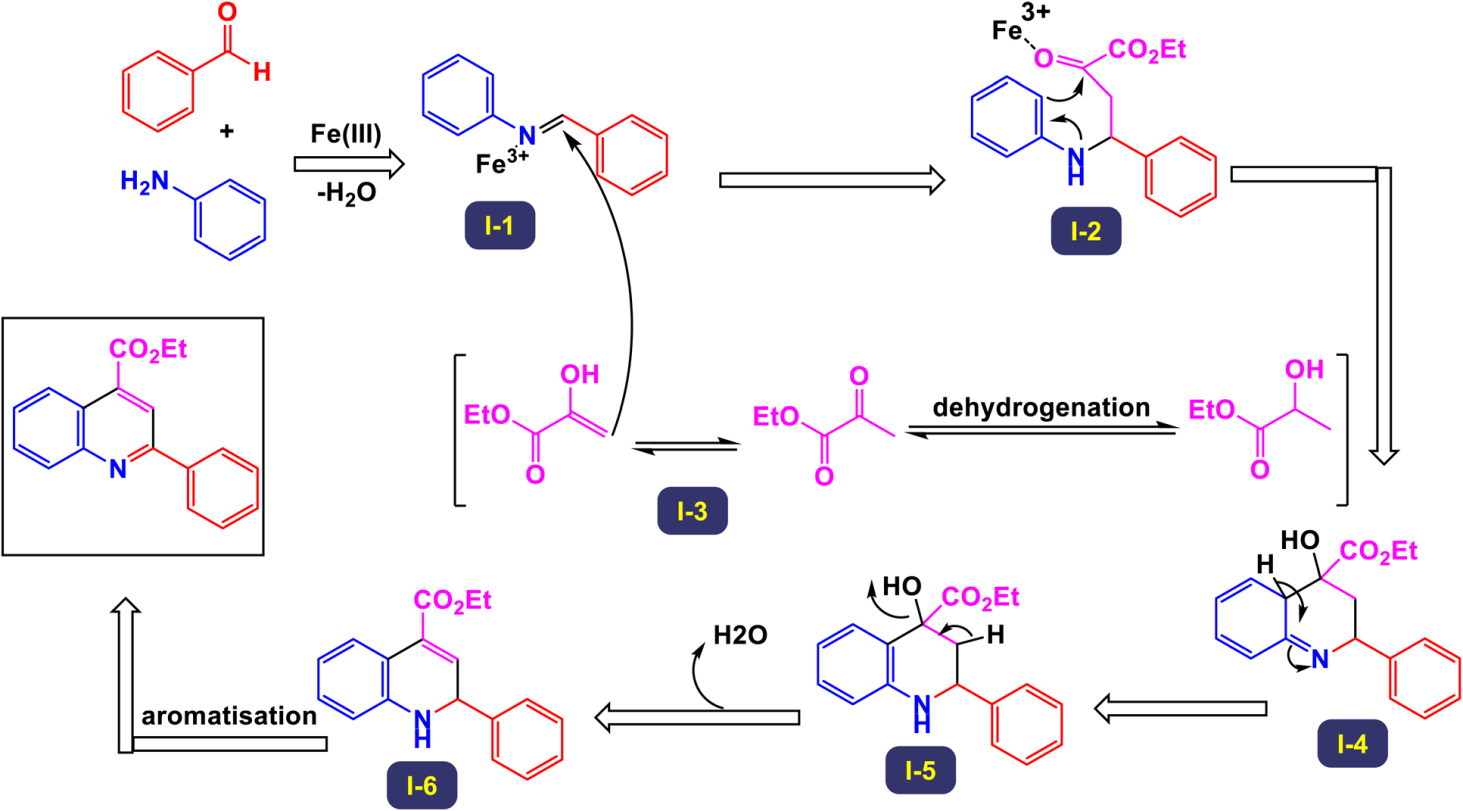
Plausible mechanism for the synthesis of 2,4-disubstituted quinoline derivatives [catalysis by FeCl_3_].

The primary transformation, as illustrated in Scheme 23, involves the dehydrogenation of ethyl lactate (EL) to form ethyl pyruvate (compound I-3). Although this conversion is reversible, ethyl pyruvate can be effectively captured by the imine (compound I-1) that is generated *in situ*. This reaction proceeds through an isomeric intermediate (compound I-3′), allowing for the formation of adduct I-2. Once formed, this adduct undergoes an intriguing intramolecular process where a nucleophilic aryl C–H bond attacks the carbonyl group of the ketone, facilitating a transformation that leads to the elimination of an intermediate (compound I-4). This ultimately gives rise to dihydroquinoline intermediate I-5. Following this step, a further aromatization of compound I-6 occurs, culminating in the production of the desired target compounds.

### 2.3 Catalysis by iridium

Iridium is a highly valuable catalyst in chemical reactions due to its exceptional stability, efficiency, and versatility. It plays a crucial role in various industrial and research applications, particularly in hydrogenation, oxidation, and energy-related processes.^89–92^ The importance of iridium as a catalyst lies in its ability to enhance reaction efficiency, stability, and selectivity while contributing to sustainable chemical processes. Its advantages make it indispensable in industrial applications, clean energy technologies, and advanced chemical transformations.^93–96^

#### 2.3.1 Advantages of catalysis by iridium

✓ *High efficiency & turnover*: Iridium catalysts exhibit high turnover frequencies (TOF) and turnover numbers (TON), meaning they can catalyze many reactions before deactivation. They enable reactions to proceed at lower temperatures and pressures, saving energy.^97^

✓ *Exceptional stability*: Iridium is resistant to oxidation, corrosion, and deactivation, making it durable under harsh reaction conditions. It can function effectively in acidic, basic, and high-temperature environments.^98^

✓ *Versatility in catalysis*: Iridium catalysts are widely used for hydrogenation, oxidation, water splitting, and carbon–carbon bond formation. They facilitate asymmetric synthesis, essential for pharmaceuticals and complex organic molecules.^99^

✓ *Enhanced selectivity*: These catalysts allow for precise control over reaction pathways, leading to higher yields and purity of the desired product. This is crucial for industries requiring high product specificity, such as pharmaceuticals and agrochemicals.^100^

✓ *Role in renewable energy technologies*: Iridium-based catalysts are used in proton-exchange membrane (PEM) electrolyzers for hydrogen generation. They are essential in fuel cells for clean energy applications.^101^

**Scheme 24:**
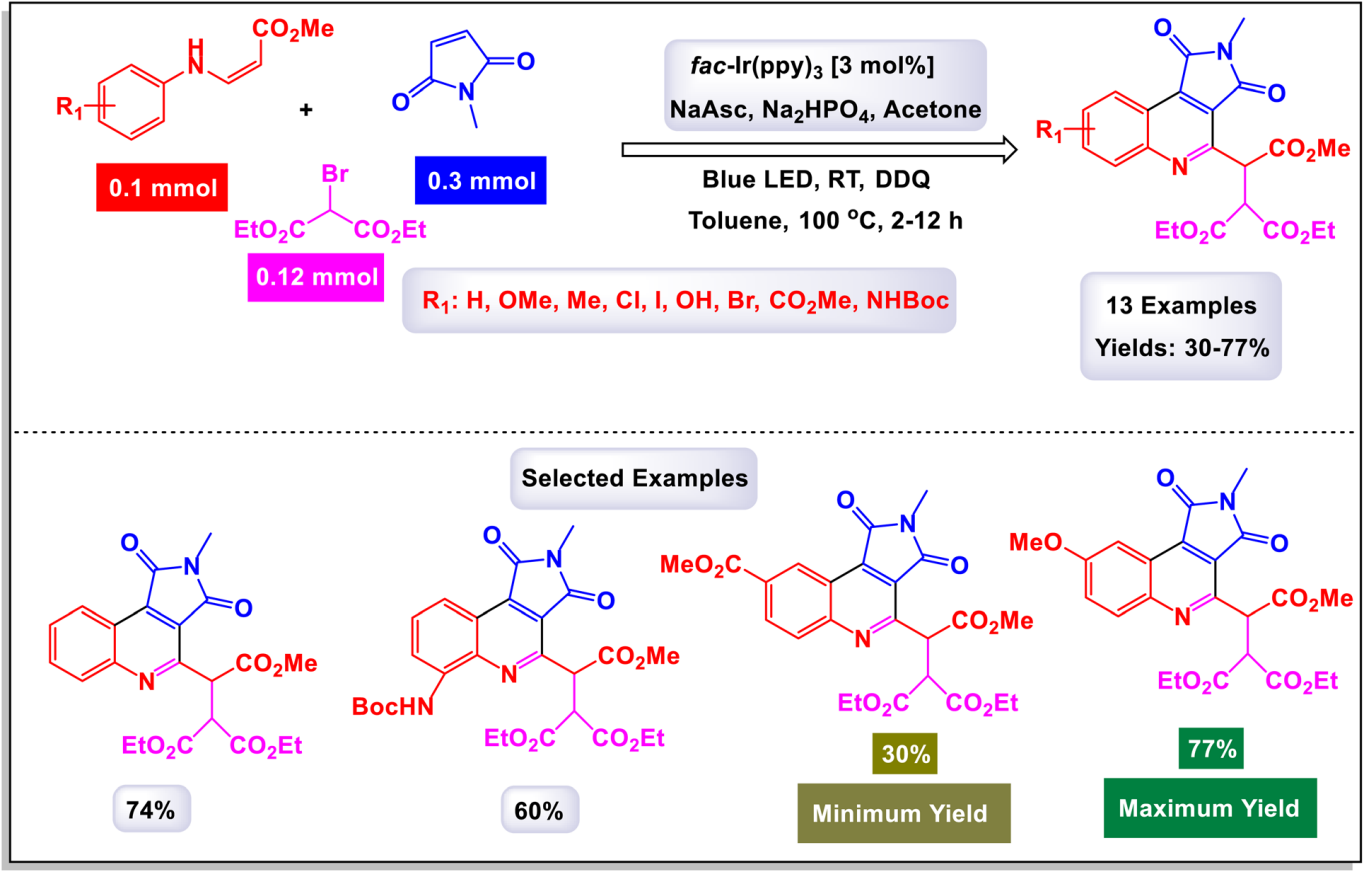
Synthesis of highly substituted quinolines [catalysis by Ir(ppy)_3_].

**Scheme 25:**
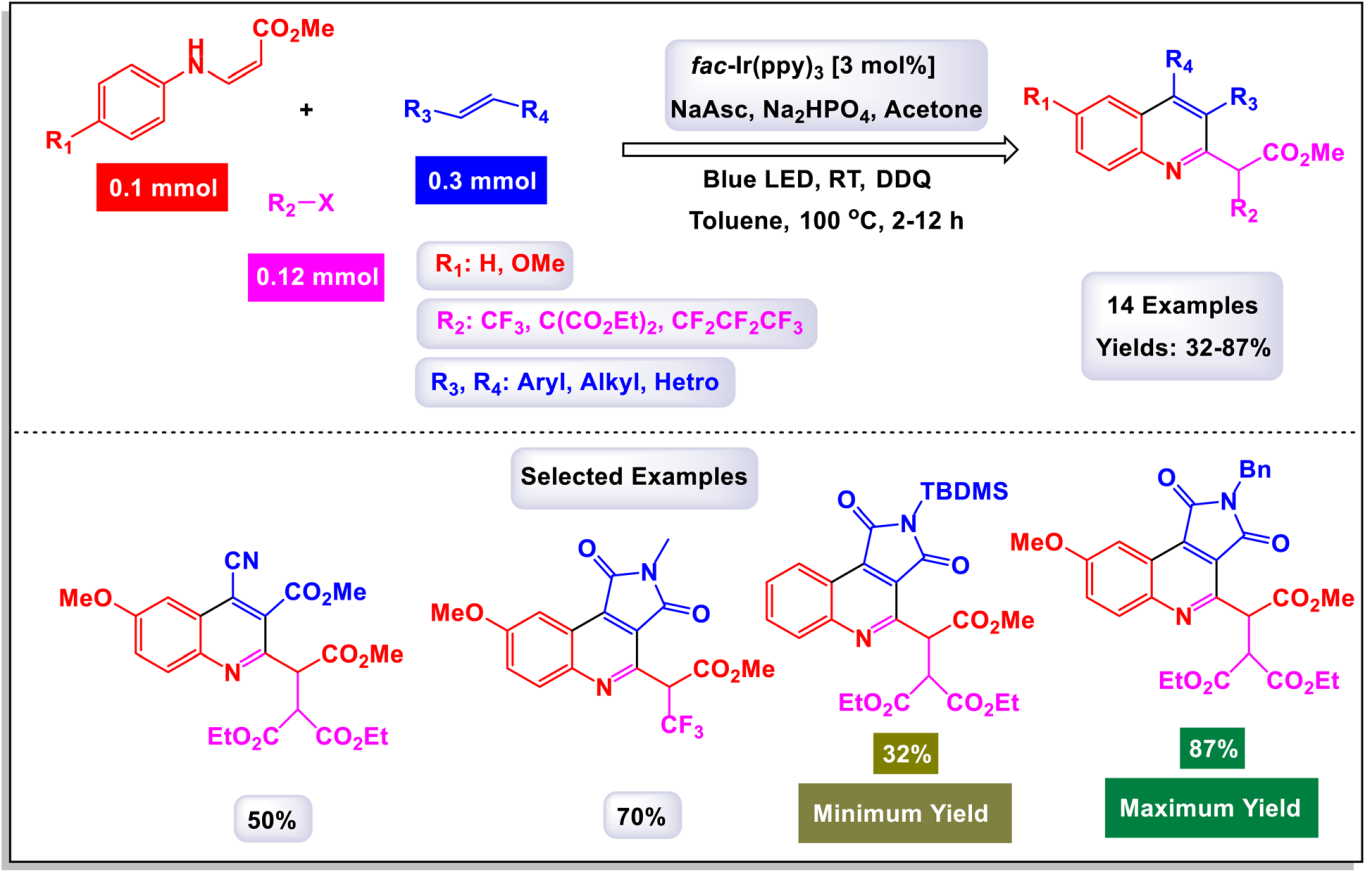
Synthesis of highly substituted quinolines [catalysis by Ir(ppy)_3_].

**Scheme 26:**
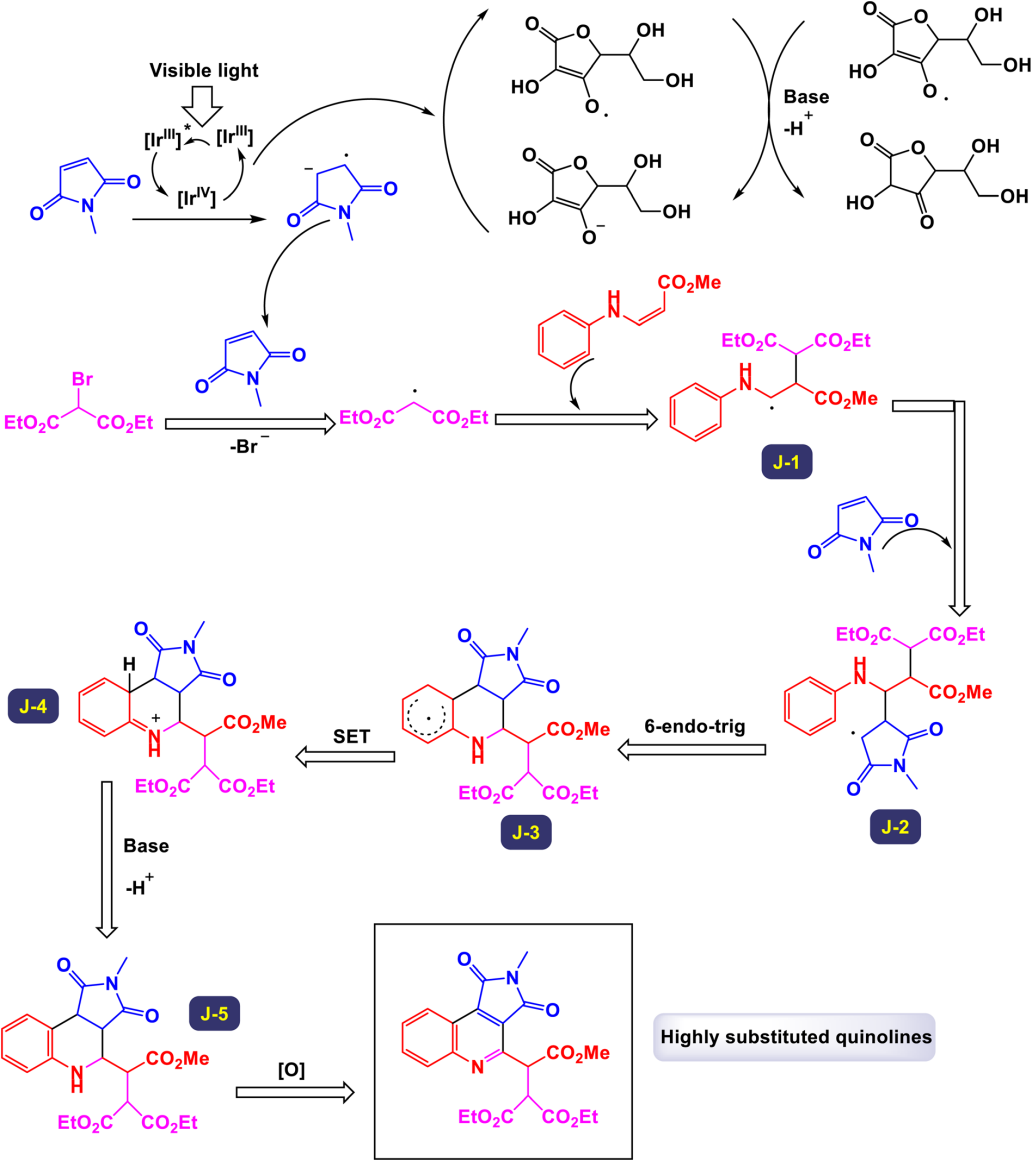
Plausible mechanism for the synthesis of highly substituted quinolines [catalysis by Ir(ppy)_3_].

In 2018, Park *et al.* made significant strides in synthetic chemistry by developing a novel method for the synthesis of highly substituted quinolines. This innovative approach relies on a three-component radical cascade that utilizes visible-light photoredox catalysis, enabling a series of reactions to occur seamlessly.^102^ The tandem coupling reaction that they designed showcases remarkable chemoselectivity, which is attributed to the distinct electronic properties of the different coupling partners involved. Specifically, when electron-rich β-aminoacrylates are subjected to this optimized reaction environment alongside electron-deficient halides and alkenes, the process yields quinolines in substantial quantities. Notably, this transformation includes an *in situ* oxidation step that converts tetrahydroquinolines into the desired quinoline products. As illustrated in Schemes 24 and 25, the researchers highlighted the effectiveness of employing maleimides with various *N*-alkyl substituents, such as benzyl and cyclohexyl groups. These modifications led to the successful formation of the corresponding quinolines, achieved consistently with good yields. This work not only advances the field of quinoline synthesis but also demonstrates the potential of harnessing photoredox catalysis for complex molecular transformations. Drawing from the mechanistic studies illustrated in Scheme 26, The reaction commences with the oxidative quenching of the excited state of [Iriii]* by the compound. This coupling generates the α-amino radical species labeled (J-1). Following this, a radical addition occurs where α-amino radical (J-1) reacts with 1-methyl-1*H*-pyrrole-2,5-dione. This step is succeeded by an intramolecular cyclization of the resulting electron-deficient radical (J-2) with an electron-rich aryl group, leading to the formation of the compound (J-3). The mechanism continues as compound (J-3) undergoes further transformations (J-4), converting into 2,3,4-trisubstituted hydroalkylquinoline (J-5) through a process that includes both oxidation and subsequent aromatization. Ultimately, a one-pot oxidation using DDQ results in the production of quinoline. Throughout this catalytic cycle, the oxidized form of the photocatalyst [Iriv] is restored by reduction with ascorbate, allowing it to continue functioning effectively. Additionally, the formation of the byproduct is believed to proceed *via* a single-electron transfer (SET) oxidation, followed by the deprotonation of the β-amino proton, highlighting the complexities of the reaction pathways involved.

**Scheme 27:**
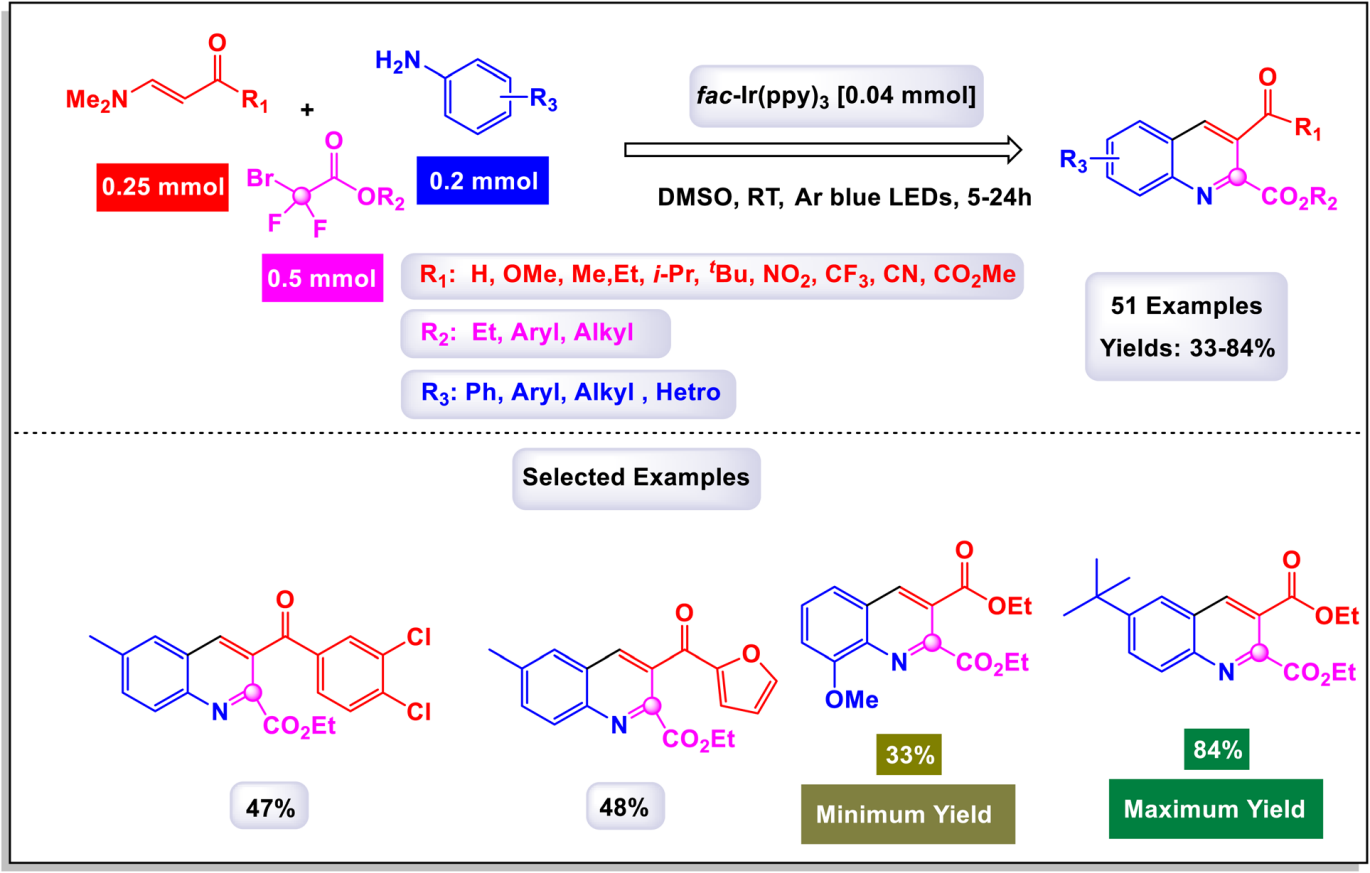
Synthesis of 2,3-difunctionalized quinolines [catalysis by Ir(ppy)_3_].

**Scheme 28:**
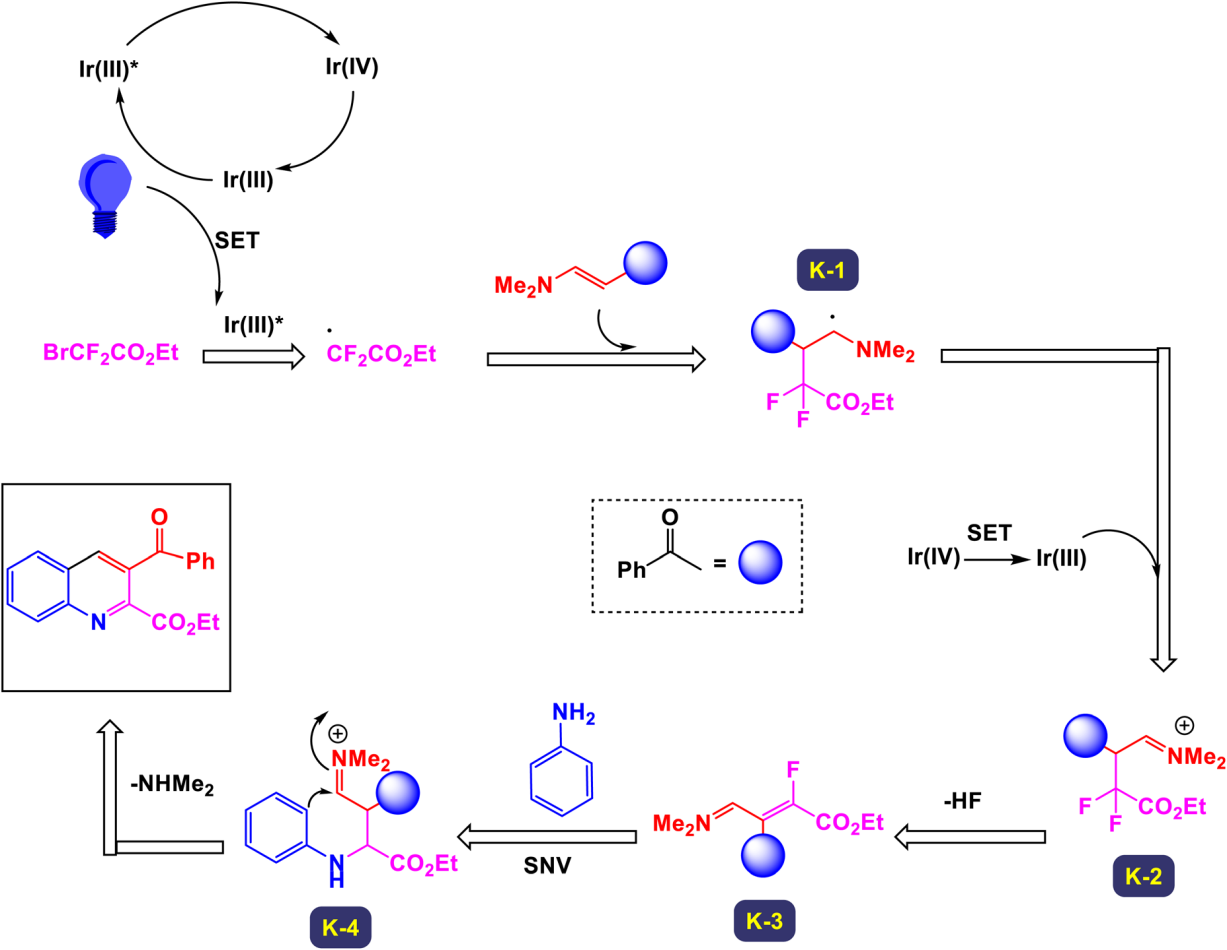
Plausible mechanism for the synthesis of 2,3-difunctionalized quinolines [catalysis by Ir(ppy)_3_].

In 2022, Geng *et al.* introduced an innovative photoinduced multicomponent reaction involving arylamines, enaminones, and difluorobromoacetates, which facilitated the synthesis of 2,3-difunctionalized quinolines.^103^ This novel approach, depicted in Scheme 27, is characterized by its remarkable tolerance to a wide array of functional groups and extensive substrate diversity, paving the way for further synthetic applications of the resulting compounds. Through detailed mechanistic investigations, it was revealed that the pivotal step in this transformation is the intermolecular [3 + 3] cyclization occurring between *in situ* generated 1,3-vinylimine ions and arylamines. Under the optimized experimental conditions, bromodifluoroalkyl compounds, derived from simple primary alcohols like methanol, dodecanol, and cyclopropylmethanol, underwent reactions smoothly, leading to the formation of cyclization products with impressive yields ranging from 33% to 84%. As illustrated in Scheme 28, the reaction mechanism is initiated with the generation of ethyl difluoroacetate radical 3a-I, which is swiftly produced *via* a single-electron transfer (SET) process from ethyl bromodifluoroacetate 3a in conjunction with the excited state of the photocatalyst [fac-Iriii(ppy)_3_]. Following this, a radical addition ensues between the resulting radical and an olefin moiety of reactant 1a, forming a two-component radical intermediate, designated as K-1. This intermediate is subsequently transformed into imine intermediate B through an oxidation process involving the fac-[Iriv(ppy)_3_] state, concurrently regenerating the ground state fac-[Iriii(ppy)_3_] for subsequent cycles of photoredox catalysis. The imine intermediate K-2 then undergoes an elimination of hydrogen fluoride (HF), resulting in the formation of intermediate K-3. This intermediate is subsequently captured by *p*-toluidine, yielding intermediate K-4. The final target product is formed through an intramolecular cyclization of K-4, followed by the elimination of *N*,*N*-dimethylamine (NHMe_2_), completing this intricate synthetic pathway.

### 2.4 Catalysis by cobalt

Cobalt catalysts provide a cost-effective, sustainable, and versatile alternative to precious metal catalysts in organic synthesis. Their ability to operate under mild conditions with high selectivity and compatibility makes them valuable tools in organic synthesis, pharmaceutical chemistry, and materials science.^104^ Cobalt-based catalysts offer several advantages for the synthesis of heterocycles, making them a valuable alternative to more traditional catalysts like palladium, platinum, or rhodium.^105,106^ The key benefits are as follows:

✓ *Cost-effectiveness and abundance*: Cobalt is significantly cheaper and more abundant than noble metals like palladium, platinum, and rhodium. This makes cobalt-based catalysts more sustainable and economically viable for large-scale applications.^107^

✓ *High selectivity and efficiency*: Cobalt catalysts often exhibit excellent regio- and chemo-selectivity in heterocycle formation. They enable selective C–H activation, allowing the direct functionalization of heterocycles without pre-functionalization.^108^

✓ *Versatility in catalytic reactions*: Cobalt catalysts have been successfully applied in various heterocycle-forming reactions, including cyclization reactions, C–H activation, hydrogenation and transfer hydrogenation, and cross-coupling reactions. They can efficiently construct nitrogen, oxygen, and sulfur-containing heterocycles.^109^

✓ *Mild reaction conditions*: Many cobalt-catalyzed reactions proceed under relatively mild conditions (lower temperatures and pressures). This reduces energy consumption and minimizes the formation of unwanted by-products.^106^

✓ *Sustainability and green chemistry*: Cobalt catalysts often enable atom-economical transformations, reducing waste generation. Some cobalt complexes operate under environmentally benign solvents, such as water or alcohols.^106^

✓ *Unique redox properties and stability*: Cobalt exhibits multiple oxidation states (Co(0), Co(i), Co(ii), Co(iii)), making it suitable for a variety of redox transformations. Many cobalt catalysts are air- and moisture-stable, enhancing their practicality.^105^

✓ *Compatibility with functional groups*: Cobalt-based catalysts tolerate a wide range of functional groups, enabling late-stage modifications of bioactive molecules and pharmaceuticals.^105^

✓ *Enabling radical and photocatalytic pathways*: Cobalt complexes can participate in radical-based mechanisms, useful for synthesizing complex heterocycles. Cobalt-based photocatalysts have been explored for visible-light-driven reactions, expanding their application scope.^110^

**Scheme 29:**
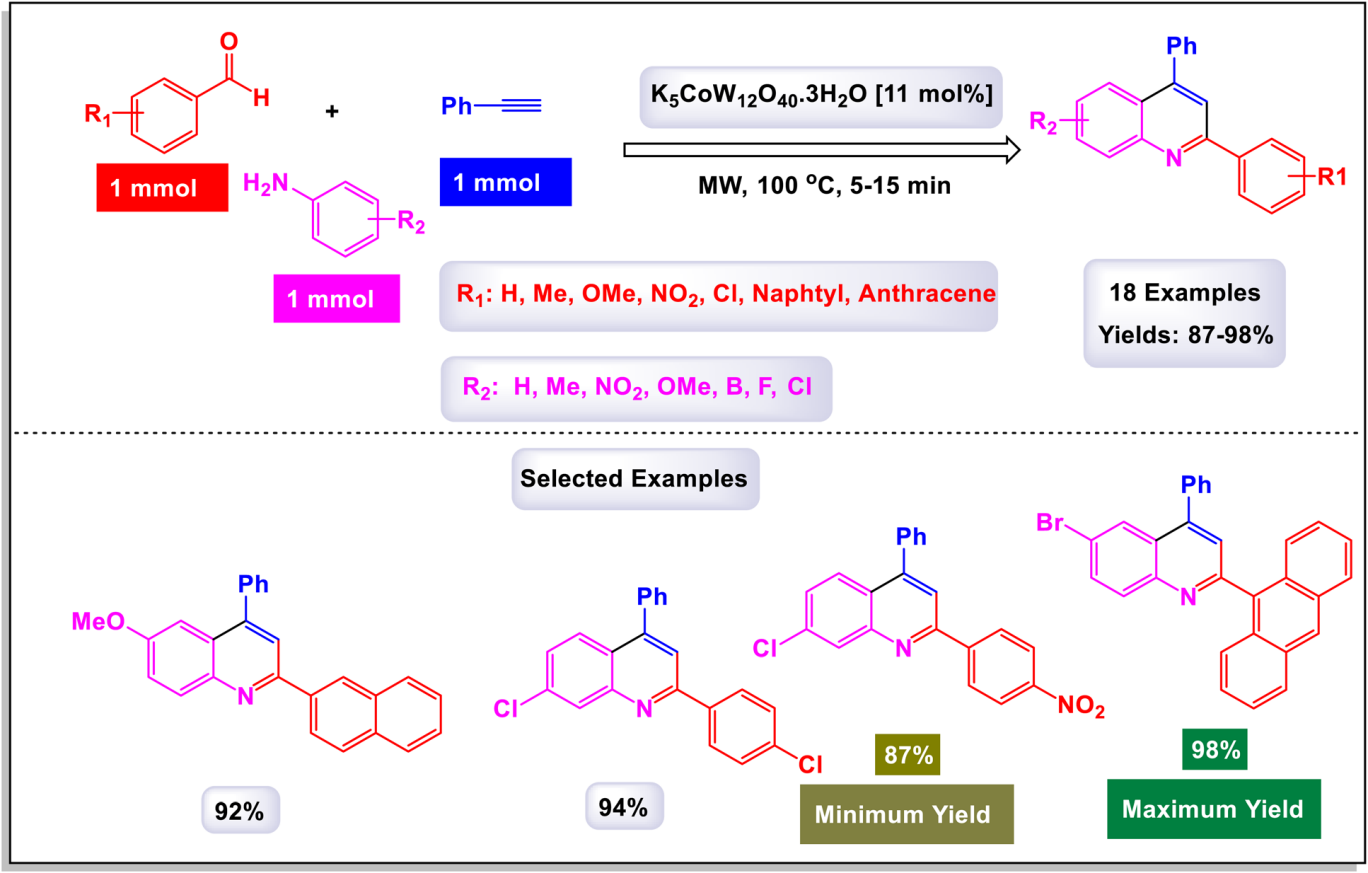
Synthesis of 2,4-difunctionalized quinolines [catalysis by K_5_CoW_12_O_40_·3H_2_O].

**Scheme 30:**
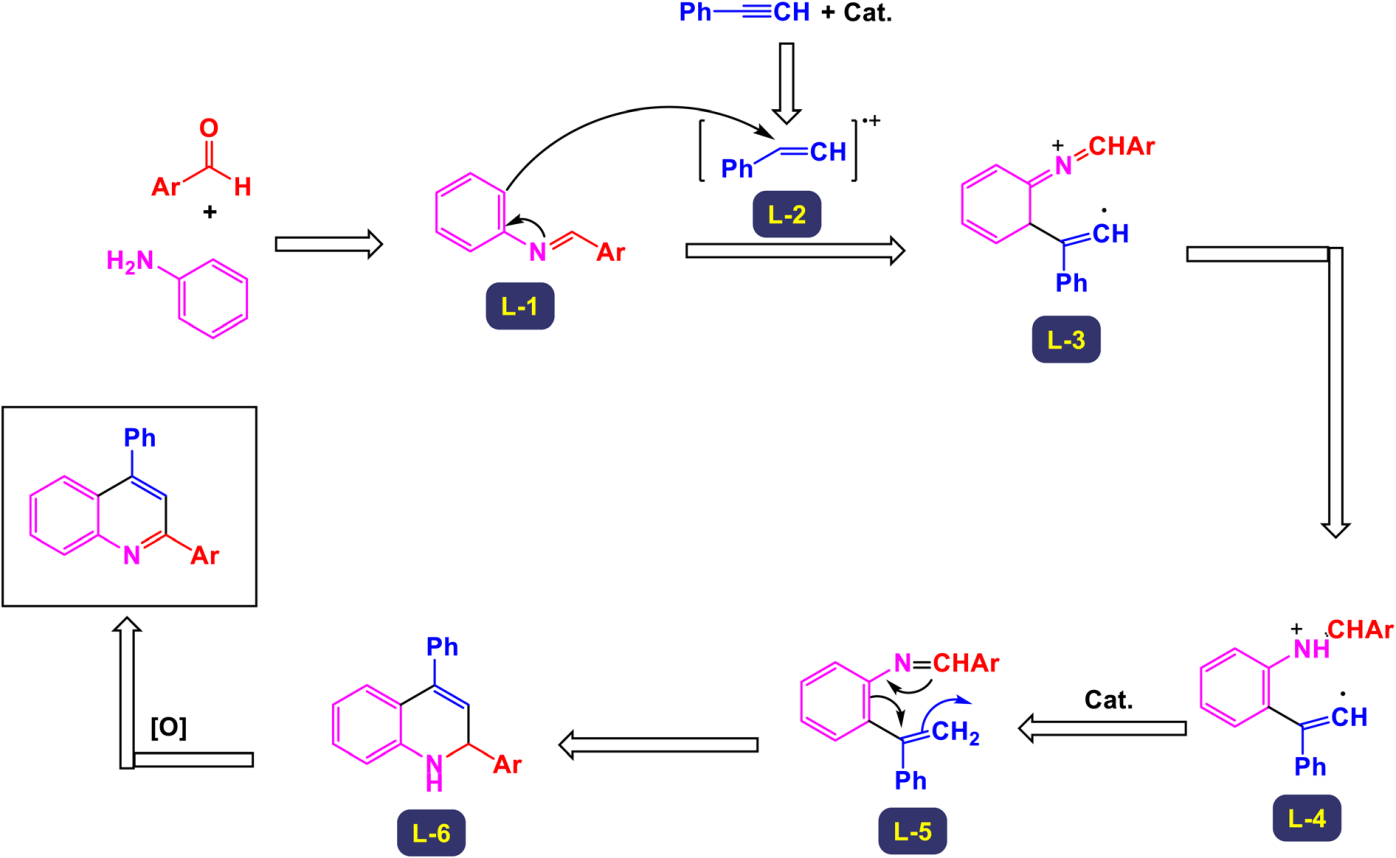
Plausible mechanism for the synthesis of 2,4-difunctionalized quinolines [catalysis by K_5_CoW_12_O_40_·3H_2_O].

In a fascinating study conducted by Mohammadpoor-Baltork *et al.*, a novel and efficient method has been developed for synthesizing quinoline and bis-quinoline derivatives through a microwave-assisted one-pot three-component reaction.^111^ This process involves the interaction of aromatic amines, aromatic aldehydes, and phenylacetylene, facilitated by small amounts of potassium dodecatungstocobaltate trihydrate (K_5_CoW_12_O_40_·3H_2_O) as a catalyst. Remarkably, this method achieves excellent yields of the desired quinoline compounds. One of the standout features of this approach is its ability to selectively convert aromatic aldehydes and arylacetylenes into their corresponding quinoline products, even in the presence of aliphatic aldehydes and alkylacetylenes. This selectivity presents significant advantages and highlights the method's practicality for synthesizing a variety of quinoline derivatives. For instance, when an equimolar mixture of phenylacetylene and 1-hexyne was subjected to microwave irradiation along with 4-nitroaniline and 4-methylbenzaldehyde in the presence of the aforementioned catalyst, phenylacetylene was transformed with remarkable chemoselectivity into its respective quinoline, yielding excellent results (Scheme 29). Similarly, 4-methylbenzaldehyde was selectively converted into its quinoline form, even when heptanal was present, further illustrating the technique's capability for maintaining high selectivity under the same conditions. This innovative method not only enhances the efficiency of quinoline synthesis but also serves as an attractive option for chemists looking to explore derivative compounds. The process outlined in Scheme 30 begins with the formation of the intermediate imine (L-1), which is generated through the condensation reaction between amine and aldehyde. Following this, an electron is transferred from phenylacetylene to the cobalt(iii) species, resulting in the creation of a radical cation intermediate, denoted as (L-2), alongside a cobalt(ii) species. Notably, the observed regiochemistry of the resulting products suggests that placing the positive charge at the benzylic carbon while the unpaired electron resides at the primary carbon of the radical cation is the most favorable arrangement. This conclusion is further substantiated by X-ray analysis, which provides clear confirmation of the regiochemistry in the final products. Next, imine (L-2) undergoes a nucleophilic attack on the radical cation (L-3), leading to the formation of compound 8. In a subsequent step, a one-electron transfer from the cobalt(ii) species to compound (L-4) occurs, accompanied by a prototropic shift from the nitrogen atom to the adjacent carbon atom. This transformation results in the formation of intermediate (L-5). The conversion of this intermediate into dihydroquinoline (L-6) is achieved through an electrocyclic reaction, which is once again followed by a prototropic shift, this time from carbon back to nitrogen. Finally, the dihydroquinoline (L-6) undergoes aromatization in ambient conditions in the presence of a catalyst, yielding the desired quinoline product. This step also facilitates the release of the cobalt(iii) species, allowing it to participate in another catalytic cycle. The catalyst can be effectively reused multiple times, with minimal degradation of its catalytic performance.

**Scheme 31:**
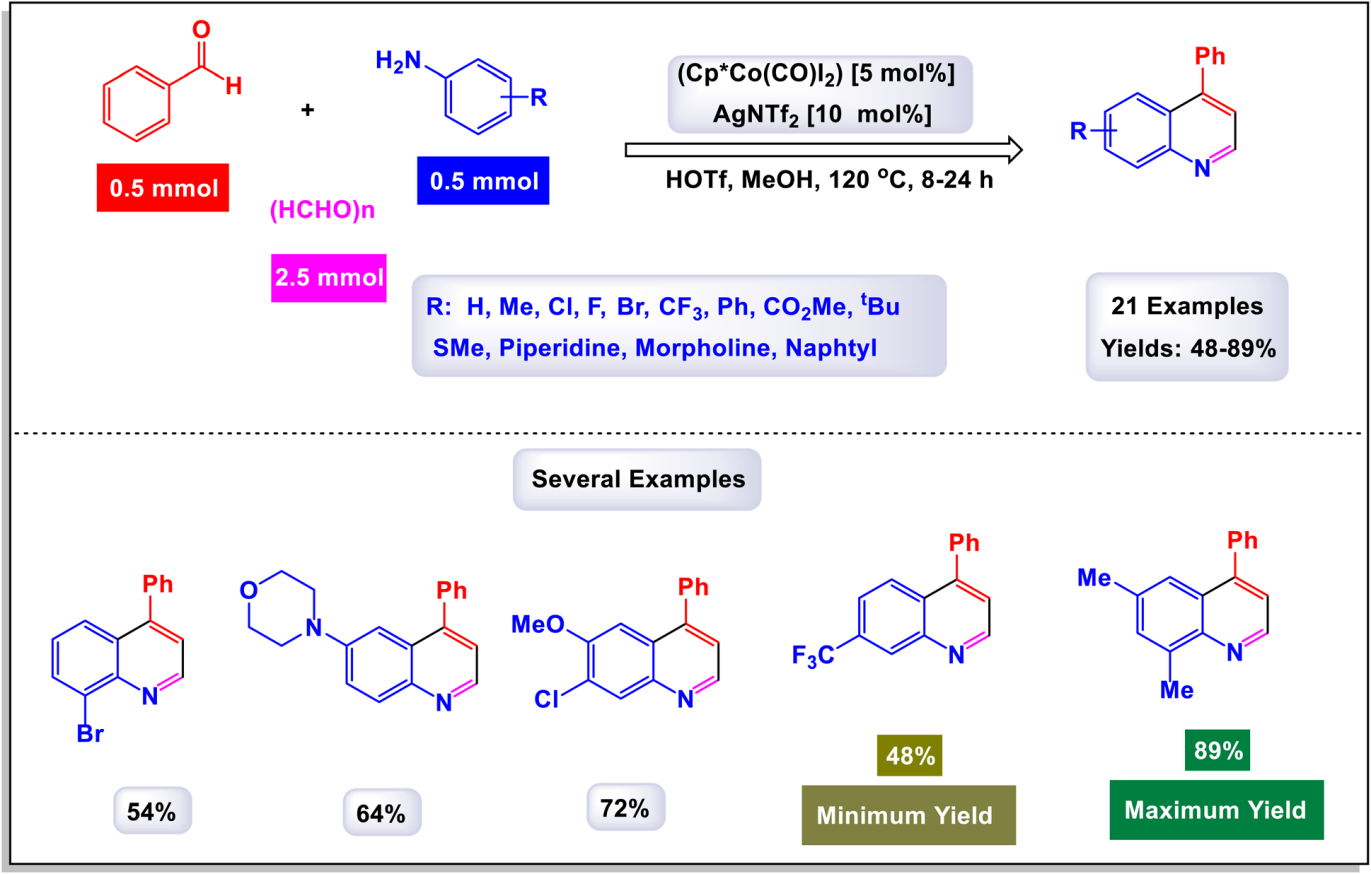
Synthesis of 4-phenyl quinolines [catalysis by Cp*Co(CO)I_2_].

**Scheme 32:**
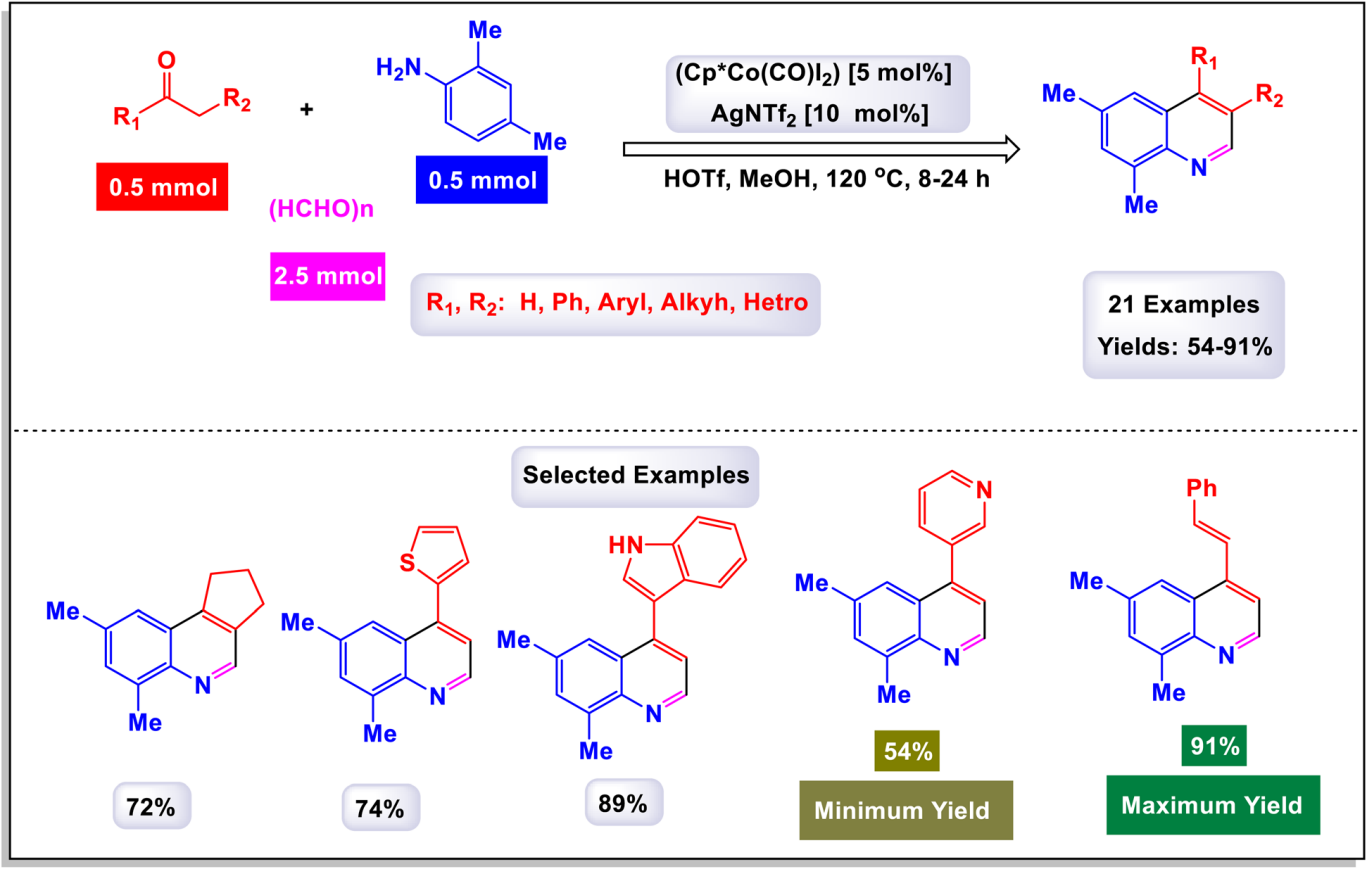
Synthesis of 3,4-difunctionalized quinolines [catalysis by Cp*Co(CO)I_2_].

**Scheme 33:**
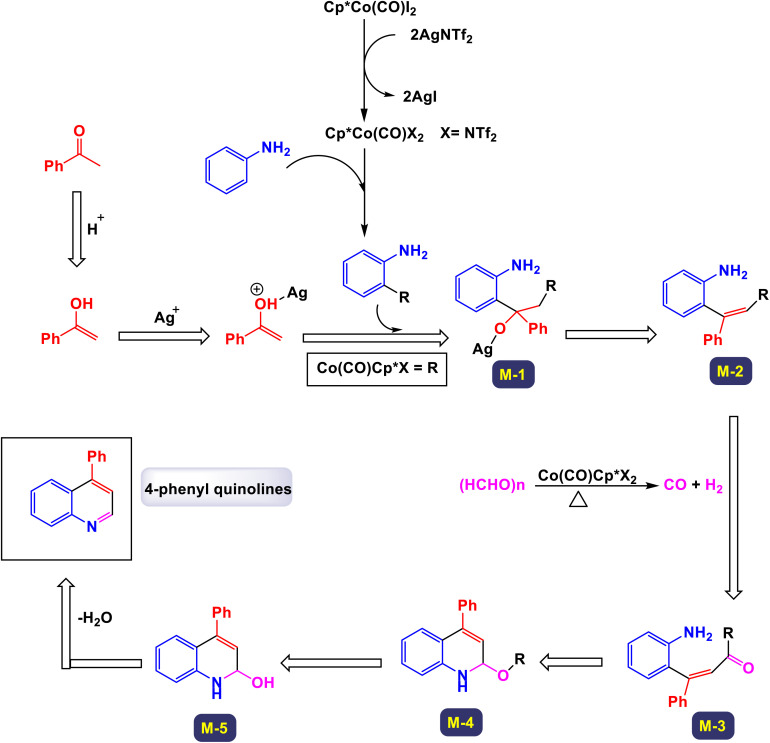
Plausible mechanism for the synthesis of 4-phenyl quinolines [catalysis by Cp*Co(CO)I_2_].

The research team led by Zhang introduced an innovative method that utilizes a cobalt(iii)-catalyzed process for C–H activation, followed by carbonylation and cyclization of anilines in conjunction with ketones.^112^ This reaction employs paraformaldehyde as a carbonyl agent, enabling access to a diverse array of quinoline compounds characterized by a wide tolerance for different functional groups, and yielding products in good to excellent quantities. Remarkably, the catalytic system demonstrated adaptability by successfully accommodating various substrates, including heteroaromatic compounds such as furan, thiophene, pyridine, and indole, as well as polyaromatic naphthalene and cycloaryl ketone substrates. These diverse starting materials were converted into their corresponding products with moderate to good yields. As illustrated in Schemes 31 and 32, larger substrates such as 4-(piperidin-1-yl)benzenamine and 4-morpholinobenzenamine were also examined and proved to be suitable. These substrates were transformed into their respective products, yielding satisfactory results. The proposed mechanism, detailed in Scheme 33, begins with the activation of the complex Cp*Co(CO)I_2_ by AgNTf2, which subsequently coordinates with substrates. This coordination sets off an electrophilic aromatic substitution (EAS) pathway, leading to *ortho*-metalation and the formation of an intermediate labeled A. Following this, a regioselective migratory insertion of the enol form of substrate aldehyde occurs, resulting in the formation of intermediate (M-1). The next step features a beta-hydride elimination, which generates an alkenyl intermediate (M-2). This intermediate then reacts with carbon monoxide, produced *in situ* from the decomposition of paraformaldehyde, yielding intermediate (M-3). An intramolecular cyclization follows, producing a cobalt(iii) alkoxide species denoted as M-4. The protonolysis of the Co–O bond in this species regenerates the active cobalt(iii) catalyst and simultaneously produces a tertiary alcohol, referred to as M-5. Finally, intermediate F undergoes intramolecular dehydration, resulting in the formation of the desired final product 3a.

**Scheme 34:**
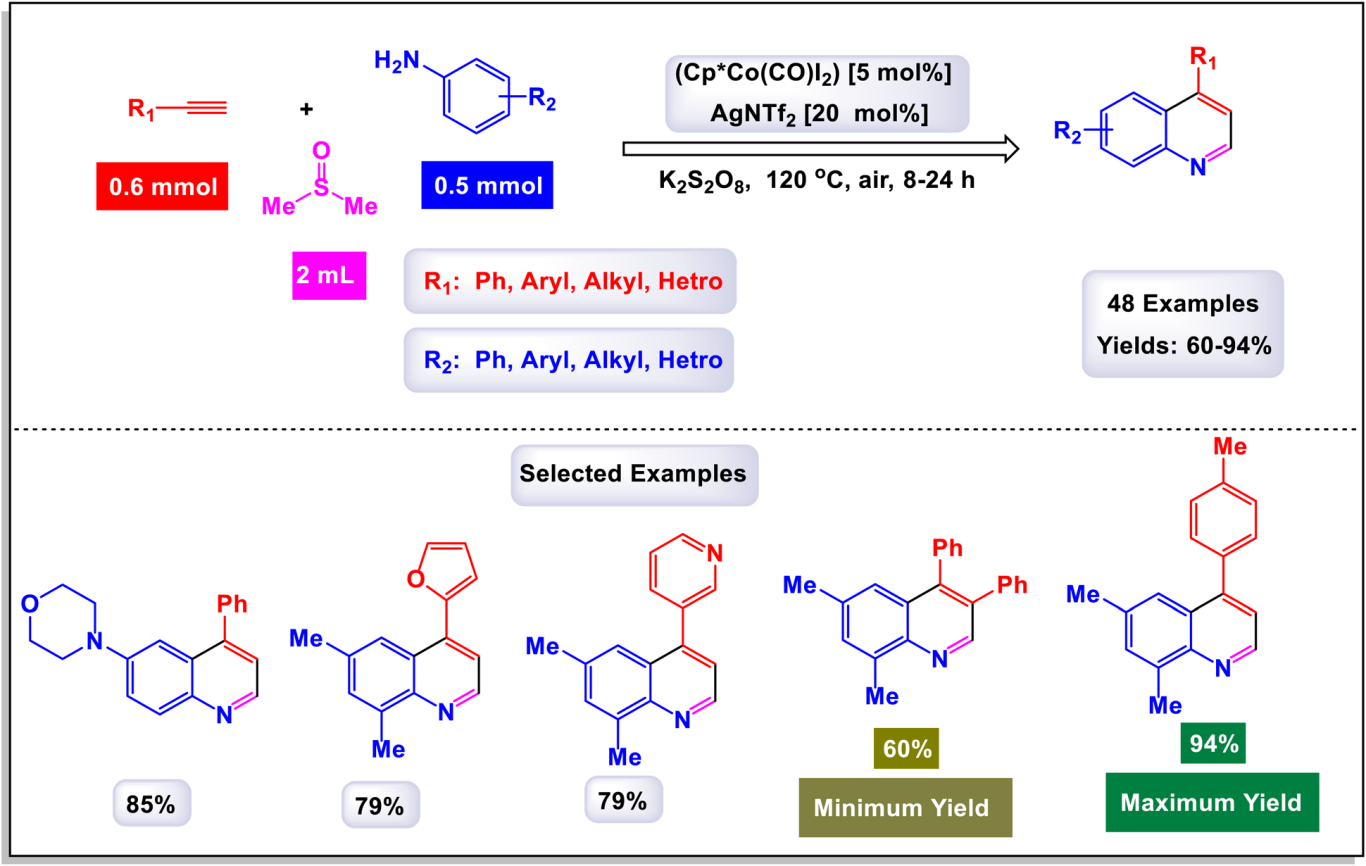
Synthesis of quinoline derivatives [catalysis by Cp*Co(CO)I_2_].

**Scheme 35:**
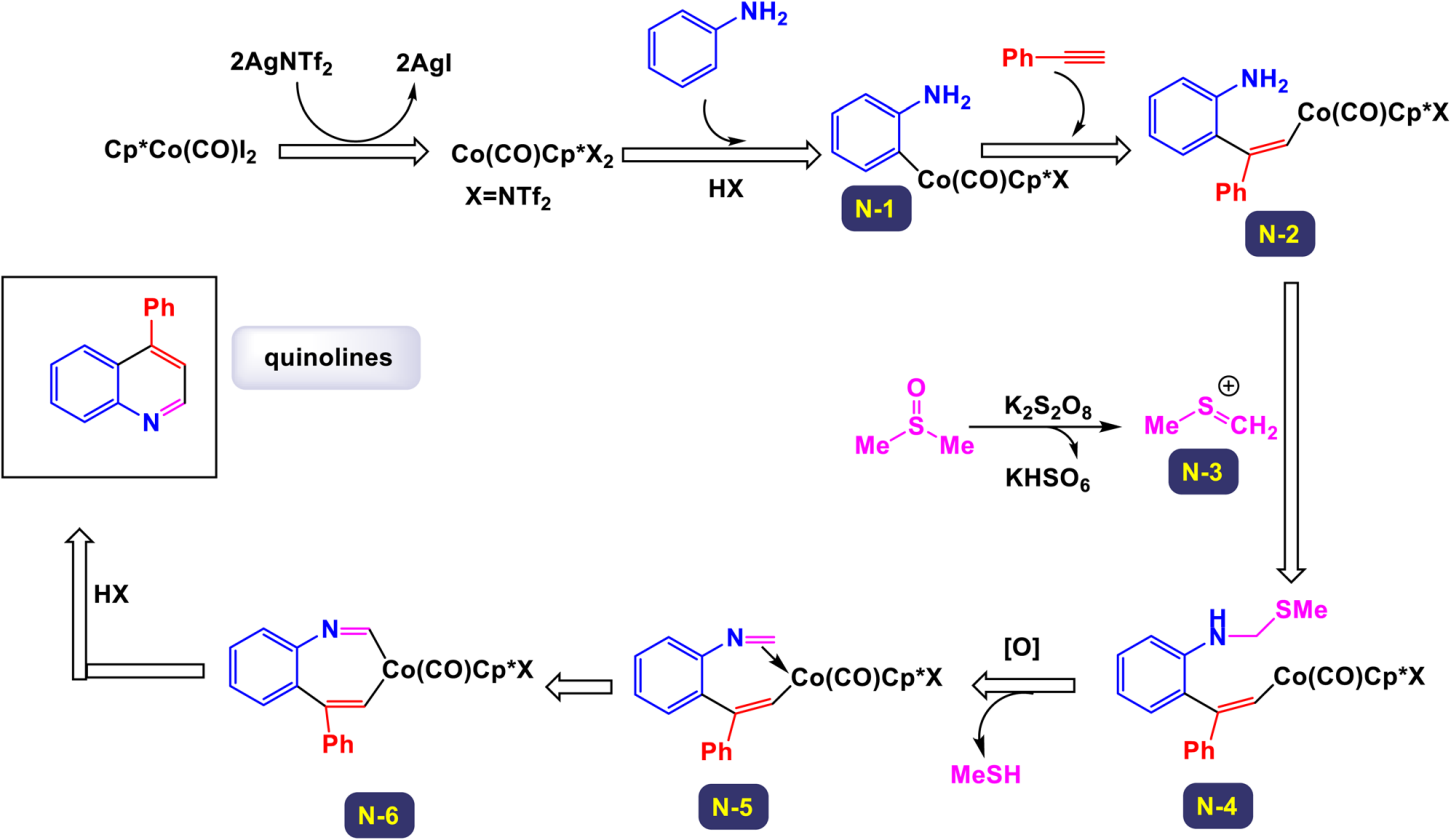
Plausible mechanism for the synthesis of quinoline derivatives [catalysis by Cp*Co(CO)I_2_].

The research team led by Yi has made significant advancements in the field of organic synthesis, specifically through a novel Co(iii)-catalyzed method that incorporates dimethyl sulfoxide (DMSO) to facilitate C–H activation and cyclization reactions.^113^ This innovative approach enables the direct and highly efficient synthesis of valuable quinoline derivatives starting from simple, economically accessible anilines and a variety of alkynes. The process is characterized by its exclusive regioselectivity and impressive tolerance for a broad range of substrates and functional groups, resulting in good to excellent yields of the desired quinoline products. Notably, DMSO plays a dual role in this reaction, acting both as the solvent and as a crucial C1 building block integral to the formation of the quinoline structure. As illustrated in Scheme 34 of the study, various substituted acetylenes—including cyclohexylacetylene, ethynylnaphthalene, *N*-propargylphthalimide, and ethynylferrocene—are effectively integrated into the catalytic system, yielding their corresponding quinoline products in moderate to good yields. The method demonstrates a remarkable compatibility with both electron-donating groups (such as methyl, methoxy, ethyl, *n*-propyl, *tert*-butyl, and dimethylamino groups), as well as electron-withdrawing groups (including fluorine, chlorine, bromine, iodine, and trifluoromethyl) when attached to the benzene ring of the alkynes. Detailed mechanistic studies conducted during the research have indicated that the reaction likely involves the formation of a reactive intermediate known as 2-vinylbenzenamine. In the proposed mechanism, as depicted in Scheme 35, the process initiates with the generation of an active cationic Co(iii) catalyst upon the treatment of Cp*Co(Co)I_2_ with AgNTf_2_. This catalyst then coordinates with the aniline substrate, leading to an electrophilic aromatic substitution (EAS) pathway. *Ortho*-Metalation occurs next, forming an intermediate referred to as N-1. The unique polarization of the carbon–cobalt bond in this intermediate promotes a 1,2-regioselective insertion of an alkyne, resulting in the formation of another intermediate, N-2. Further along the reaction pathway, the presence of K_2_S_2_O_8_ activates DMSO to yield an intermediate, N-3, which then couples with intermediate B to produce another reactive species, N-4. This intermediate N-4 undergoes oxidation, transforming into an imine species N-5 while releasing methanethiol (HSMe). Following this, the Co–C migratory insertion leads to the formation of yet another intermediate, N-6. The final step of the process involves the protonolysis of N-6, which gives rise to the desired quinoline product alongside the regeneration of the active Co(iii) catalyst. This comprehensive mechanistic understanding sheds light on the efficiency and versatility of the reaction, paving the way for future applications and improvements in synthetic organic chemistry.

### 2.5 Catalysis by palladium

Palladium catalysts offer significant advantages in the synthesis of heterocycles due to their high efficiency, selectivity, and versatility in forming carbon–carbon (C–C) and carbon–heteroatom (C–X) bonds under mild conditions.^114,115^ They enable key transformations such as cross-coupling reactions (*e.g.*, Suzuki, Heck, and Sonogashira reactions) and C–H activation, which streamline synthetic routes by minimizing steps and reducing the need for protecting groups.^116–118^ Additionally, palladium-catalyzed reactions often exhibit excellent functional group tolerance, allowing for the construction of complex heterocyclic frameworks with high yields and regioselectivity.^119^ These benefits make palladium catalysts indispensable in pharmaceutical and materials science applications.

**Scheme 36:**
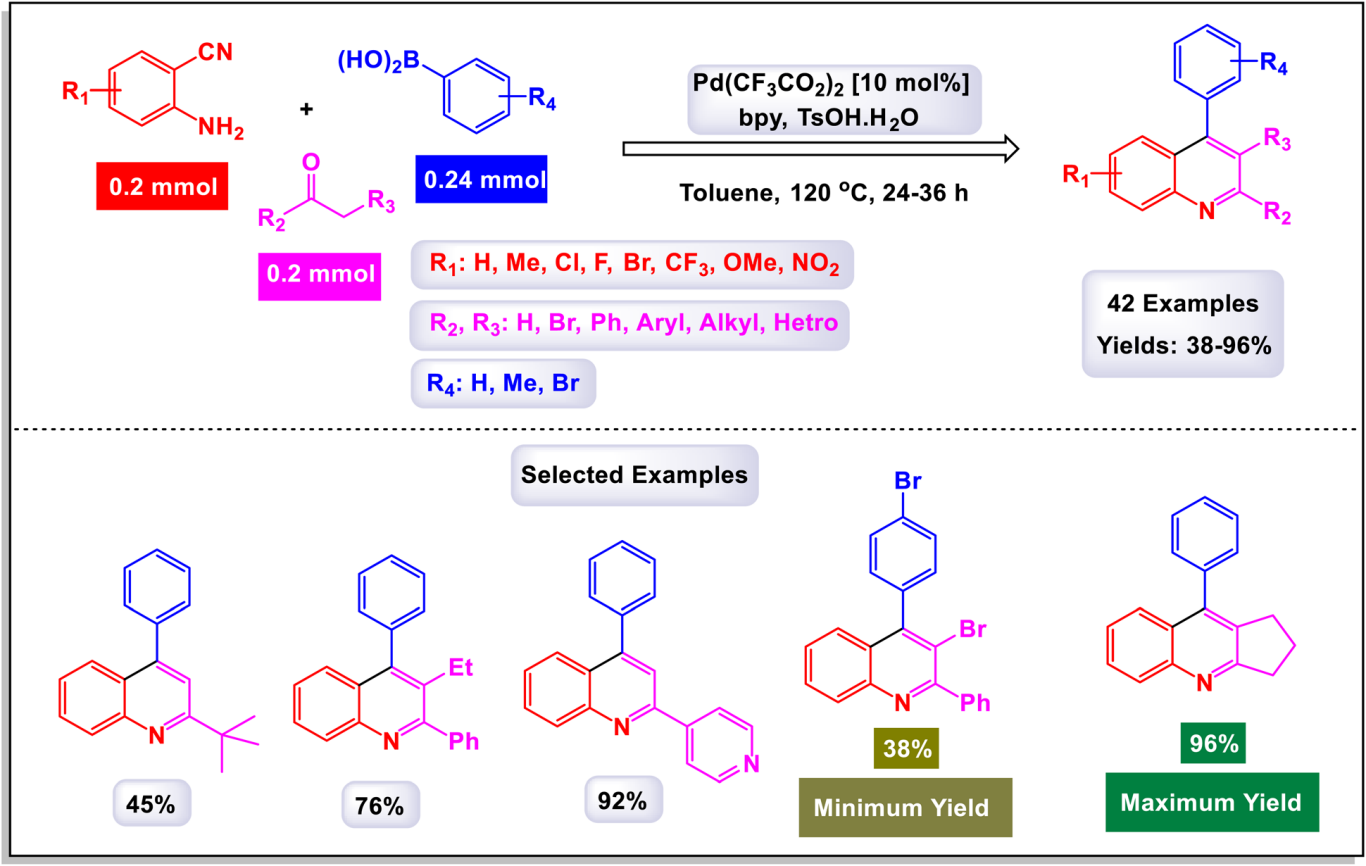
Synthesis of poly-substituted quinolines [catalysis by Pd(CF_3_CO_2_)_2_].

**Scheme 37:**
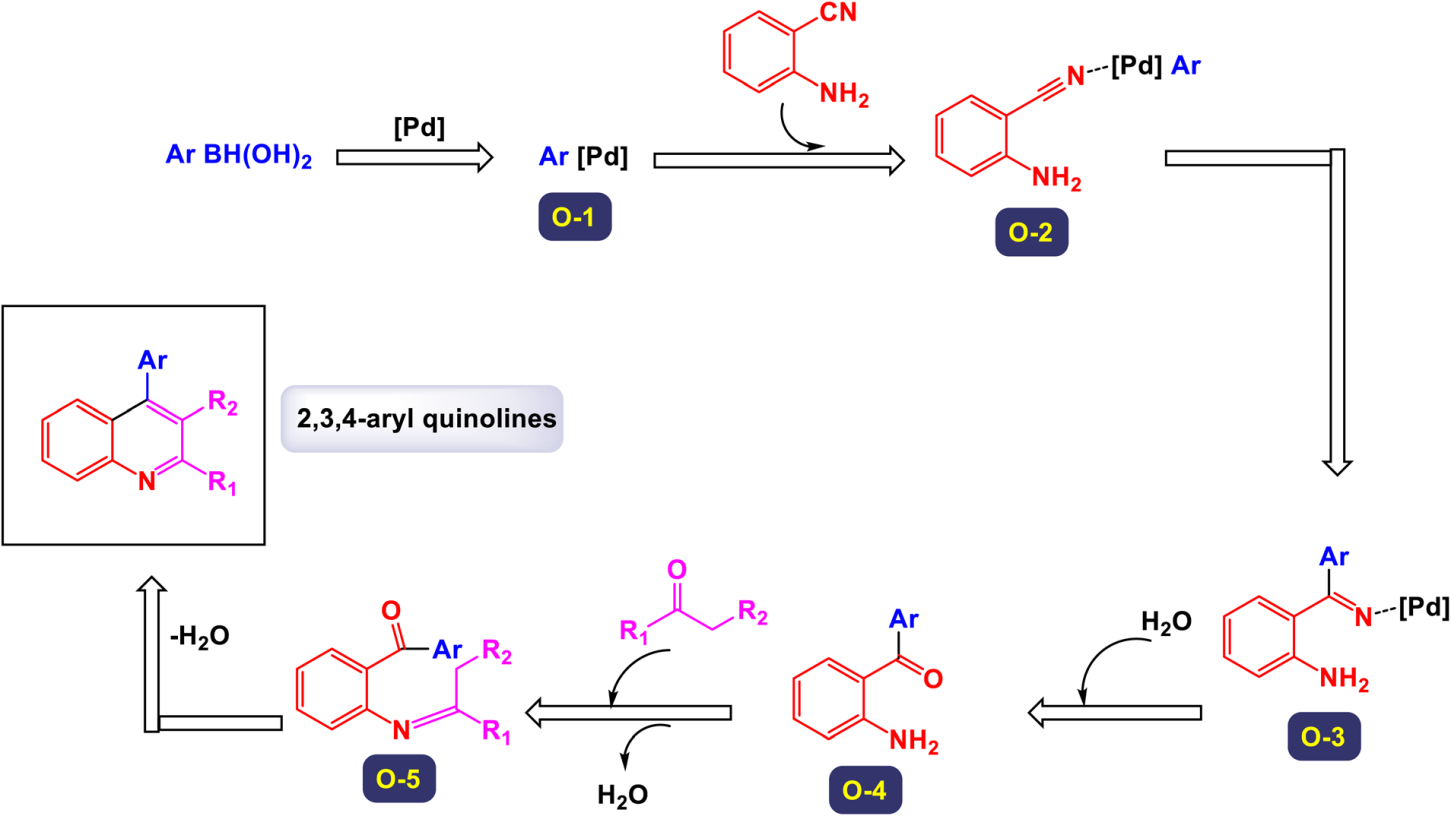
Plausible mechanism for the synthesis of poly-substituted quinolines [catalysis by Pd(CF_3_CO_2_)_2_].

In a study conducted by Chen *et al.*, a noteworthy three-component tandem reaction was developed involving 2-aminobenzonitriles, arylboronic acids, and various ketones, all facilitated by a palladium catalyst.^120^ This innovative method enables the synthesis of poly-substituted quinolines, a class of compounds valued for their diverse biological activities. As illustrated in Scheme 36, the reaction yielded promising results, with the majority of substrates yielding products in moderate to good overall yields. The study demonstrated that a wide range of arylboronic acids could be utilized, including those bearing methyl, methoxy, halogen, nitro, and trifluoromethyl groups, all of which showed compatibility with the transformation process. The reaction system also proved versatile when tested with different alkyl ketones. Specifically, compounds such as acetone, 3,3-dimethylbutan-2-one, and 1-cyclohexylethanone were employed, successfully leading to the formation of alkyl-substituted quinolines. The suggested mechanism of this reaction, as outlined in Scheme 37, provides detailed insight into the transformation's intricacies. Initially, the Pd(ii) catalyst engages in a process known as transmetalation with an arylboronic acid, resulting in the formation of an arylpalladium intermediate, designated as O-1. This intermediate exhibits the potential to coordinate with the 2-aminobenzonitrile, yielding either a nitrile *N*-bound intermediate (O-2) or a chelated form (O-2′). Following this, an intramolecular carbopalladation of the cyano group occurs, giving rise to the imine palladium complex, labeled as O-3. This step is crucial as it sets the stage for hydrolysis, facilitated by the presence of water, leading to the formation of intermediate O-4. The journey does not end here; intermediate O-4 undergoes a series of condensation and cyclization reactions (O-5), ultimately culminating in the formation of the desired polysubstituted quinolines. This strategy outlines a practical and efficient one-pot synthesis method that produces functional quinolines, achieving moderate to good yields. Importantly, this procedure demonstrates a remarkable tolerance for a variety of functional groups, making it a versatile approach for synthesizing these valuable compounds.

### 2.6 Catalysis by scandium

Scandium catalysts offer unique advantages in the synthesis of heterocycles due to their strong Lewis acidity, which facilitates highly selective and efficient transformations under mild conditions. They enable key reactions such as cyclization, ring-opening, and multicomponent coupling, allowing for the direct construction of complex heterocyclic frameworks with high regio- and stereo-selectivity.^121^ Additionally, scandium catalysts often promote reactions at lower temperatures and in environmentally friendly solvents, reducing energy consumption and waste.^122^ Their ability to activate a wide range of substrates, including oxygen- and nitrogen-containing heterocycles, makes them valuable tools in pharmaceutical and fine chemical synthesis.

**Scheme 38:**
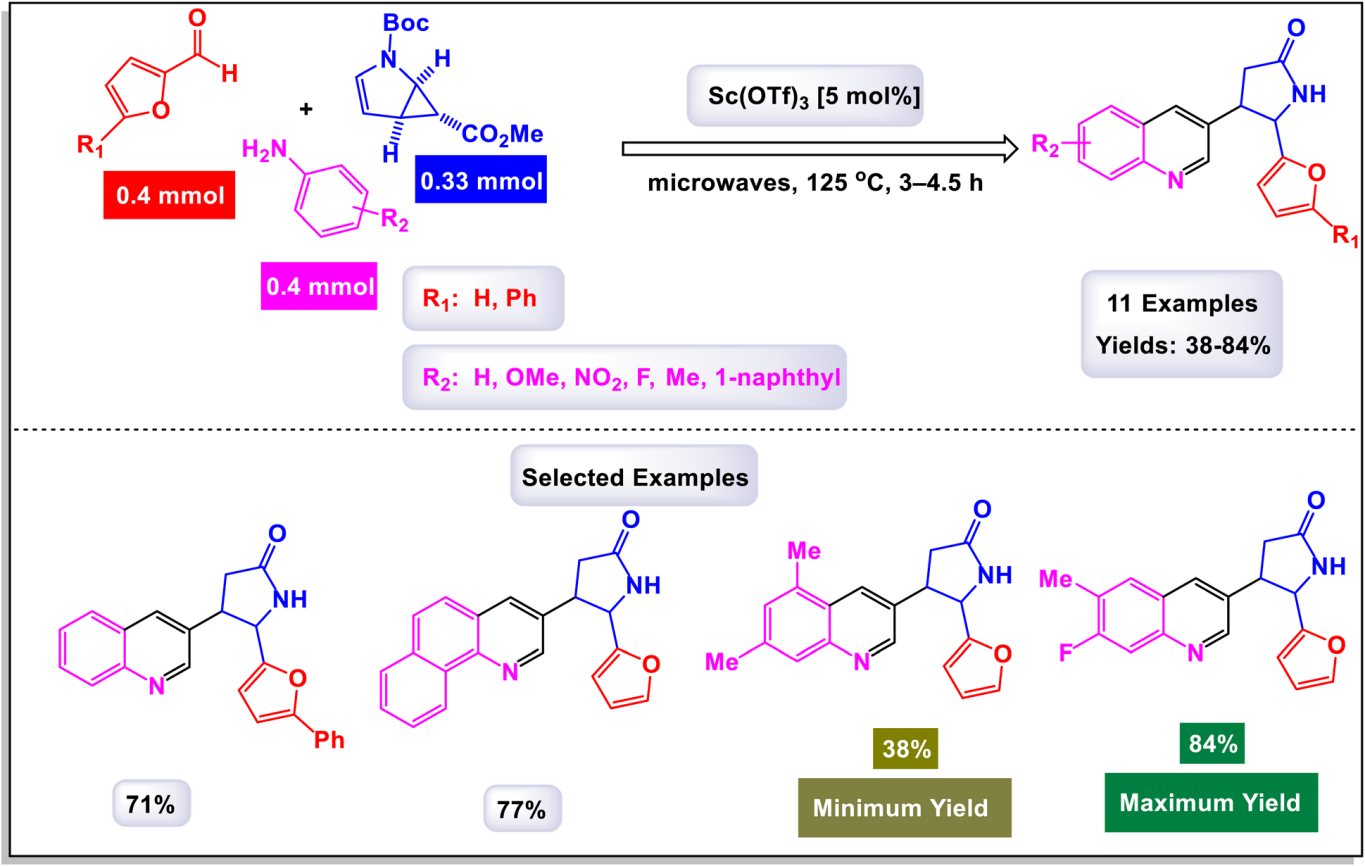
Synthesis of 3-substituted quinolines [catalysis by Sc(OTf)_3_].

**Scheme 39:**
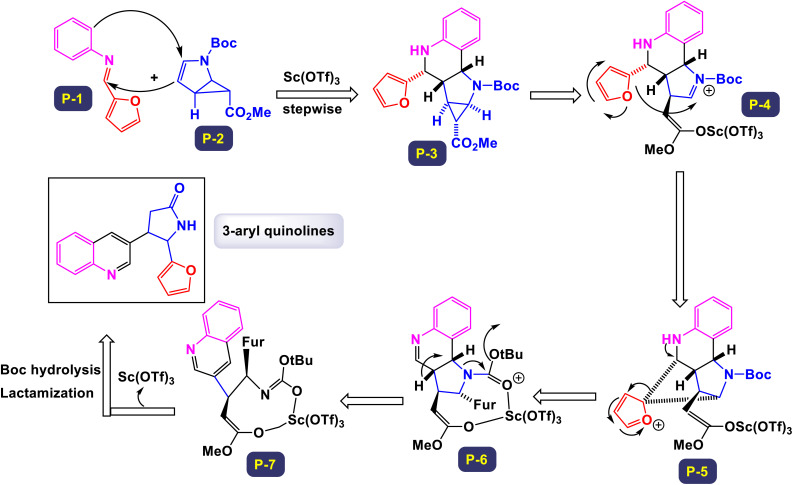
Plausible mechanism for the synthesis of 3-substituted quinolines [catalysis by Sc(OTf)_3_].

Reiser and Roy have introduced an innovative and straightforward catalytic methodology that involves a three-component assembly utilizing readily accessible furancarbaldehydes, anilines, and a specially designed monocyclopropanated adduct of *N*-Boc-pyrroles.^123^ This method successfully yields a diverse array of 3-substituted quinolines, which are produced in a stereoselective manner and with impressive yield. These compounds represent significant structural constituents found in various pharmacologically important molecules. Interestingly, when employing 3-substituted anilines in the synthesis, two potential regioisomers can arise. However, the methodology exclusively favors the formation of the sterically less-hindered quinoline, positioning the substituent in the 7-position rather than the 5-position. In cases where the more sterically hindered 5-position cannot be avoided, such as with 3,5-dimethyl aniline, there is a marked reduction in product yield (Scheme 38). The proposed mechanism for synthesizing the quinoline compounds, illustrated in Scheme 39, begins with a Povarov reaction involving the aldimine (P-1) reacting with the enamide (P-2). This reaction specifically occurs at the convex face of the enamide, leading to the formation of intermediate P-3. Notably, the bicyclic nature of enamide (P-2) plays a crucial role in determining the stereochemistry at the carbon center where the furan group is situated. This furan group is located on the endo face of the bicyclo[4.3.0] ring system (P-4), contrasting with observations from Povarov reactions involving simpler structures like 2,3-dihydrofuran (P-5) or 2,3-dihydro-1*H*-pyrroles (P-6). Due to this stereochemical arrangement (P-7), it becomes necessary for the less favored *cis*-configured aldimine to participate in the reaction if it were to proceed through a concerted cycloaddition. This indicates a greater likelihood for a stepwise reaction pathway, which would involve a Mannich reaction followed by an electrophilic substitution on the aromatic ring. This perspective aligns with recent elegant mechanistic studies involving enecarbamates as dienophiles in similar Povarov reactions.

### 2.7 Catalysis by silver

Silver catalysts offer significant advantages in the synthesis of heterocycles due to their unique ability to activate alkynes, alkenes, and C–H bonds, enabling highly selective and efficient transformations.^124^ They are particularly valuable in oxidative coupling, cyclization, and annulation reactions, facilitating the formation of diverse heterocyclic frameworks under mild conditions.^125^ Silver catalysts also exhibit excellent functional group tolerance and compatibility with various nucleophiles, making them versatile tools in complex molecule synthesis.^126^ Additionally, their relatively low toxicity and ability to work in greener solvents enhance their appeal for sustainable and pharmaceutical applications.

**Scheme 40:**
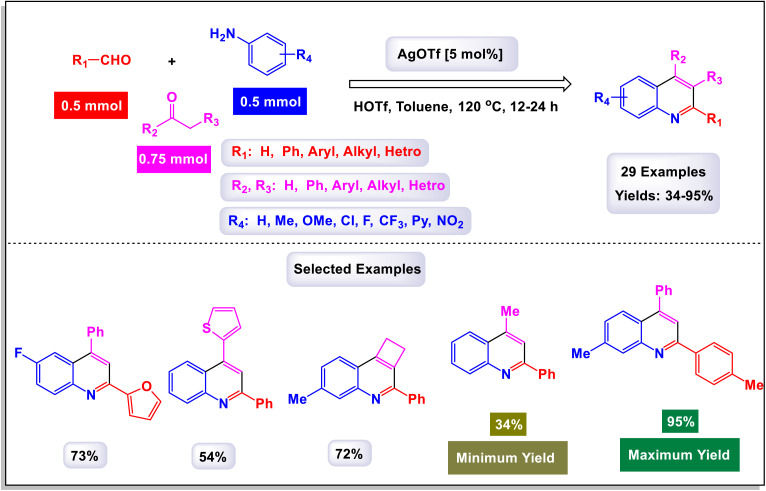
Synthesis of polysubstituted quinolines [catalysis by AgOTf].

**Scheme 41:**
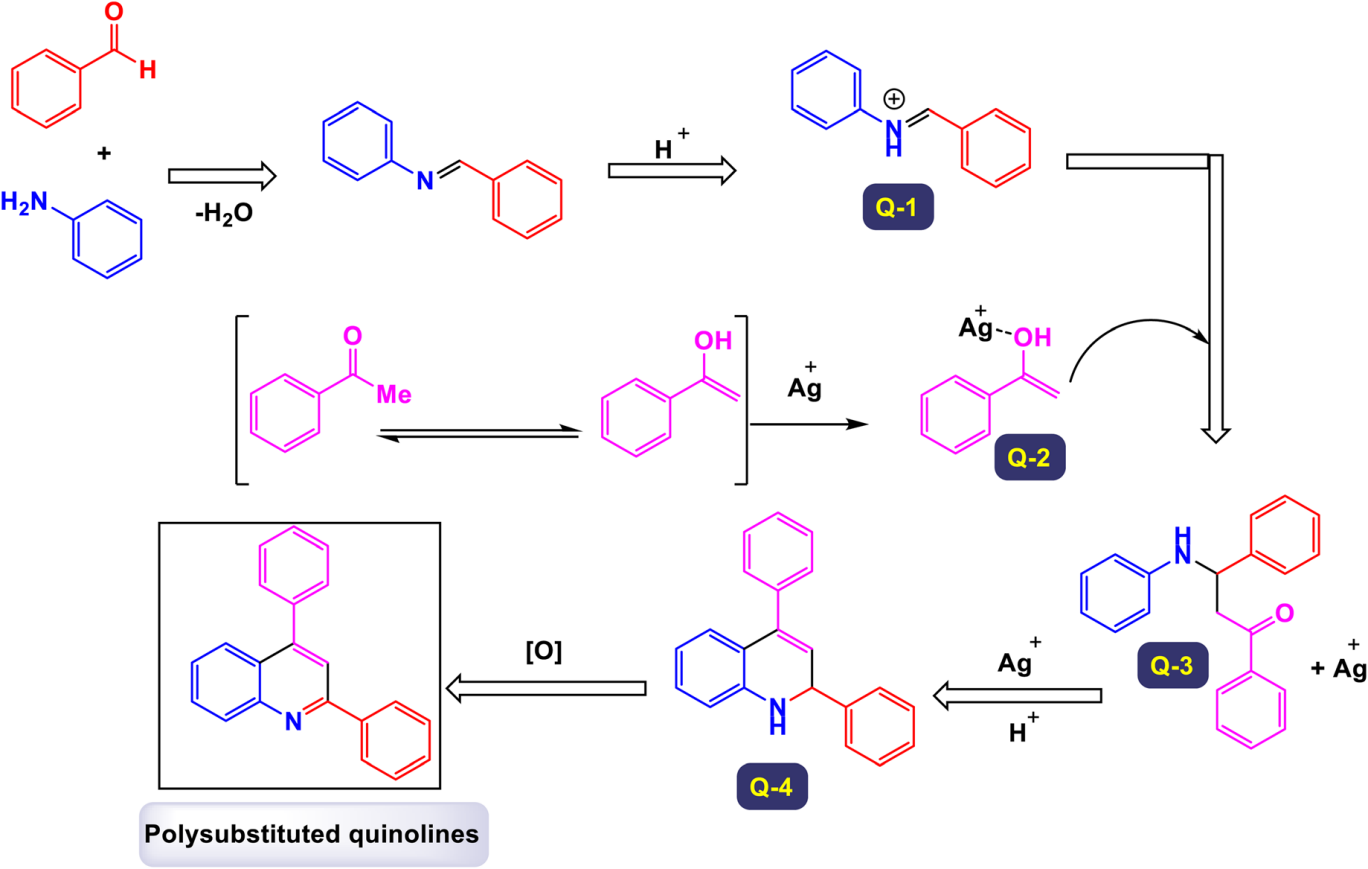
Plausible mechanism for the synthesis of polysubstituted quinolines [catalysis by AgOTf].

In a compelling study, Zhang *et al.* introduced a silver-catalyzed method for the formation of carbon–carbon (C–C) bonds, facilitating the synthesis of a diverse range of polysubstituted quinolines from various starting materials, including arylamines combined with aldehydes and ketones, as well as arylamines and 1,3-diketones.^127^ The extensive versatility of this quinoline-forming reaction was showcased through experiments using anilines featuring a wide array of substituents. These substituents included *meta*-position variants such as methyl (–CH_3_), fluorine (–F), and trifluoromethyl (–CF_3_), along with *para*-position groups such as methyl (–CH_3_), methoxy (–OCH_3_), piperidino, and trifluoromethyl (–CF_3_), all leading to satisfactory yields, as illustrated in Scheme 40. However, the electronic nature of the substituents on the amine significantly impacted the yields. Substrates possessing electron-withdrawing groups typically exhibited lower yields compared to those bearing electron-donating groups. The reactions involving aldehydes-including common choices like formaldehyde, acetaldehyde, and cyclohexanecarbaldehyde, along with heteroaryl aldehydes such as furan-2-carbaldehyde and ferrocene-2-carbaldehyde-progressed smoothly under standard conditions. These reactions yielded the anticipated quinolines in moderate to excellent yields upon isolation, demonstrating the robustness of the method, irrespective of whether the substituents on the aldehyde ring were electron-donating or electron-withdrawing. This innovative approach, utilizing a singular catalytic system for multiple chemical transformations, is significant for the advancement of new atom-economic strategies in synthetic chemistry. It illustrates an efficient pathway for constructing complex molecular architectures from straightforward starting materials while adhering to environmentally friendly practices. According to the suggested mechanism depicted in Scheme 41, acetophenone exists in equilibrium with its enol form, with this equilibrium strongly favoring the enol under the influence of silver catalyst. The generated enol swiftly reacts with an imine (Q-1), which is conveniently formed by the condensation of an amine and the aldehyde, to create an intermediate (denoted as Q-4) through a process of intramolecular cyclization (Q-2 and Q-3). Notably, oxygen from the air plays a crucial role as an effective oxidant, facilitating the aromatization of intermediate Q-4 into the final quinoline products.

### 2.8 Catalysis by ruthenium

Ruthenium catalysts offer significant advantages in the synthesis of heterocycles due to their high catalytic efficiency, functional group tolerance, and ability to facilitate diverse transformations under mild conditions.^128^ They enable selective C–H activation, cyclization, and cross-coupling reactions, which are essential for constructing complex heterocyclic frameworks with high regio- and stereo-selectivity.^129^ Additionally, ruthenium-based catalysts often operate under relatively low temperatures and in green solvents, making them more sustainable compared to traditional metal catalysts.^130^ Their stability and reusability further enhance their appeal for both laboratory and industrial-scale applications in pharmaceutical and materials chemistry.

**Scheme 42:**
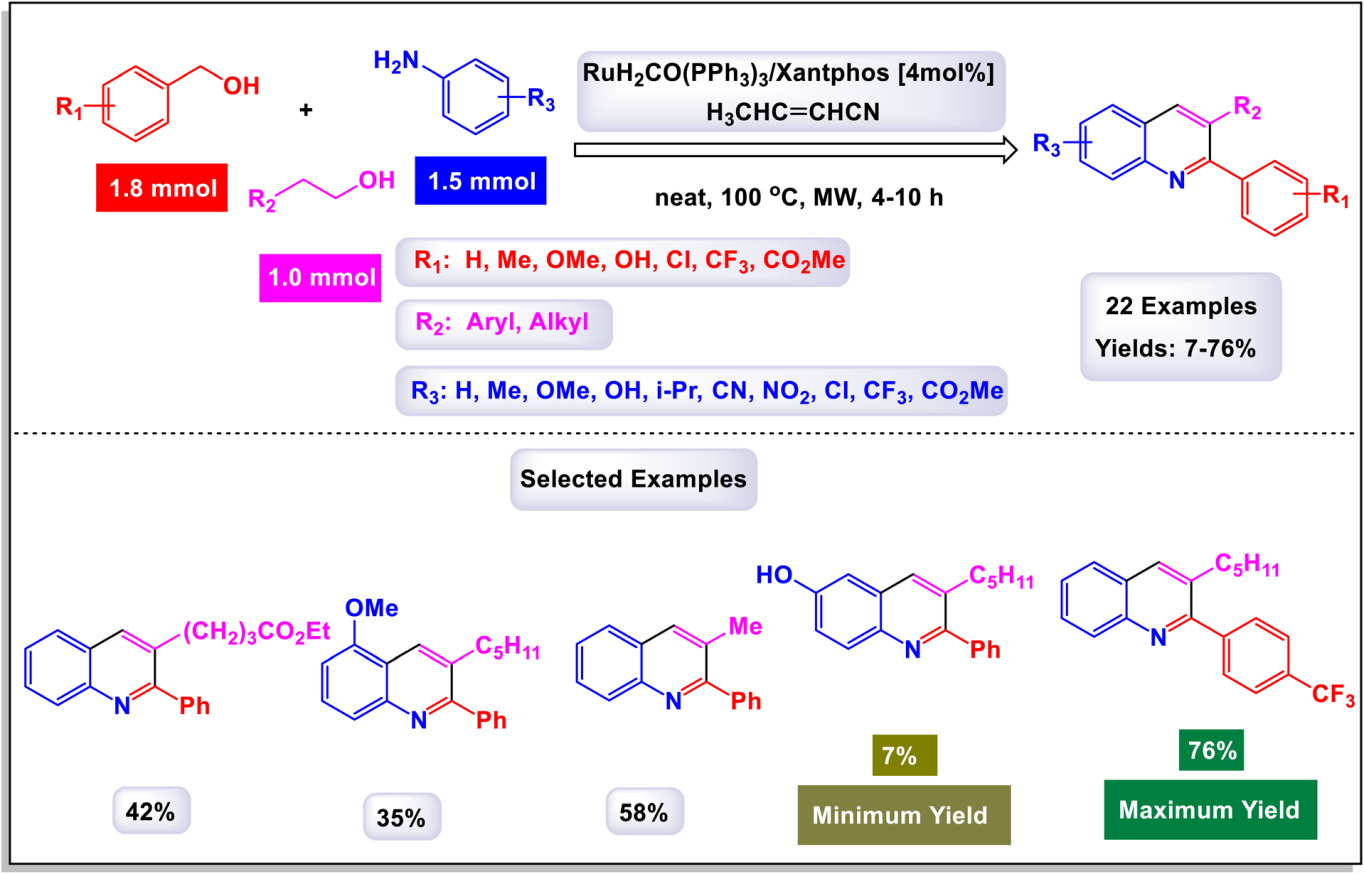
Synthesis of quinoline derivatives [catalysis by RuH_2_CO(PPh_3_)_3_/xantphos].

**Scheme 43:**
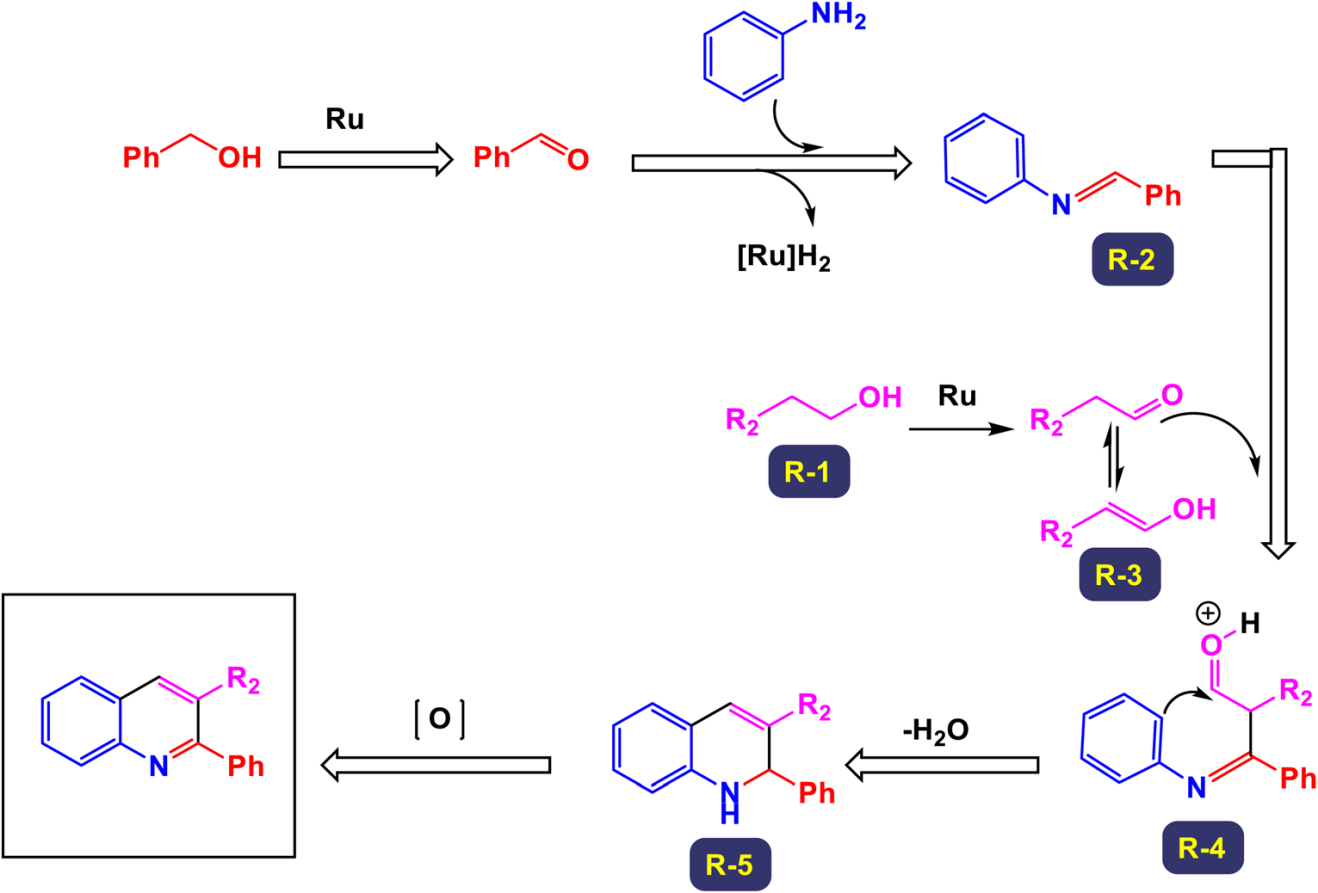
Plausible mechanism for the synthesis of quinoline derivatives [catalysis by RuH_2_CO(PPh_3_)_3_/xantphos].

Porcheddu *et al.* made significant strides in the field of organic chemistry by reporting a novel method for the ruthenium-catalyzed dehydrogenative cross-coupling of primary alcohols and imines, utilizing trifluoroacetic acid (TFA) as a co-catalyst.^37^ This innovative approach has led to the development of a diverse library of quinoline derivatives, each featuring distinct substitutions. The imines used in this process can be conveniently prepared *in situ* from a variety of anilines and multiple types of benzyl alcohols through a ruthenium-catalyzed hydrogen-transfer mechanism. As illustrated in Scheme 42, the study found that alcohols adorned with electron-withdrawing groups on their aromatic rings exhibited superior reactivity compared to those with electron-donating substituents. This observation underscores the sensitivity of the reaction to electronic effects. Interestingly, the introduction of bulky substituents at the *ortho*-position of the aromatic ring in benzyl alcohols led to a substantial decrease in the yields of the resulting quinolines. However, the reaction demonstrated remarkable versatility and tolerance towards an array of functional groups, including halogens, carboxymethyl, alkoxy, and hydroxy groups on the aromatic alcohol component. The mechanism of the reaction appears to unfold through a series of steps, commencing with an initial hydrogen transfer from the benzyl alcohol to the ruthenium catalyst. This process generates the corresponding aromatic aldehyde, along with [Ru]H_2_, as depicted in Scheme 43. The comprehensive understanding of these steps and factors contributing to reactivity provides valuable insights for further applications in synthetic chemistry. The aliphatic alcohol (R1) is transformed through a ruthenium-assisted oxidative dehydrogenation reaction, yielding the target aliphatic aldehyde. In this process, the enol (R-2) acts as a nucleophile and attacks the protonated imine (R-3), resulting in the formation of intermediate compound R-4. This intermediate then undergoes a subsequent heteroannulation reaction, ultimately leading to the creation of the 1,2-dihydroquinoline (R-5), which possesses a fused ring structure typical of quinoline derivatives.

### 2.9 Catalysis by zinc

Zinc catalysts are highly advantageous in the synthesis of heterocycles due to their low toxicity, cost-effectiveness, and environmentally friendly nature.^13,131^ They facilitate a wide range of organic transformations, including cyclization, condensation, and coupling reactions, often under mild conditions.^132^ Zinc-based catalysts exhibit excellent chemoselectivity and functional group compatibility, making them particularly useful for constructing diverse heterocyclic frameworks with high yields. Additionally, their Lewis acid properties enhance reaction rates and selectivity by activating electrophilic centers, promoting efficient bond formation.^133,134^ Due to their biocompatibility and sustainability, zinc catalysts are widely employed in pharmaceutical and green chemistry applications.

**Scheme 44:**
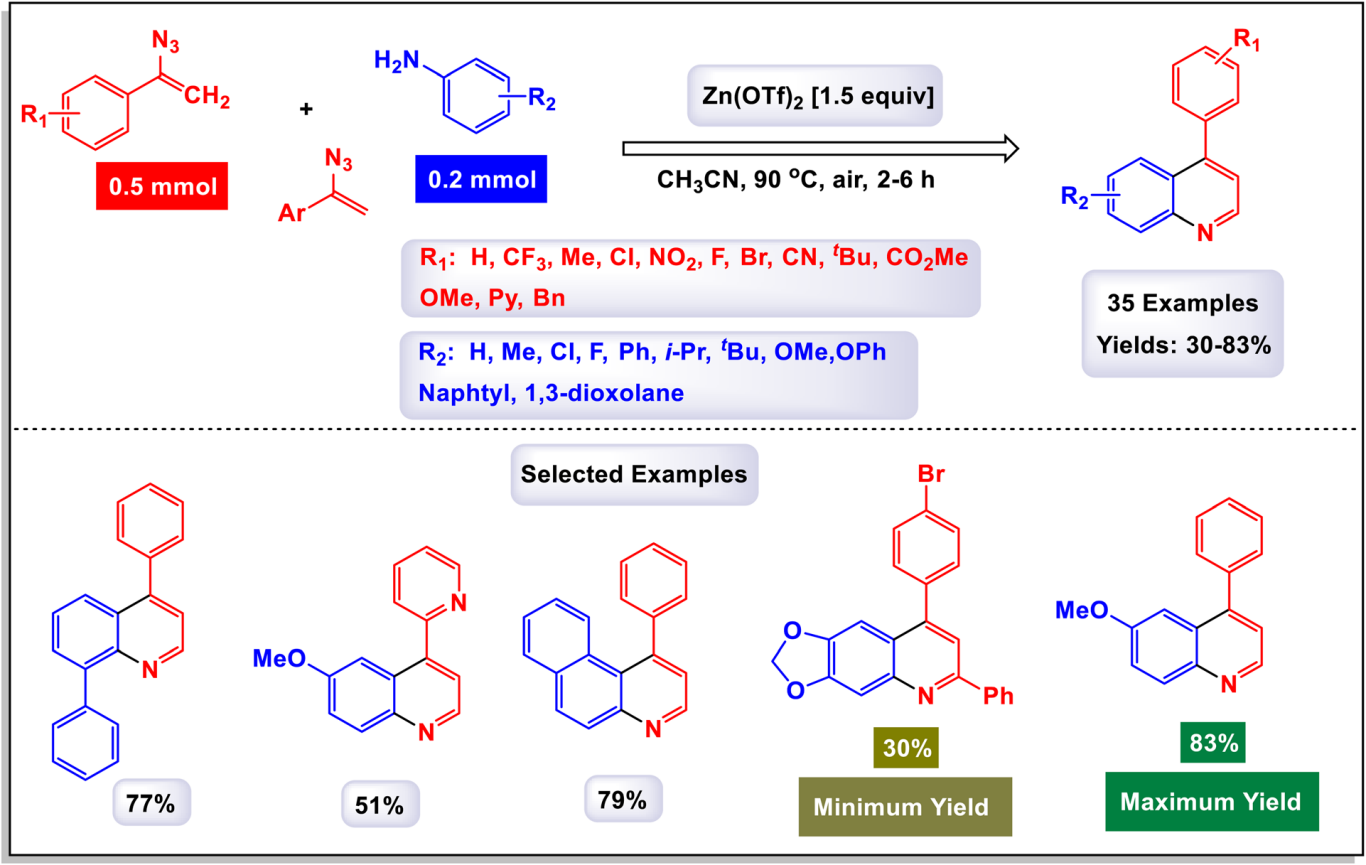
Synthesis of 4-substituted quinolines [catalysis by Zn(OTf)_2_].

**Scheme 45:**
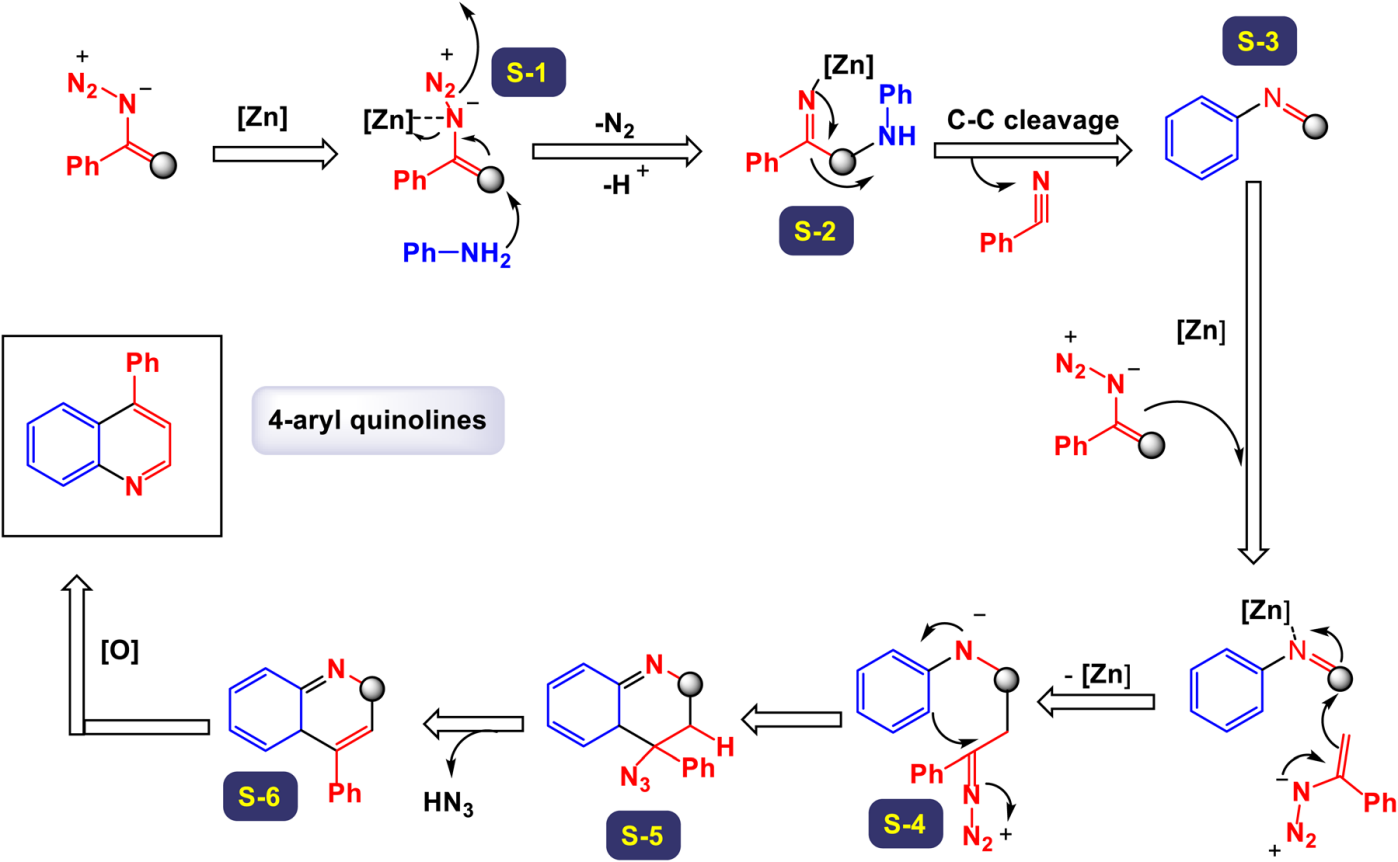
Plausible mechanism for the synthesis of 4-substituted quinolines [catalysis by Zn(OTf)_2_].

The research team led by Jiang has made significant strides in the field of organic synthesis by developing an innovative method for the selective cleavage of vinyl azides, facilitating the synthesis of 4-substituted quinolines.^135^ In this groundbreaking approach, vinyl azides serve as a dual synthon, where both C=C and C–N bonds are cleaved to yield two C=C bonds and one C=N bond, all in a single, efficient step. This reaction is particularly noteworthy due to its use of readily available substrates, remarkable step economy, mild reaction conditions, and the unique application of air as the sole oxidizing agent. Interestingly, when naphthalen-2-amine was incorporated into this transformation, the cyclization occurred at the more sterically congested α-position rather than the β-position. This outcome highlights the crucial role of electronic factors in influencing the reaction pathway, overshadowing the effects of steric hindrance. Moreover, it was observed that vinyl azides bearing electron-deficient substituents such as fluorine (F), chlorine (Cl), and bromine (Br) at various aryl positions showed excellent compatibility, leading to the formation of the corresponding halo-substituted products. These products have the potential for further coupling reactions, widening their utility in synthetic applications. In addition, vinyl azides that incorporate strong electron-withdrawing groups such as trifluoromethyl (CF3) and cyano (CN) groups, along with CH_3_COO, were also found to be effective substrates in this process (Scheme 44). Remarkably, even vinyl azides containing pyridine moieties achieved a successful outcome, producing the desired product with a yield of 51%. On a less favorable note, α-alkyl vinyl azides did not yield the desired quinoline product, likely due to their reduced reactivity. According to the proposed mechanism outlined in Scheme 45, it was determined that the nitrogen atom in the resultant quinoline originates from the aniline starting material. However, when only β-methyl vinyl azides were utilized, the reaction failed to produce the desired product, indicating that the presence of terminal vinyl azides is crucial for the successful completion of this transformation. The process begins with a nucleophilic attack by aniline on intermediate S-1, leading to the formation of intermediate S-2 while simultaneously releasing nitrogen gas. This intermediate B then undergoes a transformation to generate the imine intermediate C through the cleavage of a carbon–carbon bond, resulting in the production of benzonitrile as a byproduct, which has been identified through GC-MS analysis. Following this, intermediate S-3 engages in an intramolecular cyclization reaction known as a [4 + 2]-annulation of S-5 with the azide, which results in the creation of intermediate E. As the reaction progresses, intermediate S-5 further evolves into intermediate S-6 through the elimination of hydrazoic acid (HN_3_). In the final step, the newly formed intermediate S-6 undergoes an aromatization reaction facilitated by the presence of molecular oxygen (O_2_) from the air, yielding the target product: a highly valuable quinoline compound.

**Scheme 46:**
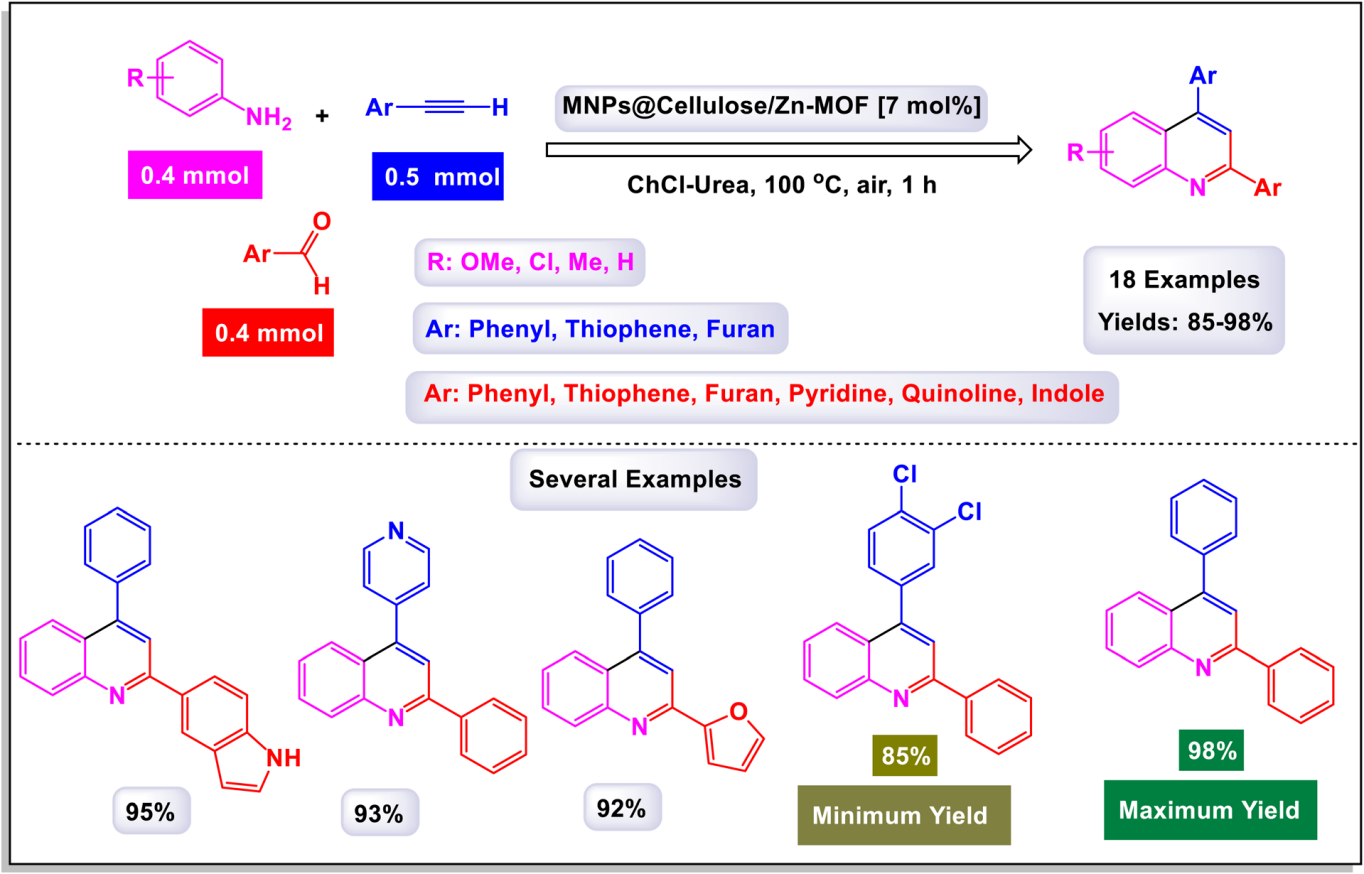
Synthesis of 2,4-diaryl quinolines [catalysis by MNPs@Cellulose/Zn-MOF].

**Scheme 47:**
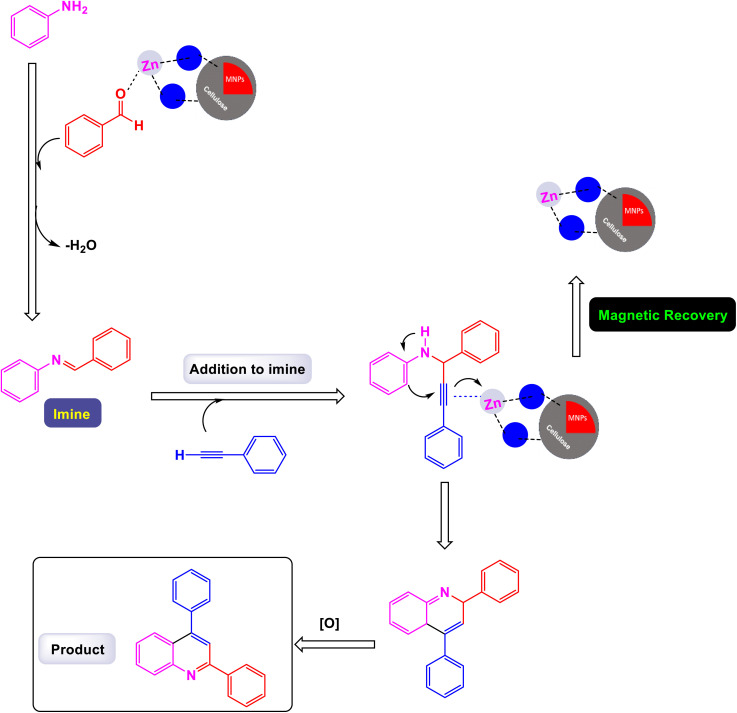
Plausible mechanism for the synthesis of 2,4-diaryl quinolines [catalysis by MNPs@Cellulose/Zn-MOF].

In a recent breakthrough, Kazemi *et al.* unveiled a pioneering synthesis method for 2,4-diaryl quinolines—an approach that is not only efficient but also environmentally sustainable.^136^ Utilizing a recyclable magnetic metal–organic framework (magnetic-MOF) zinc nanocatalyst, this innovative process unfolds in a deep eutectic solvent composed of choline chloride and urea (ChCl–Urea). The findings illustrated in Scheme 46 highlight how the variation of functional groups impacts the reaction, revealing a commendable tolerance for both electron-donating and electron-withdrawing substituents. The presence of electron-donating groups, like methoxy (–OMe) and methyl (–Me) groups, significantly enhances the reaction efficiency, leading to remarkable yields ranging from 97% to an impressive 90%. These electron-rich groups elevate the electron density, effectively stabilizing the reaction intermediates and accelerating the formation of the desired products. Conversely, while electron-withdrawing groups such as chlorine (–Cl) and nitro (–NO_2_) slightly reduce electron density, they still support notable product formation, yielding between 85% and 91%. Another significant discovery demonstrates the catalytic system's superb compatibility with a variety of heterocyclic substrates, including derivatives of quinoline, furan, and thiophene. These heterocyclic compounds yield striking results, with efficiencies soaring between 92% and 98%. This indicates the catalyst's remarkable ability to manage heteroatoms like nitrogen, oxygen, and sulfur within these intricate ring structures, all while maintaining high efficiency. The extensive compatibility of this catalyst is essential for its application in the realms of pharmaceuticals and materials chemistry, where functionalized quinolines play a crucial role. The analysis of the substrate scope reveals impressive reactivity across both mono- and di-substituted aryl groups, exhibiting minimal influence from steric hindrance. Unsubstituted aryl substrates consistently achieve high yields of 93% to 98%, while bulkier disubstituted versions still deliver respectable yields, hovering around 85% to 88%. The suggested mechanism for synthesizing 2,4-disubstituted quinolines, catalyzed by the MNPs@Cellulose/Zn-MOF nanocomposite, is beautifully illustrated in Scheme 47. Here, the Zn ions play a pivotal role, stabilizing the transition state and effectively lowering the activation energy of the reaction. Following this stabilization, the mechanism progresses through an elegant intramolecular cyclization, resulting in a partially structured quinoline framework. Notably, the magnetic properties of the catalyst provide a remarkable advantage, allowing for the recovery and reuse of the catalyst up to eight times without any significant loss in activity, underscoring the sustainability and practicality of this method.

### 2.10 Catalysis by bimetallic or alloy catalysts

Metal/metal catalysts, often in the form of bimetallic or alloy catalysts, offer significant advantages in the synthesis of heterocycles by combining the unique properties of two metals to enhance catalytic activity, selectivity, and stability.^137^ These catalysts facilitate key transformations such as cross-coupling, hydrogenation, and cyclization with improved efficiency compared to single-metal systems.^138^ The synergistic interactions between the metals can lower activation energy, enhance electron transfer, and provide dual activation sites, leading to faster reaction rates and higher yields.^139^ Additionally, metal/metal catalysts often exhibit improved durability and reusability, making them valuable for sustainable and industrial-scale heterocycle synthesis.

**Scheme 48:**
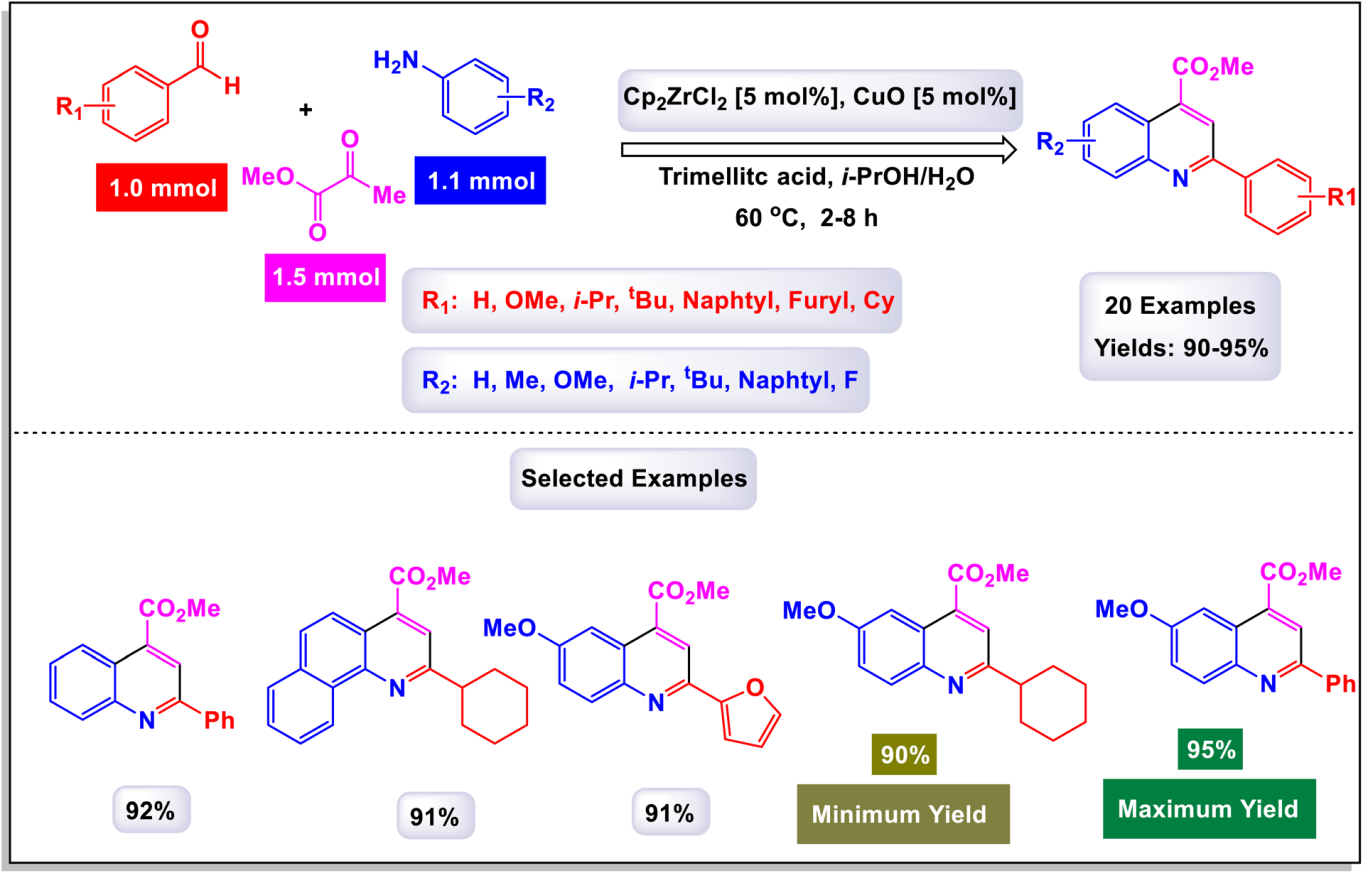
Synthesis of substituted quinolines from dicarbonyls [catalysis by Cp_2_ZrCl_2_/CuO].

**Scheme 49:**
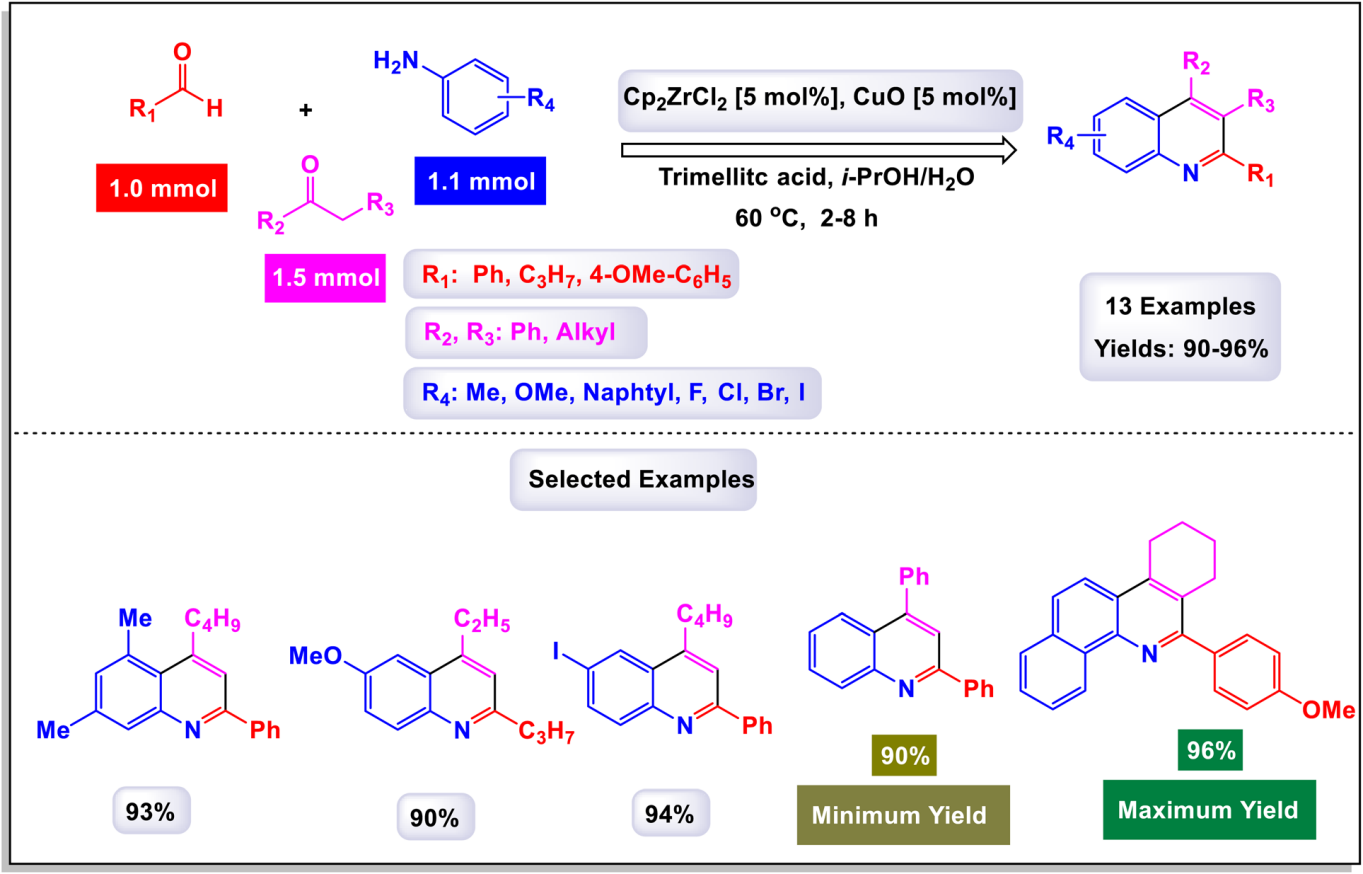
Synthesis of substituted quinolines from ketones [catalysis by Cp_2_ZrCl_2_/CuO].

**Scheme 50:**
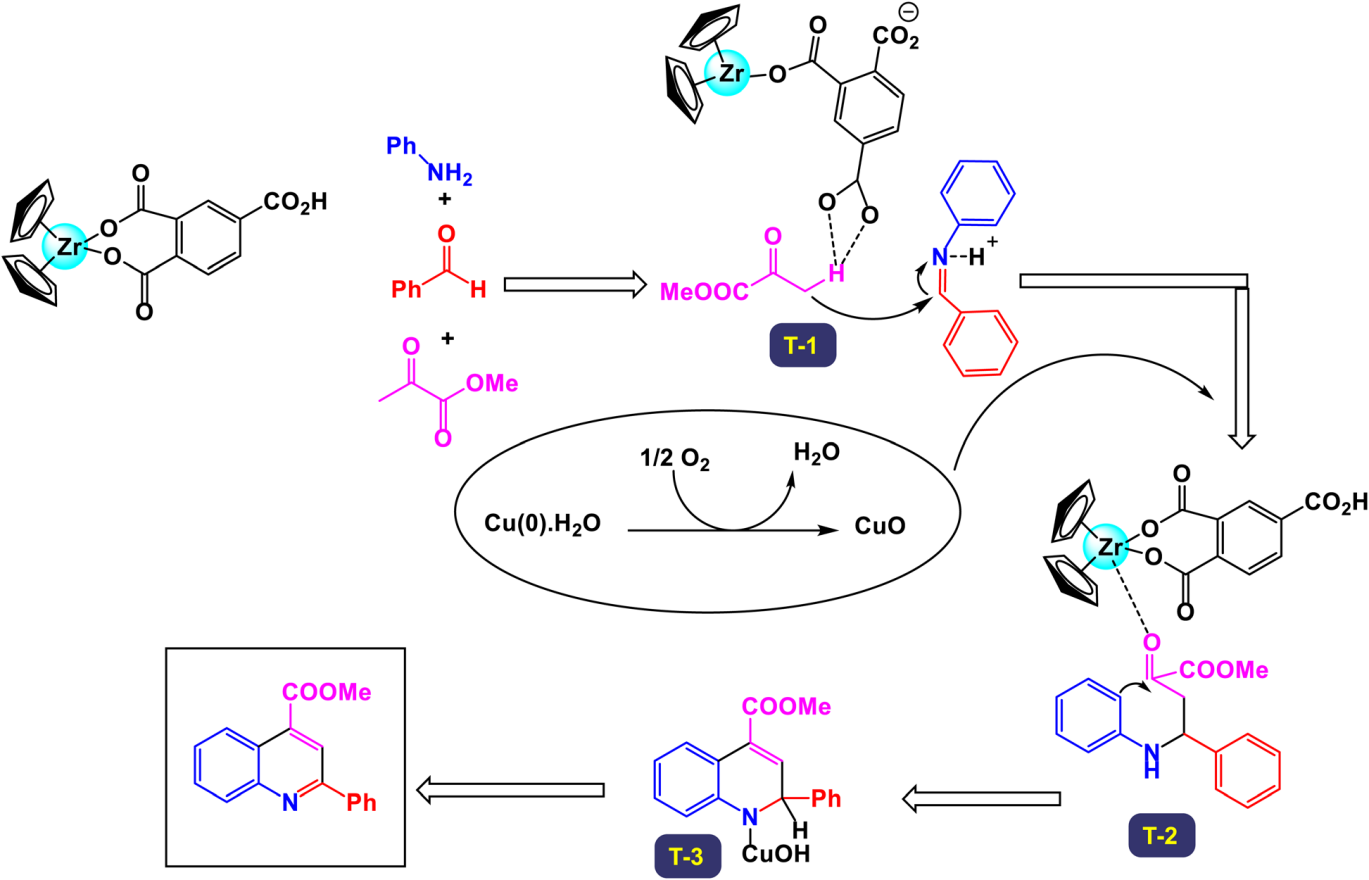
Plausible mechanism for the synthesis of substituted quinolines from dicarbonyls [catalysis by Cp_2_ZrCl_2_/CuO].

In a groundbreaking study, Sun *et al.* introduced an innovative catalytic system characterized by a unique combination of triple cooperative and relay catalysis. This sophisticated mechanism involves the Mannich addition, followed by carbon–carbon bond construction and oxydehydrogenation.^140^ Central to this process are zirconocene dichloride and trimellitic acid, which work synergistically to facilitate the Mannich addition and subsequent C–C bond formation. Meanwhile, the presence of copper oxide (CuO) enables relay catalysis for the critical oxydehydrogenation step. This cutting-edge strategy has demonstrated remarkable efficacy in synthesizing substituted quinolines from readily available starting materials, including anilines, aldehydes, and ketones. Impressively, the reaction yielded a total of 32 distinct substituted quinolines, achieving high yields ranging from 90% to 96% under mild reaction conditions, making this approach not only efficient but also practical (Schemes 48 and 49). Mechanistic studies have provided insight into this novel catalytic process, particularly through the proposed mechanism illustrated in Scheme 50. An intriguing element of this research is the formation of a zirconocene–Brønsted acid complex that is generated *in situ* and serves as the active catalyst. In this mechanism, a coordinated enolate ion engages in a targeted addition to an aldimine. This critical step is activated by a proton (H^+^) derived from one of the carboxyl groups of trimellitic acid, showcasing the cooperative functionality of this binary acid catalytic system. In a specific transition state, referred to as transition state T-2, the electron-rich aromatic ring effectively attacks the keto-carbonyl group (T-1), leading to the formation of an intermediate known as dihydroquinoline (T-3). Following this, Cu(0) in the presence of water is readily oxidized by atmospheric oxygen, regenerating CuO, which then reintegrates into the subsequent catalytic cycle. This cycle underlines the efficiency and resilience of the developed catalytic system, paving the way for future advancements in synthetic organic chemistry.

### 2.11 Catalysis by MOF

Metal–organic framework (MOF) catalysts offer unique advantages in the synthesis of heterocycles due to their high surface area, tunable porosity, and structural versatility.^141,142^ Their well-defined active sites enable selective catalysis, facilitating complex transformations such as cyclization, coupling, and C–H activation with enhanced efficiency.^143,144^ MOFs can incorporate various metal centers and organic linkers, allowing for fine-tuned catalytic properties tailored to specific heterocyclic syntheses.^143^ Additionally, their stability, recyclability, and potential for heterogeneous catalysis make them attractive for sustainable and green chemistry applications.^145^ These features contribute to high product yields, improved selectivity, and reduced waste generation in heterocycle synthesis.

**Scheme 51:**
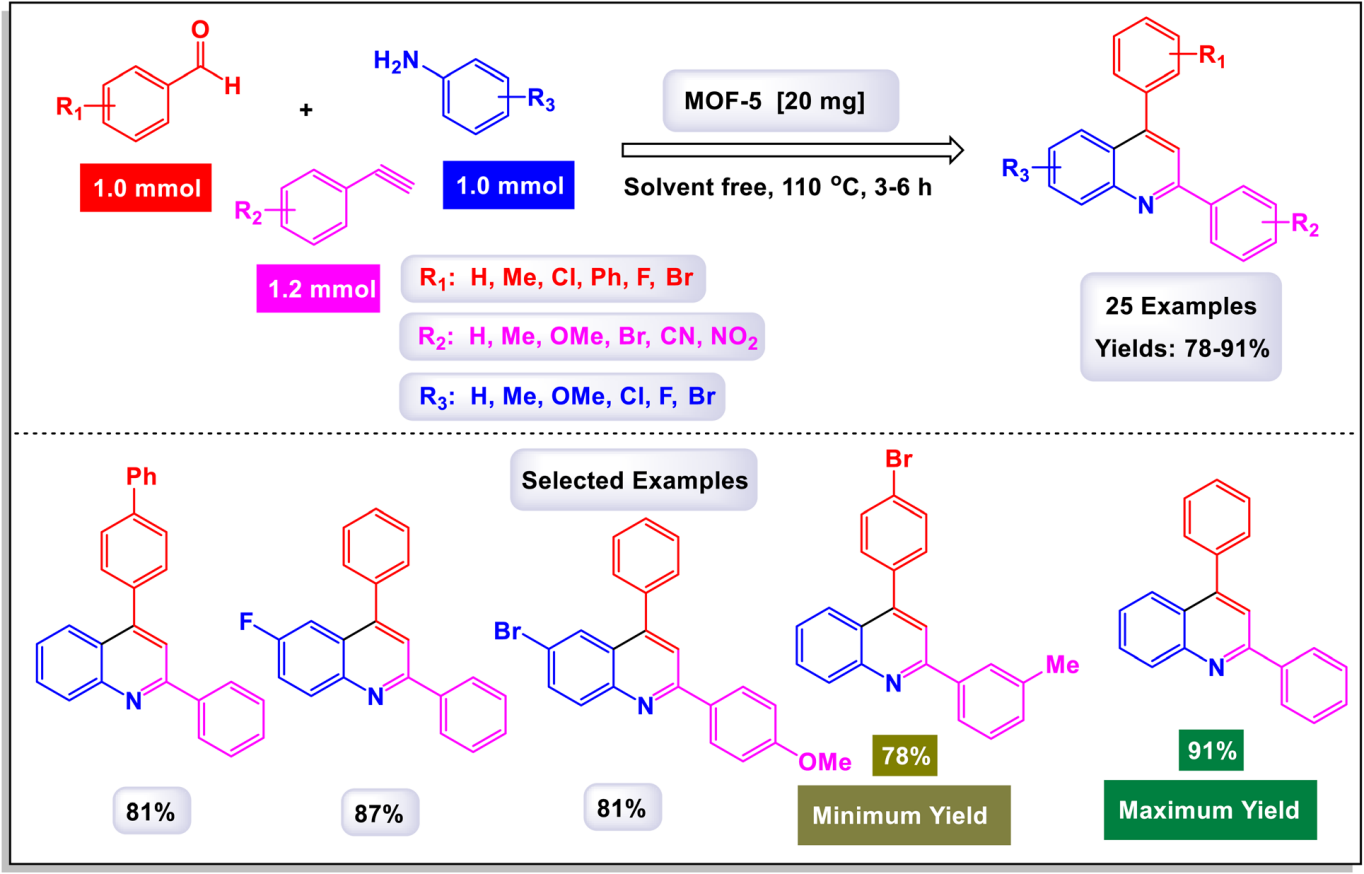
Synthesis of 2,4-disubstituted quinolines [catalysis by MOF-5].

**Scheme 52:**
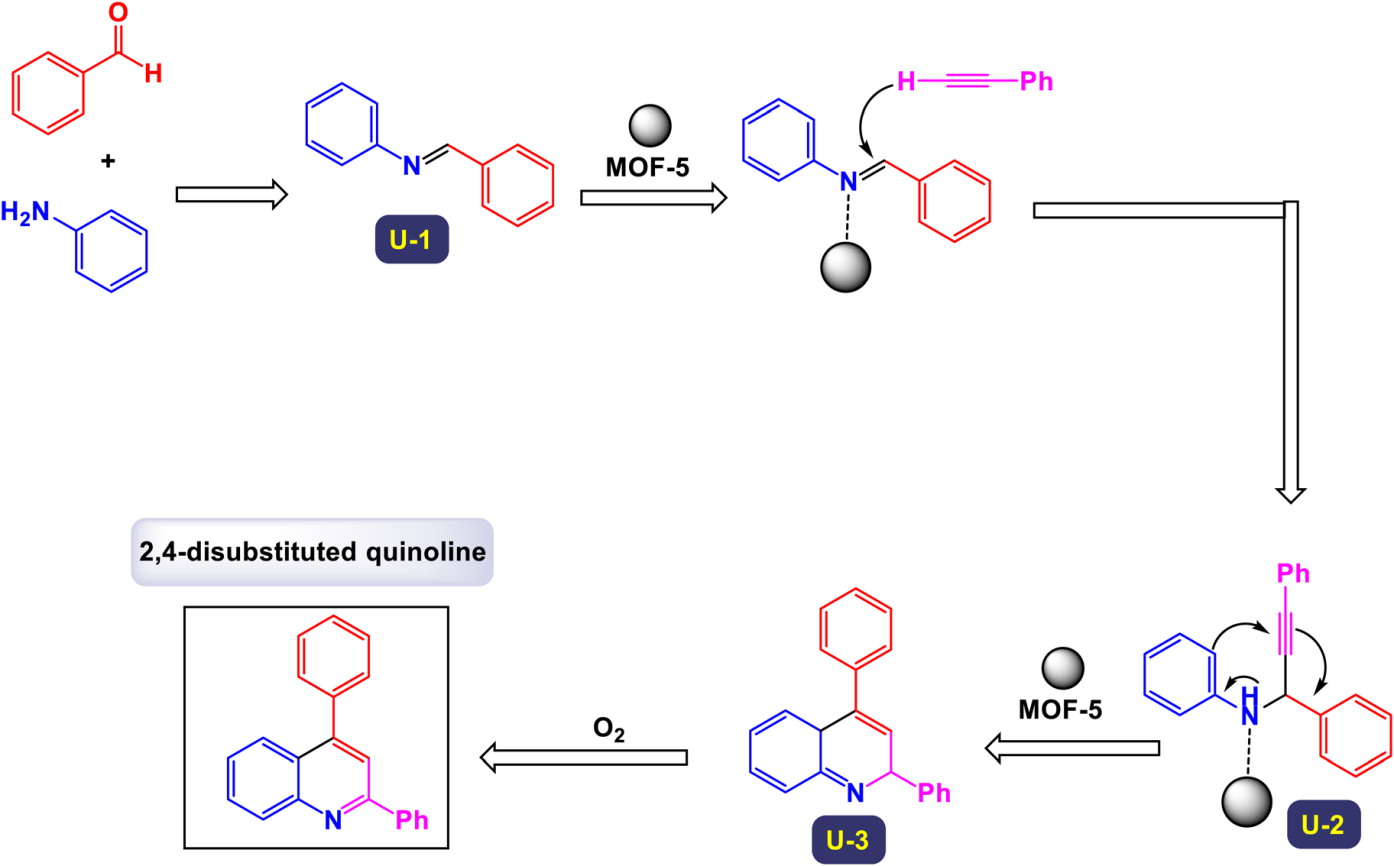
Plausible mechanism for the synthesis of 2,4-disubstituted quinolines [catalysis by MOF-5].

The research group led by Xiao has pioneered a novel three-component coupling reaction catalyzed by the MOF-5 nanocomposite, presenting an efficient method for the synthesis of 2,4-disubstituted quinoline derivatives.^143^ This innovative approach involves a one-pot reaction where an aromatic amine, an aldehyde, and an alkyne are combined, resulting in exceptional yields of the desired products. To explore the versatility of this method, various substituted phenyl acetylenes and anilines were investigated, focusing on *para*-substituents such as fluorine, chlorine, bromine, methoxy, and methyl groups. The results demonstrated that both electron-withdrawing and electron-donating substituents were generally well-tolerated, resulting in the successful synthesis of compounds featuring diverse functionalities, including alkyl, halide, and alkoxyl groups, all achieved with commendable yields (Scheme 51). Furthermore, the reaction showed compatibility with 4-ethynyl-1,1′-biphenyl, leading to the successful formation of the corresponding product. Encouraged by these promising results, the team proceeded to investigate the reactions of various substituted aromatic aldehydes and anilines under the same optimal conditions. This exploration yielded a range of quinoline derivatives in high yields. As illustrated in Scheme 52, the process begins with the *in situ* generation of intermediate (U-1) from the reaction of an aromatic aldehyde and an aniline. In this intermediate, the nitrogen atom is coordinated by the MOF-5, which enhances the electrophilicity of the resulting imine. Following this, an alkyne is introduced, leading to the formation of a propargylamine intermediate (U-2). This intermediate then undergoes an intramolecular hydroarylation of the alkyne, transitioning into a dihydroquinoline intermediate (U-3). The final step of the reaction involves the oxidation and aromatization of this intermediate using oxygen, resulting in the formation of the target quinoline compound. The MOF-5 catalyst demonstrated remarkable reusability, performing effectively up to five times with minimal loss of activity. This showcases its strong potential for practical industrial applications.

## 3 Summary and outlook

Transition metal-catalyzed multi-component reactions (MCRs) have revolutionized the synthesis of quinoline derivatives by offering efficient, selective, and sustainable methodologies. These catalysts, including palladium, copper, iron, silver, and other transition metals, facilitate key transformations such as cyclization, C–C and C–N bond formation, and oxidative coupling, enabling the rapid construction of diverse quinoline frameworks. The major advantages of these catalytic systems include high atom economy, reduced reaction steps, operational simplicity, and compatibility with a wide range of functional groups, making them highly valuable for pharmaceutical, agrochemical, and material science applications.

The highlighted advancements in transition metal catalysis have significantly improved reaction efficiency, regioselectivity, and functional group tolerance in quinoline synthesis. However, challenges remain in terms of catalyst cost, recyclability, and selectivity control, particularly for regio- and stereo-selective transformations. Future research should focus on the development of more sustainable catalysts, such as earth-abundant metals, ligand-engineered systems with enhanced activity, and hybrid catalytic approaches integrating photocatalysis and electrocatalysis. Additionally, adopting greener solvents, improving reaction conditions, and exploring continuous flow processes will further enhance the practicality and environmental impact of these methodologies.

### 3.1 Advantages of transition metal-catalyzed MCRs in quinoline synthesis

Transition metal catalysts have transformed the synthesis of quinoline derivatives through multi-component reactions (MCRs), offering several key advantages:

❖ *High efficiency and selectivity*: Transition metal catalysts enable the rapid and selective formation of quinoline frameworks by promoting key transformations such as C–C, C–N bond formation, and oxidative coupling. Their ability to control regioselectivity and stereoselectivity enhances product purity and yield.

❖ *Mild reaction conditions*: Many transition metal-catalyzed MCRs proceed under mild temperature and pressure conditions, reducing energy consumption and minimizing side reactions. This makes the synthesis process more cost-effective and environmentally friendly.

❖ *Functional group tolerance*: These catalysts exhibit broad compatibility with diverse functional groups, allowing for the direct incorporation of various substituents into the quinoline core without requiring additional protecting or deprotecting steps.

❖ *Atom economy and sustainability*: MCRs using transition metals improve atom economy by assembling multiple reactants in a single step, reducing the need for excessive reagents and minimizing waste generation. This aligns with green chemistry principles, making the process more sustainable.

❖ *Versatility and scalability*: Transition metal-catalyzed MCRs offer a wide scope of substrates and reaction pathways, making them suitable for synthesizing diverse quinoline derivatives. Their adaptability also enables scale-up for industrial and pharmaceutical applications.

### 3.2 Highlights of transition metal-catalyzed MCRs in quinoline synthesis

➢ *Copper-catalyzed approaches*: Copper catalysts excel in oxidative cyclization and C–H activation strategies, providing cost-effective alternatives to precious metals.

➢ *Iron-catalyzed green chemistry applications*: Iron, being an abundant and environmentally benign metal, has gained attention for its role in oxidative couplings and tandem reactions for quinoline synthesis.

➢ *Silver catalysis for selective oxidation*: Silver catalysts effectively promote oxidative annulation and cyclization reactions, contributing to the development of novel quinoline derivatives.

➢ *Multimetallic and hybrid catalysis*: Combining different transition metals or integrating metal catalysis with photocatalysis and electrocatalysis has opened new pathways for more efficient and sustainable quinoline synthesis.

➢ *Palladium-catalyzed strategies*: Palladium-based catalysts are extensively used in the Suzuki, Heck, and Sonogashira coupling reactions for quinoline synthesis, offering high efficiency in constructing complex frameworks.

The continuous development of transition metal-catalyzed MCRs provides a powerful and versatile platform for synthesizing quinoline derivatives with enhanced efficiency, selectivity, and environmental compatibility. Future advancements in catalyst design and reaction engineering will further expand their applications in pharmaceutical, material, and fine chemical industries. Overall, transition metal-catalyzed MCRs represent a powerful and versatile strategy for quinoline synthesis, with ongoing advancements poised to expand their scope and applicability in drug discovery and material development. The continuous evolution of these catalytic systems will play a crucial role in shaping the future of heterocyclic chemistry and sustainable synthetic methodologies.

### 3.3 Final outlook

The application of transition metal catalysts in the multi-component synthesis of quinoline derivatives has significantly advanced the field of heterocyclic chemistry, offering efficient, selective, and sustainable approaches for complex molecule construction. As the research progresses, the development of more cost-effective, earth-abundant, and recyclable catalysts will be crucial in enhancing the practicality and environmental impact of these methodologies. Additionally, integrating transition metal catalysis with emerging technologies, such as photocatalysis, electrocatalysis, and continuous flow chemistry, holds great promise for improving reaction efficiency and scalability. Future efforts should also focus on expanding the substrate scope, optimizing reaction conditions, and designing novel ligand systems to further refine selectivity and reactivity. With continued innovation, transition metal-catalyzed MCRs will remain a cornerstone for the sustainable and versatile synthesis of quinoline derivatives, driving new advancements in pharmaceuticals, materials science, and green chemistry.

## Conflicts of interest

The authors affirm that there are no conflicts of interest to disclose.

## Data Availability

No primary research results, software or code have been included, and no new data were generated or analyzed as part of this review.
